# EasyCloneMulti: A Set of Vectors for Simultaneous and Multiple Genomic Integrations in *Saccharomyces cerevisiae*

**DOI:** 10.1371/journal.pone.0150394

**Published:** 2016-03-02

**Authors:** Jérôme Maury, Susanne M. Germann, Simo Abdessamad Baallal Jacobsen, Niels B. Jensen, Kanchana R. Kildegaard, Markus J. Herrgård, Konstantin Schneider, Anna Koza, Jochen Forster, Jens Nielsen, Irina Borodina

**Affiliations:** 1 The Novo Nordisk Foundation Center for Biosustainability, Technical University of Denmark, Hørsholm, Denmark; 2 Department of Biology and Biological Engineering, Chalmers University of Technology, Göteborg, Sweden; Imperial College London, UNITED KINGDOM

## Abstract

*Saccharomyces cerevisiae* is widely used in the biotechnology industry for production of ethanol, recombinant proteins, food ingredients and other chemicals. In order to generate highly producing and stable strains, genome integration of genes encoding metabolic pathway enzymes is the preferred option. However, integration of pathway genes in single or few copies, especially those encoding rate-controlling steps, is often not sufficient to sustain high metabolic fluxes. By exploiting the sequence diversity in the long terminal repeats (LTR) of Ty retrotransposons, we developed a new set of integrative vectors, EasyCloneMulti, that enables multiple and simultaneous integration of genes in *S*. *cerevisiae*. By creating vector backbones that combine consensus sequences that aim at targeting subsets of Ty sequences and a quickly degrading selective marker, integrations at multiple genomic loci and a range of expression levels were obtained, as assessed with the green fluorescent protein (GFP) reporter system. The EasyCloneMulti vector set was applied to balance the expression of the rate-controlling step in the β-alanine pathway for biosynthesis of 3-hydroxypropionic acid (3HP). The best 3HP producing clone, with 5.45 g.L^-1^ of 3HP, produced 11 times more 3HP than the lowest producing clone, which demonstrates the capability of EasyCloneMulti vectors to impact metabolic pathway enzyme activity.

## Introduction

Budding yeast *Saccharomyces cerevisiae* is used in the biotech industry for the production of a wide range of chemicals, ranging from biofuels and bulk chemicals to nutraceuticals and pharmaceuticals [1–4]. *S*. *cerevisiae* is an excellent host for heterologous production of metabolites and fine chemicals at industrial scale, since it has a GRAS (Generally Recognized As Safe) status and is tolerant to low pH. Moreover *S*. *cerevisiae* is well-amenable to genetic engineering, so strains for novel processes can be developed relatively fast [2,5,6]. The list of chemicals that have been produced in *S*. *cerevisiae* at proof-of-concept levels counts several hundreds of compounds and includes alcohols, organic acids, terpenes [7,8], polyphenols [9,10], and glucosinolates [11], to name a few. In order to produce a chemical of interest, yeast strains are usually metabolically engineered by inserting and optimizing the required heterologous pathways as well as by improving the host properties, such as precursor and co-factor supply, tolerance to the product, or by-products formation [12]. Pathway optimization often includes balancing of the expression levels of each pathway enzyme, some of which need to be overexpressed [13]. It has been shown that higher copy number of genes causes higher level of transcription and therefore more efficient production of (heterologous) proteins [14]. However, using high copy plasmids such as 2μ-based vectors is not the preferred option, since these plasmids are not mitotically stable and thereby lead to heterogeneous gene expression in a population of cells [15,16]. Therefore, integrating genes in multiple copies into the genome to maintain stable production of the desired molecule for many generations is of high interest.

Yeast retrotransposons are a family of transposable elements that are dispersed throughout the eukaryotic genome in high numbers [17,18]. Ty elements are a subclass of retrotransposons that belong to Class-I elements, which transpose using a copy-paste mechanism with an RNA as intermediate [19]. Ty elements play a role in the mobilization of genome fragments throughout the genome, probably due to the ectopic recombination involving Ty element repetitive sequences, which can lead to chromosomal rearrangements and gene duplications in an evolutionary context [20]. Ty elements consist of two long terminal repeats (LTRs) that flank the open reading frames *TYA* and *TYB* [21]. *S*. *cerevisiae* has five distinct families of Ty elements, designated Ty1 to Ty5. Ty1, Ty2, Ty4 and Ty5 are Ty1-*copia* group elements, while Ty3 is a Ty3-*gypsy* retrotransposon [22,23]. A total of 331 Ty insertions have been identified in the *S*. *cerevisiae* genome, 85% of which are LTR fragments or solo LTRs [23]. Ideally, target sites for genomic integrations in multiple copies would be present in high numbers and would preferably be spread across the entire genome to limit the risk of loss of DNA material by homologous recombination, as target sites in this case will most probably be interspaced by elements essential for growth. Therefore, LTRs represent potential candidates as target sites for multiple integrations.

Sequences from Ty retrotransposons have previously been used as target sites for gene integration. Sakai *et al*. used the sequence of the terminal repeats, δ sequence, of a Ty element as recombination sites to express heterologous genes and reported three to five insertions, on one particular chromosome. Recombinant protein production increased significantly and the integrations were mitotically stable over a period of 50 generations [24]. Parekh *et al*. constructed yeast integration vectors carrying the NEO^R^ resistance marker that targets Ty LTR sequences and reported tandem integrations ranging from one to 30 copies. Thereby, secretion levels of bovine pancreatic trypsin inhibitor were significantly increased. This system could be tuned by applying different concentrations of the antibiotic G418 [25]. A flocculent *S*. *cerevisiae* strain was engineered to stably secrete β–galactosidase by integrating the β–galactosidase expression cassette at δ sequences of the retrotransposon Ty1 [26]. Integrants producing the highest levels of β–galactosidase were shown to carry approximately eight gene copies [26]. δ sequences have also been successfully applied to metabolic pathway engineering. For the production of 1,2-propanediol, Lee *et al* engineered a strain with increased copy numbers of two heterologous genes, *mgs* and *gldA*, integrated at δ sequences [27]. In this case, the strain containing three copies of *mgs* and *gldA* produced the highest amount of 1,2-propanediol, although a clear correlation with copy number was not apparent [27]. Even though the abovementioned studies reported about integrations that proved stable for their copy number, most of the integrations occurred at a limited number of sites and mostly as tandem arrays. This likely owes to the fact that the vectors for integration were designed for single cross-over type integrations. More recently, Yamada *et al* used a novel approach, “cocktail δ-integration”, to optimize expression levels of cellulolytic enzymes [28], while Yuan and Ching used δ sequences to successfully assemble and integrate multi-step pathways in *S*. *cerevisiae* [29]. In these two studies, gene conversion events are expected as mechanism for integration. Ribosomal DNA, with 100–200 tandem repeats, was also identified as a potential target sequence for multiple integrations: Lopes *et al* reported 100–200 stable integrations using rRNA homology arms in combination with the deficient *LEU2-d* selection marker in order to promote multiple integration events [30].

In this study, we utilized the possibilities offered by the five families of Ty elements in *S*. *cerevisiae* and specifically the sequence diversity of their LTRs to create a new set of multicopy integrative vectors, named EasyCloneMulti. This vector set targets different subsets of Ty sequences in *S*. *cerevisiae* genome. Like the previously described single integration EasyClone vectors from which they are derived, EasyCloneMulti vectors are designed to integrate and replace the targeted integration loci via a gene conversion type mechanism and offer the same possibilities as the EasyClone vectors in terms of uracil-excision based cloning of up to two genes with uni- or bidirectional promoters of choice [11,16,31]. Multicopy integration and protein expression levels were analyzed using green fluorescent protein (GFP) as a reporter. As a further proof of concept, we applied EasyCloneMulti to engineer the rate controlling enzymatic step, catalyzed by L-aspartate-α-carboxylase, of the β–alanine-dependent metabolic pathway to produce 3-hydroxypropionic acid (3HP) [32].

## Materials and Methods

### Strains

*Escherichia coli* DH5α was used for cloning procedures. *S*. *cerevisiae* strains are listed in S2 Table.

### Media

Drop-out media and agar plates were prepared using pre-mixed drop-out powders from Sigma-Aldrich. Mineral medium was prepared as described previously [16]. Synthetic fed-batch medium for *S*. *cerevisiae* M-Sc.syn-1000 was purchased from M2P labs GmbH (Germany). The medium was supplemented with the supplied vitamins solution (final 1% v/v) and the enzyme mix (final concentration 0.5% v/v) immediately prior to use.

### Identification of consensus sequences for the different Ty families of *S*. *cerevisiae*

All Ty sequences reported on the genome of *S*. *cerevisiae* S288C were retrieved from the Saccharomyces Genome Database (www.yeastgenome.org). All sequences labeled as LTR and larger than 200 nucleotides were uploaded to ClustalW2 to perform a multiple sequence alignment (www.ebi.ac.uk/Tools/msa/clustalw2). From the resulting alignment, five consensus sequences were defined: Ty1Cons1, Ty1Cons2, Ty2Cons, Ty3Cons and Ty4Cons (S1 Table). The consensus sequences were then included in the input file together with all Ty sequences of more than 200 nucleotides and a new alignment was performed. From the output of the alignment, a phylogenetic tree was constructed using the maximum likelihood method for tree building provided by the software MEGA6 [33].

### Construction of the EasyCloneMulti vectors

#### Construction of the EasyCloneMulti vector based on Ty4 cons

All primers used in this study are listed in S3 Table, all vectors of this study are listed in S4 Table. The DNA strings for consensus sequences were purchased as gBlocks from IDT Integrated DNA Technology. Initially, a Ty4Cons bearing vector, pCfB312, was constructed in two subsequent In-Fusion cloning events, and as follows: pCfB255 was PCR amplified using primers PR-512 and PR-513, resulting in a pCfB255 linear fragment devoid of the “upstream” region for homologous recombination. In parallel, the gBlock containing Ty4 consensus sequence was PCR amplified using PR-517 and PR-518, in order to generate the upstream Ty4 sequence for homologous recombination. Amplifications were carried out using Phusion DNA polymerase (Finnzymes, now Thermo Fisher Scientific) and recommended conditions. Resulting PCR products were treated with *Dpn*I and gel-purified according to manufacturer’s recommendations (Macherey-Nagel, NucleoSpin Gel and PCR clean-up). The two PCR fragments share homologous regions at their 5’ and 3’ ends so that the In-Fusion reaction can be carried out according to the manufacturer’s recommendations (ClonTech Laboratories Inc.). After In-Fusion, 2.5 μL of the In-Fusion mix (total reaction volume of 5 μL) was transformed into 100 μL of chemically competent *E*. *coli* DH5α cells [34]. Correct vectors were isolated and verified by restriction digest. The resulting vector was used as template for a PCR reaction using primers PR-514 and PR-515, resulting in a linear fragment devoid of the “downstream” region for homologous recombination. In parallel, the gBlock containing Ty4 consensus sequence was PCR amplified using PR-519 and PR-526, in order to create the downstream Ty4 sequence for homologous recombination. Amplifications were carried out using Phusion DNA polymerase, reactions were then treated by *Dpn*I and gel-purified. Like above, the two latter PCR fragments share homologous regions at their 5’ and 3’ ends so that In-Fusion reaction can be performed. Correct vectors corresponding to vector pCfB312, bearing an EasyClone USER cassette and a *Kluyveromyces lactis URA3* selection marker (*Kl*.*URA3*) surrounded by upstream and downstream regions for homologous recombination at LTR from the Ty4 family, were verified by restriction digest.

*Kl*.*URA3* on pCfB312 was further modified by the addition of a degradation signal for quicker degradation of the Ura3 protein [35]. This was done by amplifying pCfB312 using primers PR-521 and PR-522 using PfuX7 [36]. After *Dpn*I treatment, separation and gel-purification, the fragment was circularized on itself in a uracil-excision reaction mediated by the USER enzyme mix (New England Biolabs). The resulting vector was pCfB322, which has already been used in a metabolic study by Borodina et al. [32]. pCfB322 bears an EasyClone USER cassette and a *Kluyveromyces lactis URA3* selection marker fused to the degradation signal (*Kl*.*URA3-degradation signal*) surrounded by upstream and downstream regions for homologous recombination at LTR from the Ty4 family.

As reporter, a GFP expression cassette comprising the *S*. *cerevisiae TEF1* promoter and the coding sequence for the green fluorescent protein was cloned using the classical uracil excision protocol reported previously [16]. The GFP-encoding sequence was prepared for uracil excision cloning by PCR amplification using primers PR-311 and PR-312 and PfuX7 [36]. Similarly, the GFP expression cassette was cloned into pCfB312, pCfB322, pCfB255, pCfB054 resulting into vectors pCfB321, pCfB326, pCfB329 and pCfB319.

#### EasyCloneMulti vectors bearing consensus sequences targeting different families of Ty sequences

pCfB326, bearing the p*TEF1*-GFP reporter cassette and the *Kl*.*URA3-degradation signal* as selective marker surrounded by upstream and downstream regions for homologous recombination at LTR from the Ty4 family, was used as template to generate four new vectors targeting LTRs of the Ty1, Ty2 or Ty3 family of Ty elements for integration. As two different consensus sequences for the Ty1 family were selected (Ty1Cons1 and Ty1Cons2), we constructed two vectors, pCfB1136 and pCfB1137 for the Ty1 family.

The overall principle was that the upstream and the downstream regions for homologous recombination at Ty4 type of LTRs, borne on pCfB326, were exchanged in a single uracil-excision based reaction for upstream and downstream regions for LTRs of the other families, i.e. Ty1, Ty2 and Ty3. In order to do this, two PCR fragments were generated from pCfB326: 1) using primer set PR-1700/PR-1702 and 2) using PR-1698/PR-1704. These two fragments encompass all features of pCfB326 but the upstream and downstream regions for homologous recombination at Ty4 elements. Upstream regions for homologous recombination at Ty1, Ty2 and Ty3 elements were generated by PCR using primers PR-1697/PR-1699 and gBlock templates Ty1Cons1, Ty1Cons2, Ty2Cons, TY3cons, respectively. Downstream regions for homologous recombination at Ty1, Ty2 and Ty3 elements were generated by PCR using primers PR-1701/PR-2051 and gBlock templates Ty1Cons1, Ty1Cons2, Ty2Cons, Ty3cons, respectively. After uracil-excision reaction and transformation of chemically competent *E*. *coli* DH5α, the following vectors were obtained: pCfB1136, pCfB1137, pCfB1138, pCfB1139.

The construction of EasyCloneMulti vectors containing *panD*, the cloning of EasyCloneMulti plasmids with synthetic *Kluyveromyces lactis Kl*.*URA3*, *Schizosaccharomyces pombe Sp*.*HIS5* and *Kluyveromyces lactis Kl*.*LEU2* markers, as well as the construction of a complete set of USER cassette containing EasyCloneMulti vectors are explained in detail in the Supplementary Methods (S1 File). All these additional EasyCloneMulti vectors are listed in S4 Table.

### Transformation of *S*. *cerevisiae*

*S*. *cerevisiae* was transformed with different vectors using the lithium acetate transformation protocol [37]. Prior to transformation, integrative vectors were digested by *Not*I and column-purified (NucleoSpin Gel and PCR cleanup kit, Macherey Nagel). Approximately 1 μg DNA was transformed into competent yeast cells. The cells were selected on drop-out agar medium.

### Measurement of fluorescence on a microtiter plate reader

Colonies of *S*. *cerevisiae* strains to be tested were inoculated in 0.8 mL of drop-out medium in a 96 deep-well plate with air-penetrable lid (EnzyScreen, NL). After approximately 24 hours of cultivation at 30°C with 300 rpm in deep-well plate, a fresh 96 deep-well plate was inoculated from the first cultivation plate to an initial OD_600_ = 0.05. After cultivation for 36–48 hours at 30°C with 300 rpm agitation, fluorescence (λ_Excitation_ 485nm, λ_Emission_ 515nm) and OD_600_ were measured on a microtiter plate reader BioTek Synergy MX (BioTek). Results are reported as specific fluorescence, which is obtained by dividing measured average fluorescence by the measured OD_600_.

### Single cell measurements of fluorescence

*S*. *cerevisiae* strains were inoculated from solid drop-out medium lacking uracil into 24 deep-well plates (EnzyScreen, NL) containing 2 mL liquid drop-out medium without uracil for overnight cultivations at 30°C with 300 rpm. The next day, a fresh 24 deep-well plate, containing 2 mL per well of drop-out medium without uracil, was inoculated to an initial OD_600_ of 0.05. After approximately 24 hours of cultivation at 30°C with 300 rpm agitation, the cells were harvested and fixed with paraformaldehyde according to the following protocol. 1.5 mL samples were taken and immediately cooled in ice-water bath and subsequently centrifuged at 4°C, 2000 x g for 2 min. Supernatant was removed and pellet resuspended in 200 μL of 2% paraformaldehyde. The mix was incubated on ice for 1 hour and subsequently centrifuged at 4°C, 2000 x g for 2 min. Finally, the paraformaldehyde was removed and the cell pellet was resuspended in 200 μL PBS. The fixed cells were stored at 4°C until further analysis.

Cells were analyzed on a BD FACSAria (BD Biosciences) equipped with three solid state diode lasers: air-cooled CoherentTM SapphireTM solid-state diode laser (488 nm, 100 mW), air-cooled CoherentTM Yellow Green laser (561 nm, 100 mW), and an air-cooled CoherentTM Deep Blue laser (445 nm, 50 mW). The FITC-A filter was applied for measurement of green fluorescence. Flow cytometry data were analyzed and interpreted using Cyflogic software (www.cyflogic.com/).

### Cultivation of *S*. *cerevisiae* for the production of 3HP

For each strain, at least sixteen single colonies originating from independent transformants were inoculated in 0.5 mL drop-out liquid medium without uracil, histidine, and leucine in a 96 deep-well plate with air-penetrable lid (EnzyScreen, NL). Plates were incubated at 30°C with 300 rpm agitation at 5.1 cm orbit cast overnight (Eppendorf innova 44). 20 μL of the overnight cultures were used to inoculate synthetic fed-batch medium (Feed-In-Time fed-batch medium, m2p-Labs) in a 96 deep-well plate. Fermentation was carried out for 72 hours at the same conditions as above.

At the end of the cultivation OD_600_ was measured using the microtiter plate reader BioTek Synergy MX (BioTek). The culture broth was filtered (Acroprep 0.2 μm, Supor membrane, VWR) and the supernatant analyzed for 3-hydroxypropionic acid concentration on HPLC. For measuring 3HP on HPLC, 30 μL of the sample was analyzed for 30 min using an Aminex HPX-87H ion exclusion column with a 1 mM H_2_SO_4_ flow of 0.6 mL.min^-1^. The temperature of the column was 60°C. The refractive index at 45°C and the UV absorption at 210 nm were recorded. 3HP quantification was performed based on RI chromatograms. The UV spectrum was recorded with a diode array and the identity of 3-hydroxypropionic acid was additionally verified by comparison with the spectrum of a commercial standard.

### Genomic DNA extraction and library sequencing

Genomic DNA was extracted using QIAamp DNA Mini Kit (QIAGEN, Germany). The genomic libraries were generated using the TruSeq Nano DNA LT Library Prep Kit (Illumina Inc., San Diego CA). Briefly, 100 ng of genomic DNA diluted in 52.5 μL TE buffer was fragmented in Covaris Crimp Cap microtubes on a Covaris E220 ultrasonicator (Woburn, MA) with 5% duty factor, 175 W peak incident power, 200 cycles/burst, and 50-s duration under frequency sweeping mode at 5.5 to 6°C (Illumina recommendations for a 350-bp average fragment size). The ends of fragmented DNA were repaired by T4 DNA polymerase, Klenow DNA polymerase, and T4 polynucleotide kinase. The Klenow exo minus enzyme was then used to add an 'A' base to the 3' end of the DNA fragments. The adapters were ligated to the ends of the DNA fragments, and the DNA fragments ranging from 300–400 bp were recovered by beads purification. Finally, the adapter-modified DNA fragments were enriched by 3 PCR cycles. Final concentration of each library was measured by Qubit 2.0 Fluorometer and Qubit DNA Broad range assay (Life Technologies). Average dsDNA library size was determined using the Agilent DNA 7500 kit on an Agilent 2100 Bioanalyzer. Libraries were normalized and pooled in 10 mM Tris-Cl, pH 8.0, plus 0.05% Tween 20 to the final concentration of 10 nM. Denaturated in 0.2N NaOH, 10 pm pool of 20 libraries in 600 μL ice-cold HT1 buffer was loaded onto the flow cell provided in the MiSeq Reagent kit v2 (300 cycles) (Illumina Inc., San Diego CA) and sequenced on a MiSeq (Illumina Inc., San Diego CA) platform with a paired-end protocol and read lengths of 151 nt.

### Next generation sequencing data analysis

Illumina reads were aligned to both *S*. *cerevisiae* S288C (www.yeastgenome.org/strain/S288C/overview) and CEN.PK113-7D (www.ncbi.nlm.nih.gov/assembly?LinkName=bioproject_assembly_all&from_uid=52955) reference genomes as well as relevant integrative vector sequences (see S1 File) using bowtie2 [38]. Copy numbers for integrated genes (GFP, *Kl*.*URA3* marker) as well as other sequence elements present on the vectors (e.g. left and right consensus sequences used for homologous recombination) were determined by taking the ratio of read coverage across these elements to the average coverage for coding regions in the chromosomal DNA sequences. The coverage (the depth of sequencing) was calculated using coverageBed tool in bedtools [39]. Potential integration loci were determined by taking advantage of paired end sequencing as follows using samtools [40] and custom Python scripts: First, reads that mapped uniquely into regions on the integration construct that do not have significant similarity to regions on the *S*. *cerevisiae* genome were identified. Second, the corresponding paired end reads to the reads found in step #1 were identified. Third, if these paired ends mapped (even partially) to the reference genomes, the regions with significant paired end read enrichment were further examined manually using Tablet [41]. Potential tandem duplications were identified by manual inspection of read mappings against vector sequences using Tablet.

## Results

### Identification of consensus sequences for multiple integrations

LTRs of Ty retrotransposons are scattered in multiple copies throughout the entire genome of *S*. *cerevisiae* and are present on all chromosomes. Therefore, they are attractive target sequences for vectors aiming at multiple integrations in *S*. *cerevisiae*. Integration at Ty sequences was previously reported and successfully used to trigger multiple integrations of DNA constructs into the genome [24,25,27]. However, it was not shown whether it is possible to define specific DNA sequences that would trigger the insertion of a DNA construct into a subset of Ty sequences only. Importantly, the different Ty families are present in different numbers, which could potentially be exploited to achieve different ranges of expression levels. Therefore, we first investigated whether we could identify consensus sequences amongst reported LTR sequences that would be specific to a certain subset of Ty sequences belonging to the same family. These sequences should preferably share a high degree of identity with a maximum number of LTRs belonging to the same family while being highly dissimilar to sequences belonging to other families.

**Fig 1:**
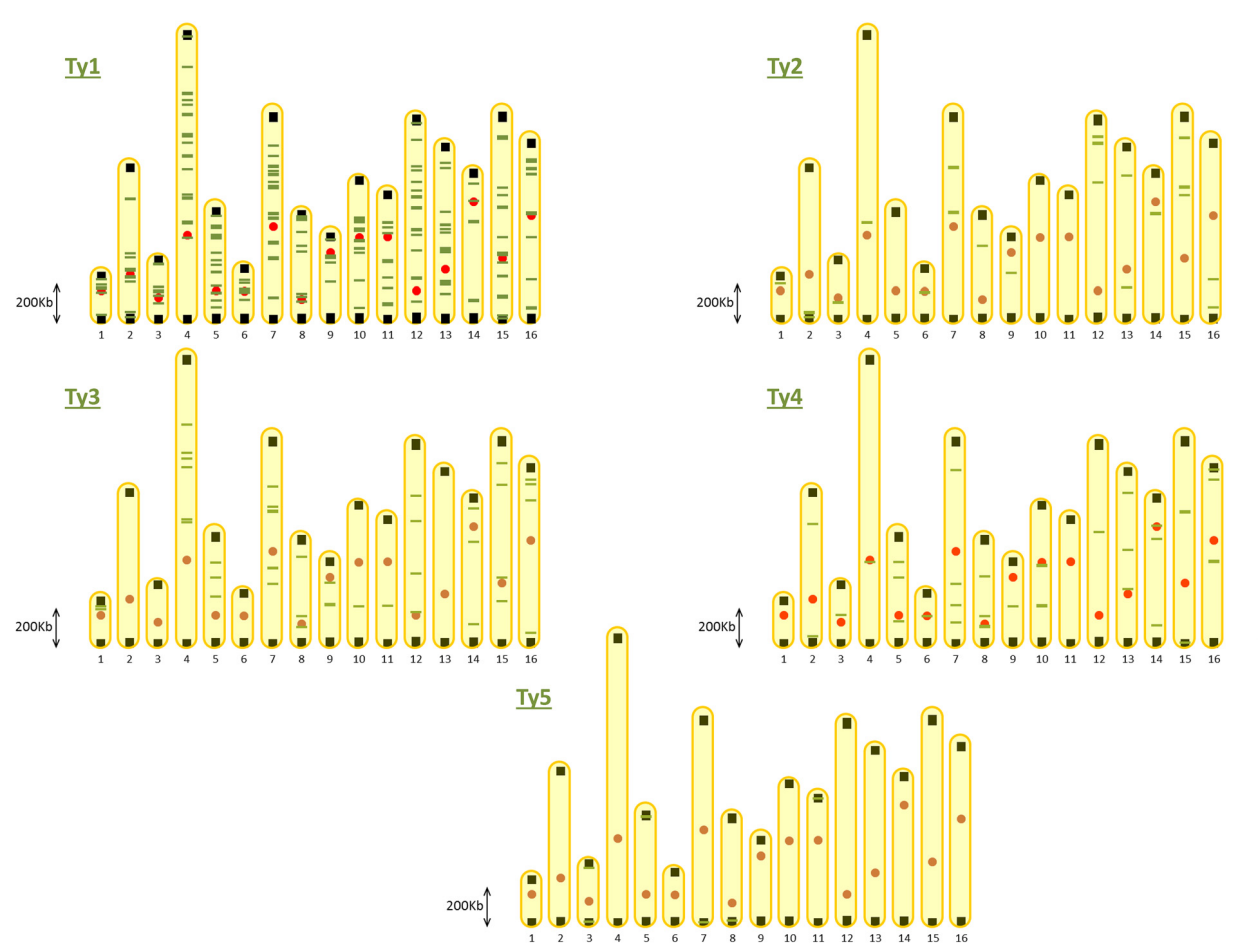
Chromosomal distribution of Ty sequences on *S*. *cerevisiae* S288C genome. Ty sequences and their location were downloaded from www.yeastgenome.org. Green bars: Ty sequences, red circles: centromeres, black squares: telomeres.

The distribution of the different Ty sequences on the genome of *S*. *cerevisiae* is presented in Fig 1. The Ty1 family is the largest one with 217 members scattered throughout the entire genome (Fig 1) [23]. An interesting feature of all families, besides Ty5, is that they consist of members distributed on most of the chromosomes. Ty2, Ty3 and Ty4 sequences appear particularly appealing as, although consisting of fewer members than the Ty1 family, these elements are largely spread on all chromosomes and only a small fraction of members of each family is present on the same chromosomes (Fig 1). With the objective of creating a vector toolset for multiple and stable integrations, this would be valuable to reduce the possibility of integrations close to each other on the same chromosome, as this could lead to genomic instability of integrated DNA sequences triggered by homologous recombination. Indeed, integration sites for the single integrative vector toolset EasyClone were defined in a way that integration sites are interspersed by essential genes, thereby limiting the risk of losing DNA material by homologous recombination [11,16]. Ty sequences of the Ty5 family were not considered suitable for the vector system presented here, as most of these elements are located in telomeric regions (Fig 1).

**Fig 2:**
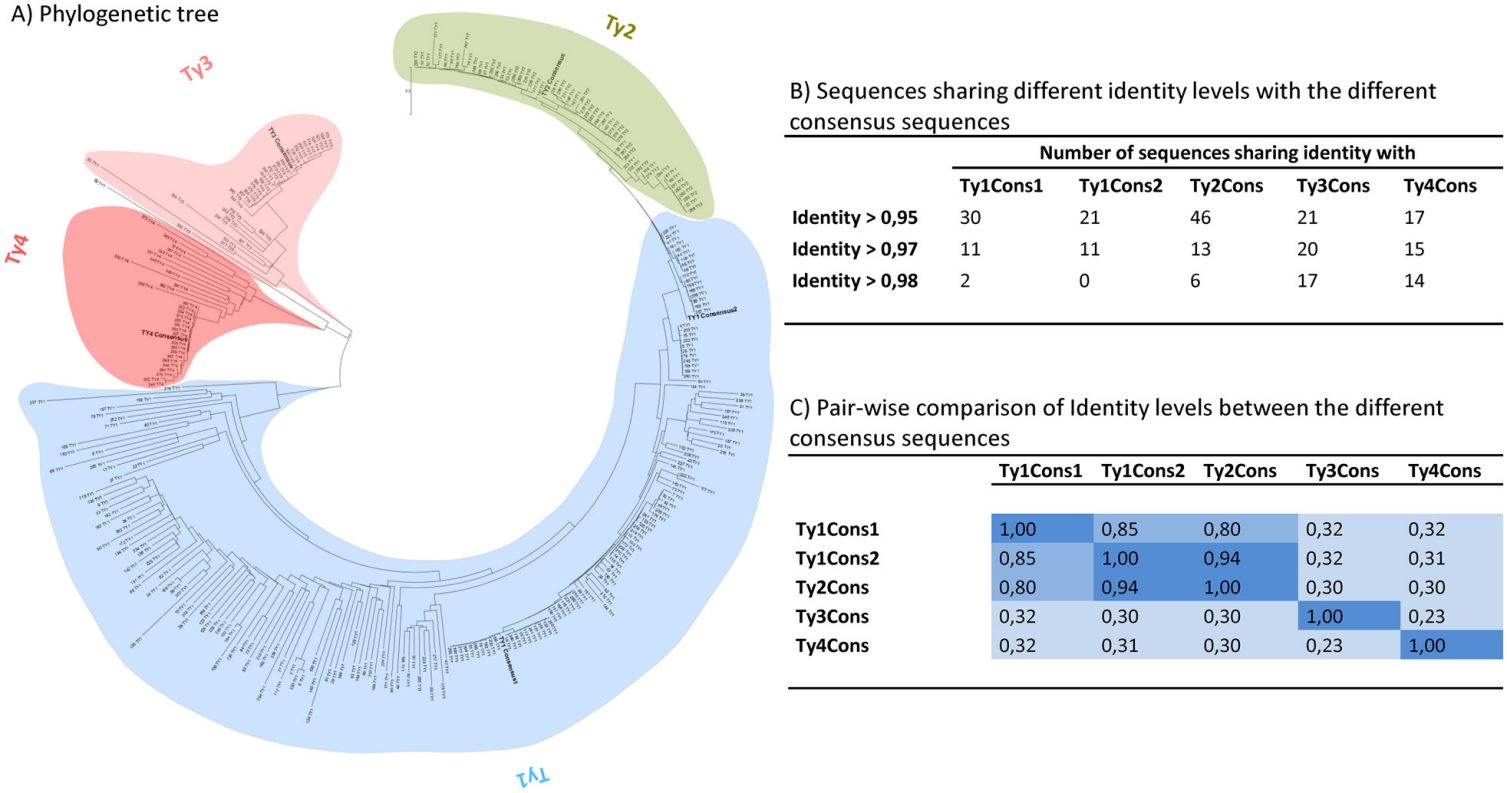
Phylogenetic tree based on alignments of LTRs from Ty elements of *S*. *cerevisiae* S288C. LTRs longer than 200 nucleotides were retrieved and a multiple alignment was performed at www.ebi.ac.uk/Tools/msa/clustalw2. A) A phylogenetic tree was created using the maximum likelihood method for tree building provided by the software MEGA6. The main cluster for each Ty family is highlighted blue (Ty1), green (Ty2), light pink (Ty3) or dark pink (Ty4). Five consensus sequences—Ty1Cons1, Ty1Cons2, Ty2Cons, Ty3Cons, and Ty4Cons—are specified in bold letters. B) Per Ty consensus sequence, the number of genome sequences sharing >0.95, >0.97 or >0.98 level of identity is reported. C) Pair-wise comparison of identity levels between consensus sequences.

Five LTR consensus sequences were defined as reported in the material and methods section: Ty1Cons1, Ty1Cons2, Ty2Cons, Ty3Cons and Ty4Cons. The phylogenetic tree relating the five consensus sequences to the other Ty sequences of *S*. *cerevisiae* is presented in Fig 2. In general, it can be observed that LTR sequences belonging to either of the families Ty2, Ty3 or Ty4, cluster significantly together in one main cluster with few divergent sequences (Fig 2A). Especially, Ty3Cons and Ty4Cons share high identity levels with sequences of their respective families. In the case of Ty3Cons, 21 sequences showed identity levels over 95% within the same family, and 17 out of these 21 showed over 98% of identity. In the case of Ty4Cons, 14 out of 17 sequences have identity levels higher than 98% (Fig 2B). Interestingly, cluster Ty2 is the only cluster that consists of sequences originating from different families as both Ty2- and Ty1-labeled sequences are present in that cluster, at an approximate 60/40 ratio (Fig 2A). The Ty1 family can be divided into four main clusters, although a high number of sequences belonging to it are highly divergent (Fig 2A). Two consensus sequences were therefore defined for this family as Ty1Cons1 and Ty1Cons2. Ty1Cons1 and Ty1Cons2 share only 85% of identity with each other and locate at two different Ty1 family clusters (Fig 2C). Ty1Cons1 shares more than 95% identity with 30 Ty1 sequences, while Ty1Cons2 shares the same degree of identity with 21 Ty1 sequences.

The five different consensus sequences defined in this analysis are also characterized by a low level of identity to each other, with exception of Ty2Cons and Ty1Cons2 (Fig 2C). Ty3Cons and Ty4cons are highly dissimilar with a maximum of 32% identity to the other consensus. While the highest identity level is observed for Ty2Cons and Ty1Cons2 with 94% of identity, those sequences are still dissimilar with the other consensus sequences defined (Fig 2C). In conclusion, we assume that the different consensus sequences defined here are both identical enough to certain subsets of Ty sequences and dissimilar enough between each other to trigger multiple integrations at specific subsets of Ty sequences. These consensus sequences are the basis for constructing EasyCloneMulti vector backbones for multiple insertions.

### The main features of EasyCloneMulti vectors

**Fig 3:**
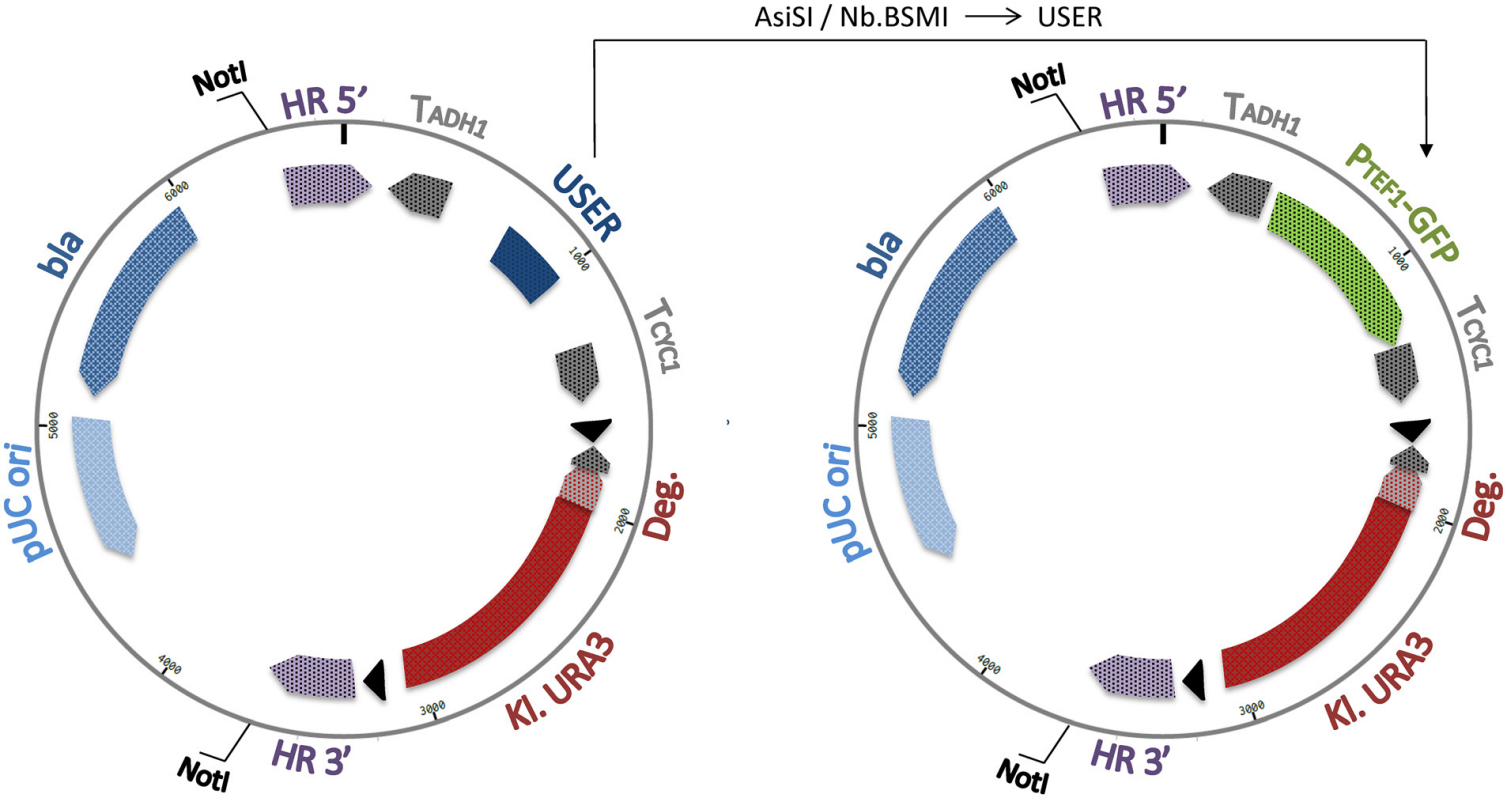
Functional map of the EasyCloneMulti vectors. Two types of EasyCloneMulti were constructed: A) EasyCloneMulti with a USER cassette for cloning purposes and B) EasyCloneMulti with a reporter cassette consisting of P_*TEF1*_-GFP-T_*CYC1*_. All EasyCloneMulti vectors share the following features: 1) upstream and downstream regions for insertion at specific Ty sequences, HR 5’ and HR 3’, flanked by *Not*I restriction sites for linearization 2) a selective marker, i.e. *URA3* from *Kluyveromyces lactis*, flanked by loxP sites; 3) an in-frame fusion of the marker gene with a degradation signal. HR 5’ and HR 3’ are in direct orientation with respect to one another to trigger gene conversion based integrations. EasyCloneMulti with a USER cassette support the same USER cloning possibilities as EasyClone vectors [16].

**Fig 4:**
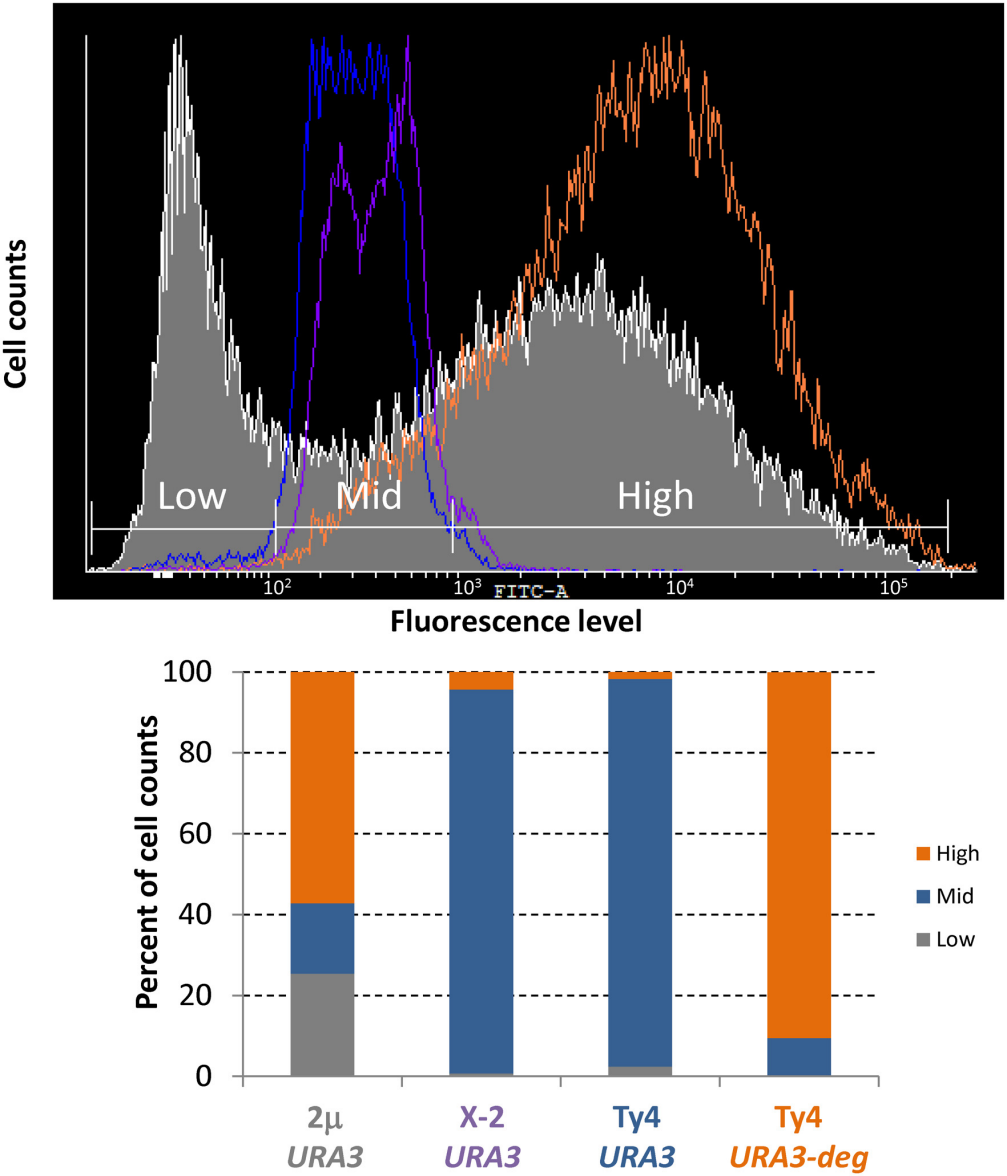
Comparison of single cell fluorescence levels of *S*. *cerevisiae* strains bearing different types of vectors. Single cell measurements of fluorescence of strain CEN.PK 113-5D transformed with either of the following vectors: pCfB319 (episomal, 2μ, *URA3*), pCfB329 (single integrative at locus Chr. X-2, *URA3*), pCfB321 (multi-integrative, Ty4Cons based, *URA3*) and pCfB326 (multi-integrative, Ty4Cons, fusion *URA3*-degradation signal). Low (grey), mid (blue) and high (orange) expression levels were arbitrarily defined and are represented.

EasyCloneMulti vector backbones for multicopy integrations were constructed based on the EasyClone vector series previously reported [16]. Each of the EasyCloneMulti vectors comprises the following features (Fig 3): i) an upstream and downstream region for insertion at a specific subset of Ty sequences, HR 5’ and HR 3’, ii) a selective marker, i.e. *URA3* from *Kluyveromyces lactis* (*Kl*.*URA3*), iii) flanking loxP sites for recycling the selective marker, iiii) a USER cloning site (*Asi*SI/*Nb*.*Bsm*I). HR 5’ and HR 3’ correspond to the 5’ and the 3’ end of each of the LTR consensus sequences (S1 Table). Their size varies between 164 and 186 bp. HR 5’ and HR 3’ are in direct orientation with respect to one another in order to trigger integration via a gene conversion type mechanism. In every EasyCloneMulti vector, a further modification was added, i.e. the in-frame fusion of *URA3* with a degradation signal leading to the quick degradation of the Ura3 protein [35,42]. The signal for degradation is a DNA sequence encoding the following amino acid sequence: ACKNWFSSLSHFVIHL. This C-terminal extension is presumed to contain degradation signals channeling proteins to the ubiquitin system. Therefore, we chose to fuse *Kl*.*URA3* to the degradation signal CL-1, as a fusion to this particular C-terminal sequence was shown to result in rapid degradation of the Ura3 protein [35]. The latter feature proved to be essential for triggering insertions at multiple loci (Fig 4). Similar to EasyClone vectors, EasyCloneMulti vectors can be linearized by digestion with *Not*I and then transformed into *S*. *cerevisiae*.

### Combining a Ty4 targeting consensus sequence and a fast degrading selective marker leads to high expression levels of green fluorescent protein

Fluorescence levels of GFP arising from the EasyCloneMulti vector bearing the Ty4 consensus sequence were compared to the same vector backbone bearing the Ty4 consensus sequence but devoid of the signal for degradation of *Kl*.*URA3*, to an episomal vector, and to a single integrative vector targeting locus 2 on chromosome X (an integration site described by [11]). Single cell fluorescence levels were measured and analyzed (Fig 4). We initially observed that fluorescence levels of cells harboring a Ty4Cons based vector devoid of the signal for degradation of *Kl*.*URA3* were very similar to the fluorescence levels of cells bearing the single integrative vector (Fig 4). Higher fluorescence levels were only obtained when vector backbones combined Ty4Cons together with the expression of the fusion protein *Kl*.Ura3-degradation signal (Fig 4). In that case, fluorescence levels of the cell population are highly homogeneous with 91% of the cell population characterized by high expression levels, while equivalently high expression levels are observed for a smaller fraction of the cell population in the case of the episomal 2μ based (57%), the Ty4 based vector devoid of *Kl*.Ura3-degradation signal fusion (2%), and the single integrative vector (4%) (Fig 4). Furthermore, the cell population bearing the episomal 2μ vector is highly heterogeneous with regards to fluorescence levels as a large fraction of the cell population, i.e. 25% of the cell population, are expressing very low levels of fluorescence. This fraction becomes marginal in the case of the EasyCloneMulti vector. In conclusion, the vector backbone comprising both Ty4Cons for integration at Ty4 sequences and the *Kl*.*URA3* selective marker fused to the degradation signal was successful in triggering high expression levels of GFP and largely eliminated the fraction of cells expressing low levels of fluorescence observed in the case of the episomal vector.

### Multi-integration into different subsets of Ty sequences leads to different expression levels and copy numbers

**Fig 5:**
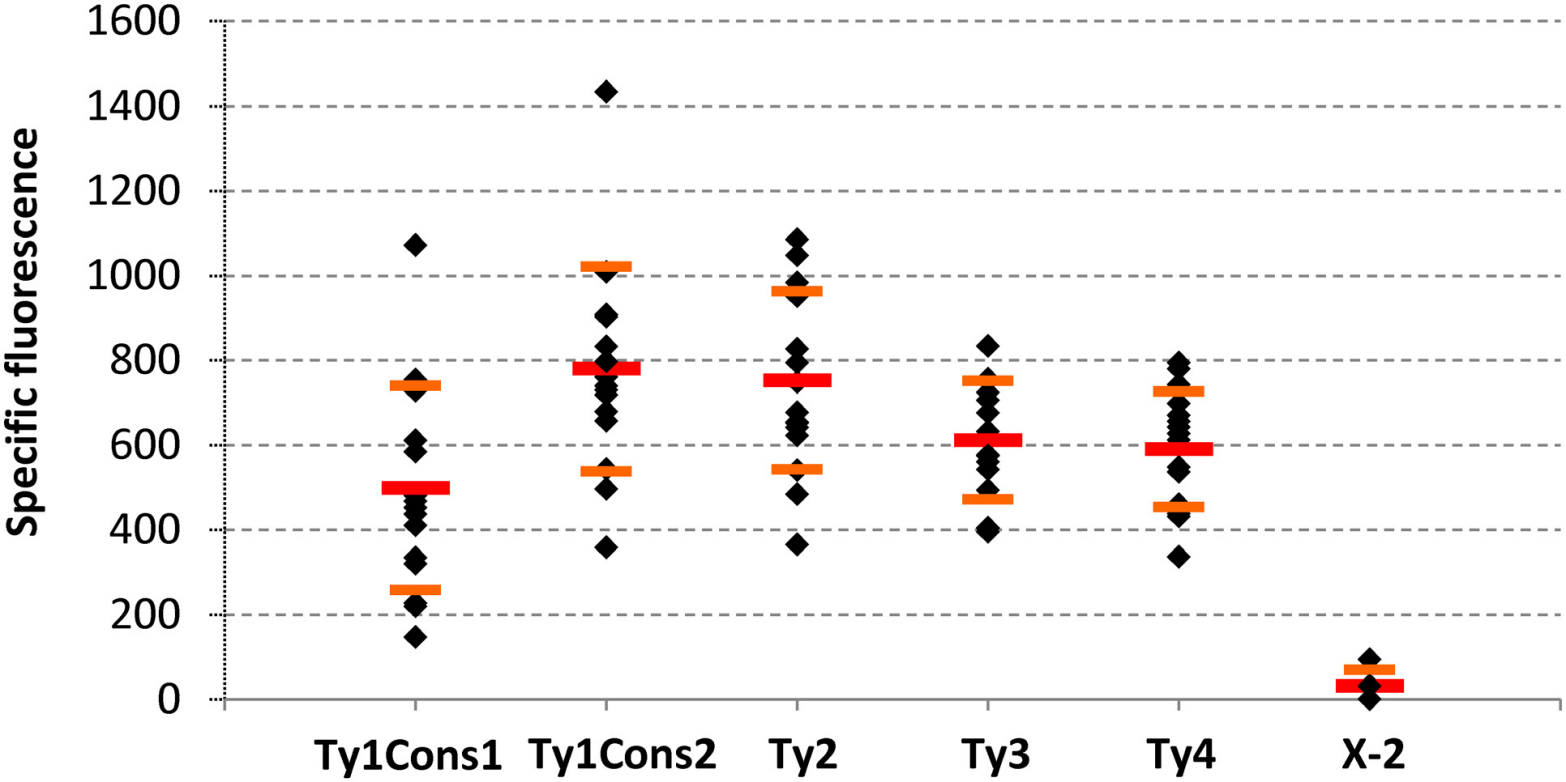
Effect of the different EasyCloneMulti vector backbones on fluorescence levels of a GFP reporter gene. Analysis of specific fluorescence levels of EasyCloneMulti vectors containing the reporter cassette P_*TEF1*_-GFP-T_*CYC1*_ and bearing the indicated consensus sequence for integration at Ty sequences: pCfB1136 (Ty1Cons1), pCfB1137 (Ty1Cons2), pCfB1138 (Ty2Cons), pCfB1139 (Ty3Cons), and pCfB326 (Ty4Cons). Integration of pCfB329 (single integrative, Chr. X-2) is used as reference for GFP expression from a single defined locus. The resulting fluorescence of *S*. *cerevisiae* CEN.PK.113-5D bearing either of the vectors was measured on a spectrophotometer. 16 individual clones for each vector type were tested. Average (red bars) and standard deviation (orange bar) are also represented.

The EasyCloneMulti vector set was expanded to include the respective Ty1Cons1, Ty1Cons2, Ty2, or Ty3 consensus sequences. All contained the fusion protein *Kl*.Ura3-degradation signal as selective marker. Each vector was linearized by *Not*I and transformed into *S*. *cerevisiae*. Resulting fluorescence levels are presented in Fig 5. All EasyCloneMulti vectors led to increased average fluorescence levels as compared to the single integrative vector that integrates at locus 2 on chromosome X (Fig 5). In general, the different EasyCloneMulti vectors are characterized by different ranges of fluorescence levels, which may reflect different numbers of insertions or differences in gene expression depending on the location of the insertion sites on the genome. Ty1Cons2 and Ty2 based EasyCloneMulti vectors sustain a highly similar range of fluorescence levels, which may reflect the high level of identity of Ty1Cons2 and Ty2 (Fig 2C). Average fluorescence levels for EasyCloneMulti vectors are 19 to 26 times higher than the fluorescence level of the single integrative vector, with extreme clones showing increased fluorescence levels up to about 50 times. Within the same vector type, the range of fluorescence level spans from 2-fold increase for the Ty3Cons based vector, to a 7-fold increase for the Ty1Cons1 based vector (Fig 5).

Interestingly, transformation efficiency for the EasyCloneMulti vectors was only reduced approximately 3-fold, with 1.5.10^3^ transformants.μg^-1^ digested plasmid DNA, compared to the transformation efficiency of the single integrative vector that integrates at locus 2 of chromosome X, with 5.10^3^ transformants.μg^-1^ digested plasmid DNA. It is however likely that the efficiency of transformation will drop when genes encoding enzymes of metabolic pathways are to be overexpressed. This is indeed what we observed in the case of the proof of concept study on 3HP production and report further below. We further expanded the EasyCloneMulti vector collection with a different selective marker: *Kl*.*LEU2*, fused to the CL-1 degradation signal as described above [35]. We observed the same pattern for these five new EasyCloneMulti vectors, which can therefore also conveniently be used for multicopy integration (S1 Fig). We also constructed EasyCloneMulti vectors with the *Sp*.*HIS5* marker fused to the CL-1 degradation signal. Interestingly, we observed a 10-fold higher transformation efficiency, i.e. a 10-fold increase in surviving colonies on drop-out media, for all five EasyCloneMulti vectors with the *Sp*.*HIS5* marker compared to the EasyCloneMulti vectors with the *Kl*.*URA3* and *Kl*.*LEU2* markers (data not shown). However, *Sp*.*HIS5* based vectors only led to weak GFP fluorescence levels, very close to fluorescence levels of the single integrative vector (S1 Fig). We therefore conclude that EasyCloneMulti vectors, based on *Kl*.*URA3* and *Kl*.*LEU2* markers fused to the CL-1 degradation signal, trigger simultaneous and multiple integrations into the *S*. *cerevisiae* genome. In turn, this leads to multiple gene copies and to a range of expression levels of the GFP reporter.

We then used next generation sequencing to analyze copy numbers and integration loci for five *S*. *cerevisiae* clones containing an EasyCloneMulti vector expressing GFP. From the experiment reported in Fig 5, one isolate from each type of EasyCloneMulti vector was analyzed by next generation sequencing (S2 Fig). One additional isolate expressing GFP from a single locus, locus X-2, was used as reference for GFP integrated at a single locus (Fig 5 and S2 Fig). As expected, the copy number was one for the isolate with single integration. The isolates with Ty sequence-targeted integrations had copy numbers ranging from ~30 to ~260 (Table 1). The specific fluorescence was found to depend linearly on the logarithm of the estimated copy number (S2 Fig). The copy numbers estimated for GFP and the heterologous *Kl*.*URA3* marker present on all EasyCloneMulti vectors were consistent with each other indicating that the copy number estimation is reliable and that intact vectors integrated in multiple copies (Table 1). We hereby confirmed by whole genome sequencing that EasyCloneMulti vectors trigger multiple integrations on the genome of *S*. *cerevisiae*.

**Table 1:** Copy numbers and number of integration loci estimated from next generation sequencing data.

EasyCloneMulti type	Ty1Cons1	Ty1Cons2	Ty2Cons	Ty3Cons	Ty4Cons	X-2
Clone ID	***clone B1***	***clone H4***	***clone D6***	***clone B8***	***clone D9***	***clone E11***
GFP copy number	44,1	265,2	58,0	32,4	28,9	1,1
*Kl*.*URA3* copy number	42,2	263,1	55,5	28,2	30,6	1,4
H.R. 5' copy number	148,9	382,0	87,2	46,4	12,4	1,1
H.R. 3' copy number	161,6	111,7	75,0	52,1	14,7	1,0
Specific fluorescence	611,5	796,8	483,7	575,0	643,1	30,6
Number of potential insertion loci	12	2	15	4	43	1
Number of high confidence insertion loci	1	0	6	0	12	1
Tandem duplication	No	Yes	No	No	No	No

We next determined potential insertion loci by examining cases where one paired read mapped to a unique sequence on the integrative vector and the other paired read mapped to a specific region on the chromosomes. This analysis confirmed the correct integration locus for the single copy X-2 integrated GFP and identified 1 to 12 high confidence integration sites for three of the Ty sequence based integrations (Table 1). However, for the other isolates we could not confidently determine any integration locus although some loci with a few mapped reads could be found. This is somewhat expected due to the high copy numbers observed for the isolates with Ty sequence based integrations, meaning that very few reads would map to any specific locus. For example if we sequence the genome at 100X coverage and a construct had a copy number of 50, we would expect to find on average two reads mapping at each integration locus. This number is too low to be detected with confidence from background non-specific mapping of reads. We have included all potential integration loci found, even with low confidence, as S5 Table. Nevertheless, the 6 and 12 high confidence insertion loci identified for the two isolates bearing Ty2Cons and Ty4Cons based vectors indicate that 5/6 and 7/12 insertions are located on different chromosomes, respectively (S5 Table). Furthermore, the distance between insertion loci on similar chromosomes is at least 5000bp (S5 Table). This indicates that, as intended, EasyCloneMulti vectors are able to target different loci for integration that are mostly located on different chromosomes.

We further investigated whether there was a potential for tandem duplication at a single locus. Tandem duplicates could be detected by manually inspecting read mappings to the vector sequences and identifying read pairs that mapped in a pattern indicative of a tandem duplication. Tandem duplications were also supported by observing differential copy numbers for the left and right integration regions on the vector (Table 1). Out of the five isolates sequenced, we observed one clear case of tandem duplication (Ty1-Cons2_H4), which may partially explain the unusually high GFP copy number obtained (Table 1). Tandem duplications are not surprising for integration at Ty sequences and have previously been reported in the literature [25,26]. In most of the studies, integrations occurred in a limited number of integration sites but in tandem arrays [26]. It is believed that the first event of integration of the Ty targeting vector may accelerate the next integration at the same site because more Ty sequences are available around the first target site, making the first integration region a hot spot for integration [26]. The design of the EasyCloneMulti vectors, which comprise two homologous regions for recombination of approximately 170bp of length, in direct orientation with respect to one another, flanking the expression cassette and the selective marker, may reduce the possibility of insertions in tandem and favor integrations at distinct loci.

Stability of expression from the different EasyCloneMulti vectors was challenged during a serial transfer cultivation experiment in synthetic drop out medium (S3 Fig). After 16 generations of growth, isolates from most of the EasyCloneMulti constructs tested retained more than 75% or their initial fluorescence level (S3B Fig). Ty2Cons based vectors proved the least stable with only 31% of the isolates retaining over 75% of their original fluorescence after 16 generations. This number further decreased to 19% after 32 generations (S3B Fig). After 32 generations, for most of the EasycloneMulti constructs tested, more than 50% of the isolates retained over 75% of their original fluorescence levels (S3B Fig). It is therefore possible to identify *S*. *cerevisiae* strains that carry multiple copies of EasyCloneMulti borne genes in their genomes and stably express them over generations. For this purpose, maintaining selective pressure for the vectors is a requirement, as decreased GFP expression levels were observed upon cultivation in non-selective media, i.e. YPD (data not shown). As EasyCloneMulti bearing strains are prototrophic, the use of minimal medium for cultivation is a cheap and convenient way to maintain selection for the vectors during growth.

### Balancing gene expression level in the 3HP pathway using EasyCloneMulti vectors

**Fig 6:**
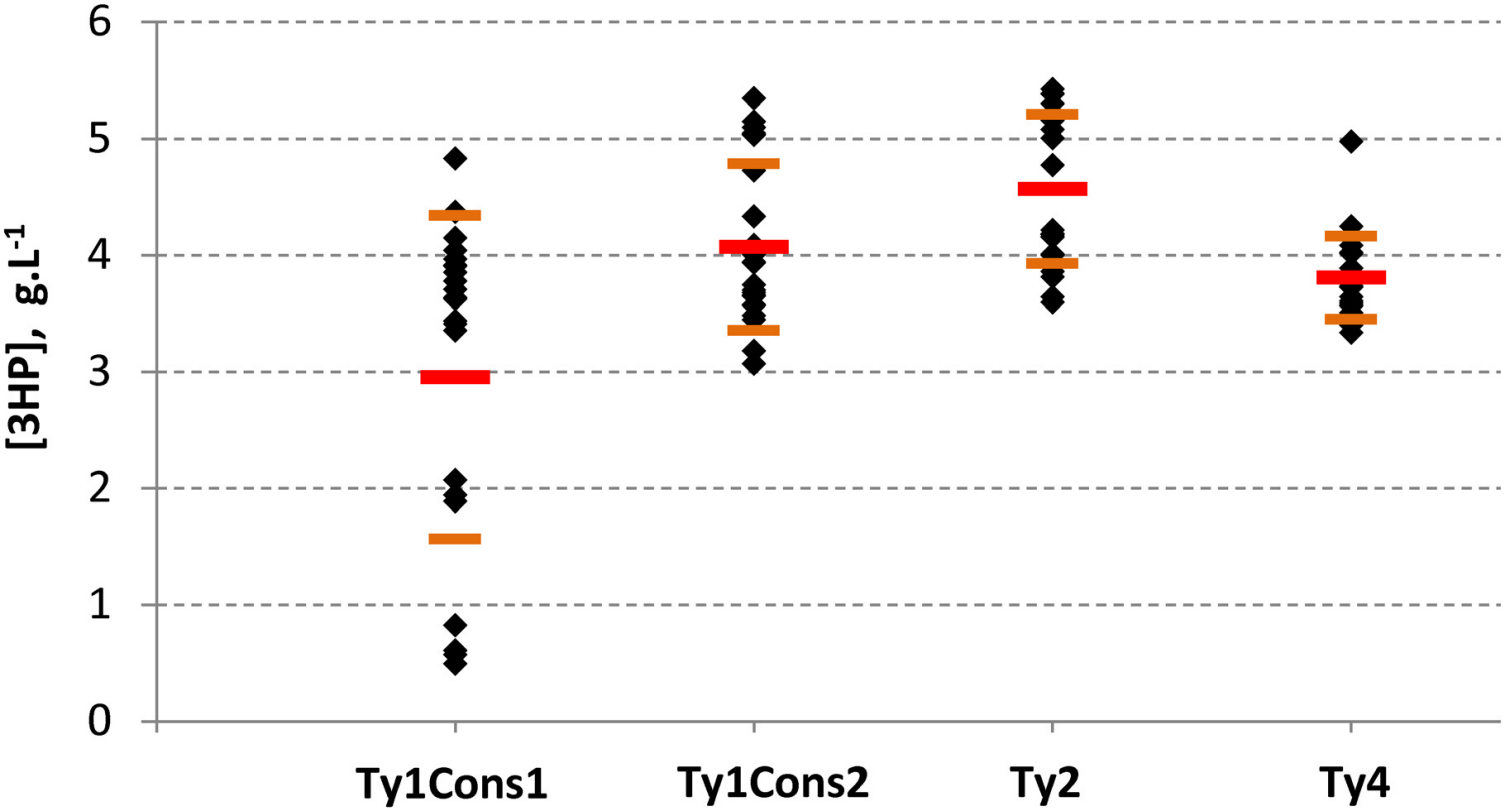
3HP production by *S*. *cerevisiae* strains bearing *T*. *castaneum panD* on different EasyCloneMulti vectors. Yeast strain SCE-iL1-155 was transformed with different EasyCloneMulti vectors that are expressing *T*. *castaneum panD*: pCfB2099 (Ty1Cons1), pCfB2097 (Ty1Cons2), pCfB2096 (Ty2Cons), or pCfB799 (Ty4cons). Final 3HP titers after cultivation in synthetic fed-batch medium (m2p-Labs) are shown. Average (red bars) and standard deviation (orange bar) are also represented.

3HP is a platform chemical, which can be converted into acrylic acid, 1,3-propanediol, malonic acid, and other valuable chemicals. In 2011, the world annual production of acrylic acid was 5000 kMT and the market size was USD 11.5 billion. Acrylic acid-derived products include superabsorbent polymers used in diapers and incontinence products, plastics, coatings, adhesives, elastomers, and paints [32]. Borodina *et al*. recently demonstrated a synthetic route for the biosynthesis of 3HP in *S*. *cerevisiae* via β–alanine [32]. They identified the enzymatic reaction catalyzed by PanD from the red flour beetle *Tribolium castaneum* as the main flux controlling step. Integration of *T*. *castaneum panD* in multiple copies, using a Ty4 based integrative vector, led to approximately 4-fold increase in 3HP production in their best producing strain [32]. Here we decided to investigate whether varying expression levels of *panD* by the EasyCloneMulti vector set could impact and potentially further improve 3HP production. The DNA fragment containing *panD* from *T*. *castaneum* under the control of the native *S*. *cerevisiae TEF1* promoter was cloned into EasyCloneMulti vectors for multiple insertions at different Ty sequences. The different types of EasyCloneMulti vectors bearing *panD* were transformed into a *S*. *cerevisiae* strain overexpressing three steps of the β–alanine pathway (SCE-iL1-155) and thereby completed the metabolic pathway to yield 3HP. A reduced transformation efficiency for EasyCloneMulti vectors bearing the *T*. *castaneum panD* expression cassette compared to a single integrative vector bearing the same gene was observed, with efficiencies of 10^1^ to 10^2^ transformants.μg^-1^ digested plasmid DNA and 4.10^3^ transformants.μg^-1^ digested plasmid DNA, respectively (data not shown). Despite this reduction in transformation efficiency when using the different EasyCloneMulti vectors, we were still able to further improve 3HP production, as the overall span of 3HP titers obtained for this experiment was approximately 11-fold (Fig 6). An approximate 20% increase in 3HP average titer was also observed when *panD* was expressed from Ty2Cons based vectors, as compared to Ty4Cons based ones. The highest producing clone based on a Ty2Cons EasyCloneMulti vector produced 40% more than the average 3HP titer of the Ty4Cons EasyCloneMulti vector (Fig 6). It should be further mentioned that no major impact on final biomass concentration was observed (S6 Table). We hereby demonstrate the usefulness of the EasyCloneMulti vectors to vary and increase expression levels of a metabolic enzymatic step.

## Discussion

We demonstrate in this work that the EasyCloneMulti vector set is an efficient and reliable strain construction tool to integrate heterologous genes in multiple copies into the genome of *S*. *cerevisiae*. Recently, the combinatorial assembly of biochemical pathways into yeast via constructs targeting one retrotransposon δ-site coupled with antibiotic selection has been reported [29,43]. In these studies, copy number was adjusted using different antibiotics concentrations. Another new system, the D-POP system, integrates genes in high copy in consecutive integration events [44]. Our EasyCloneMulti vector set is distinct from these studies, since EasyCloneMulti vectors are based on the EasyClone system, and thereby offer the convenient and efficient assembly of standardized DNA bricks into multi-loci targeting vectors via USER-cloning [16]. We show that EasyCloneMulti vectors result in simultaneous multiple integrations on the genome of *S*. *cerevisiae*, during one single yeast transformation step. Multiple integrations at distinct loci and on different chromosomes were confirmed by genome sequencing. This feature, as opposed to previous studies which mostly report about multiple integrations in tandem arrays [24–27], owes to the design of the EasyCloneMulti vectors. With the upstream and downstream regions for homologous recombination flanking expression cassettes and the selective marker, our vector system favors integrations via gene conversion events. In previous studies, integrations at Ty sequences in tandem are probably the consequence of the integration vectors being designed for integrations via single cross over mechanisms. We believe that integration at several and distinct loci, mediated by the EasyCloneMulti vectors, will prove beneficial for genomic stability as, in this case, integrated genes will most likely be separated by essential elements for growth which in turn may reduce chances for loop out events. Similar to our system, the most recent reports also rely on gene conversion events for integration at Ty sequences [28,29,43]. Additionally, EasyCloneMulti can readily be used with two different types of auxotrophic markers, *Kl*.*URA3* or *Kl*.*LEU2*, which makes it possible to study the effect of varying expression levels of up to four genes. Importantly, it was shown that high expression levels of GFP from EasyCloneMulti vectors can be maintained for 32 generations (S3 Fig), supporting a genomic stability suitable for the construction of production strains.

The two different selective markers, *Kl*.*URA3* and *Kl*.*LEU2* are fused to the CL-1 degradation signal. In *S*. *cerevisiae*, destabilizing or weakening the expression of a selective marker to increase the copy number of a plasmid has previously been reported [45,46]. It should be highlighted here that, in our study, the quick degradation of the selective marker caused by the CL-1 degradation signal is essential for the multi-integration phenotype. This may be explained by the fitness penalty such a feature provides. A quick degradation of Ura3 or Leu2 reduces the half-life of the protein and in turn imposes a positive selection for increased copy number of that gene in yeast cells. Both *Kl*.*URA3* and *Kl*.*LEU2* markers yield the same results with regards to GFP fluorescence levels, and can therefore conveniently be used for multicopy integration of genes. However, EasyCloneMulti vectors containing the *Sp*.*HIS5* marker fused to the CL-1 degradation signal only led to marginal increases of fluorescence levels compared to a single integrative vector (S1 Fig). This might reflect the low efficiency of the chosen degradation signal on His5 possibly due to protein structure that could potentially bury the C-terminal degradation tail. Also, high protein stability or long half-life of the His5 protein might be causative reasons for the low integration frequencies observed. It might also be that only trace amounts of the His5 protein are needed for survival on drop-out media. In any case, using this last set of vectors based on *Sp*.*HIS5* for multicopy integration is not recommended.

As a proof of concept, we used EasyCloneMulti vectors to integrate the rate-controlling enzyme PanD from *T*. *castaneum* into a strain that contains the β–alanine dependent metabolic pathway to produce 3HP [32]. We demonstrated the ability of the vector set to significantly increase 3HP titers. It has been established earlier that multiple copies of *panD* integrated using a Ty4Cons based EasyCloneMulti vector led to a higher production of 3HP as compared to single integration of *panD* [32]. Here we could further increase production by using an EasyCloneMulti vector targeting Ty2Cons sites. Interestingly, when compared to the high fold-increase of GFP fluorescence of EasyCloneMulti constructs, 3HP production was increased to a lesser extent, although still significantly higher than with single-copy *panD* (extrapolated from [32]). This might indicate that PanD ceases to be the flux-controlling step of the β-Alanine pathway at such high levels of expression. The number of integrated copies in the different isolates was not measured here but other studies from Borodina’s laboratory which used EasyCloneMulti types of vectors reported integrated copy numbers. Stovicek *et al*., for example, worked on laboratory and industrial strains and C5 sugars fermentation. They reported 6 to 8 integrated copies of two genes necessary for xylose consumption and borne on EasyCloneMulti type vectors targeting Ty2 loci [31]. The strains carrying this number of multiple copies were obtained after selection on increased antibiotic concentration and sustain growth rates in the order of 0.28 h^-1^ [31]. Li *et al*. integrated multiple copies of genes encoding enzymes of the resveratrol pathway using a Ty4 targeting EasyCloneMulti vector, they obtained 8 to 11 integrated copies [47]. Eventually, Kildegaard *et al*. in their study of the malonylCoA pathway for 3HP production, reported 3 to 4 integrated copies of two genes of that pathway using a Ty4 targeting EasyCloneMulti vector [Kildegaard et al, submitted].

Interestingly, depending on the Ty family targeted, different ranges of expression levels were achieved. This offers the unique possibility of easy and fast balancing of vector-borne gene expression by screening a small number of clones and identifying the best producer. This can be of great interest to metabolic engineers aiming at balancing expression levels of multi-step metabolic pathways. As was observed in our proof of concept study for 3HP production, transformation efficiency for the multi-integrative vectors was reduced compared to a single integrative one. This is somewhat expected due to the metabolic perturbation that may result from the overexpression of metabolic genes. In order to obtain a sizeable pool of transformants to screen from, increasing the amount of transformed linear DNA can be considered. Additionally, uncoupling transformation and integration events from expression of the borne gene of interest, by e.g. using inducible promoters, may improve transformation efficiency. One could then think of combining the EasyCloneMulti system with a set of user-defined promoters of different strengths, in a similar fashion to previous promoter studies [48,49], and generate combinatorial libraries of several genes with a broad range of expression levels. Associated to an easy screening method and combined with systems biology tools, this may contribute to understanding intrinsic control of multi-step metabolic pathways and constructing improved production strains.

As Ty sequences have been identified in a number of industrial yeast strains, it is likely that EasyCloneMulti vectors can be applied to industrial strain genetic engineering [50]. In fact, Stovicek *et al*. successfully integrated in the genome of the industrial strains Ethanol Red and CLIB382, and in multiple copies, two metabolic genes for xylose consumption using an EasyCloneMulti type of vector targeting Ty2 loci [31]. Furthermore, yeast production strains with genes integrated at Ty sequences have been reported for the industrial production of resveratrol [10], confirming the possibility of implementing our EasyCloneMulti vector set in industrial applications for biosustainable metabolite production. In the rare case where a specific yeast strain would be devoid of Ty sequences, other repeated DNA sequences such as ribosomal DNA sequences may be considered as targets for multiple integrations, as previously reported [30, 51].

In conclusion, EasyCloneMulti vectors complement the EasyClone and EasyClone 2.0 vector collections and provide a ready-to-clone and convenient solution for multiple integrations into *S*. *cerevisiae* genome. We believe that the EasyCloneMulti vector set is of high interest for balancing gene expression in metabolic pathways, and for industrial yeast metabolic engineering applications. Vectors will be deposited and be available at Addgene (www.addgene.org).

## Supporting Information

S1 Fig Specific fluorescence levels of *S*. *cerevisiae* strains transformed with EasyCloneMulti vectors based on *Kl*.*URA3*, *Kl*.*LEU2* and *Sp*.*HIS5* markers*. Specific fluorescence measurements as a function of the type of EasyCloneMulti vectors and of the type of selective marker used, *Kl*.URA3* (top), *Kl*.LEU2* (middle) and *Sp*.HIS5* (bottom) are shown. These three selective markers were ordered as synthetic DNA from GeneArt (LifeTechnologies). Fluorescence levels were compared to a reference strain bearing a single integration of GFP reporter cassette at locus X-2 [11]. Top: CEN.PK 113-5D transformed with one of the following vectors: pCfB2795 (multi-integrative, Ty1Cons1, *Kl*.URA3*-deg), pCfB2794 (multi-integrative, Ty1Cons2, *Kl*.URA3*-deg), pCfB2793 (multi-integrative, Ty2, *Kl*.URA3*-deg), pCfB2792 (multi-integrative, Ty3, *Kl*.URA3*-deg), pCfB2791 (multi-integrative, Ty4, *Kl*.URA3*-deg), and pCfB329 (single integrative at Chr. X-2, *Kl*.*URA3*). Middle: CEN.PK 113-32D transformed with one of the following vectors: pCfB2802 (multi-integrative, Ty1Cons1, *Kl*.LEU2*-deg), pCfB2801 (multi-integrative, Ty1Cons2, *Kl*.LEU2*-deg), pCfB2800 (multi-integrative, Ty2, *Kl*.LEU2*-deg), pCfB2799 (multi-integrative, Ty3, *Kl*.LEU2*-deg), and pCfB2798 (multi-integrative, Ty4, *Kl*.LEU2*-deg). CEN.PK 113-5D transformed with pCfB329 (single integrative at Chr. X-2, *Kl*.*URA3*) was used as a control for single integration. Bottom: CEN.PK 113-11A transformed with one of the following vectors: pCfB2809 (multi-integrative, Ty1Cons1, *Sp*.HIS5*-deg), pCfB2808 (multi-integrative, Ty1Cons2, *Sp*.HIS5*-deg), pCfB2807 (multi-integrative, Ty2, *Sp*.HIS5*-deg), pCfB2806 (multi-integrative, Ty3, *Sp*.HIS5*-deg), and pCfB2805 (multi-integrative, Ty4, *Sp*.HIS5*-deg). CEN.PK 113-5D transformed with pCfB329 (single integrative at Chr. X-2, *Kl*.*URA3*) was used as a control for single integration based fluorescence levels. Average (red bars) and standard deviation (orange bar) are also represented. * *Kl*.*URA3*, *Kl*.*LEU2* and *Sp*.*HIS5* were ordered as synthetic DNA from GeneArt (LifeTechnologies).
(DOCX)

S2 Fig Specific fluorescence and estimated copy number of the GFP gene based on Illumina sequencing data. Top: Specific fluorescence as a function of the EasyCloneMulti vector backbones. This figure is adapted from Fig 5, where clones analysed for whole genome sequencing are marked in yellow. Average (red bars) and standard deviation (orange bar) are presented. Bottom: Specific fluorescence measured for each of the clones analysed plotted against the estimated copy number of the GFP gene based on Illumina sequencing data.
(DOCX)

S3 Fig Evolution of specific green fluorescence during a serial transfer experiment. For this experiment, six different strains obtained after transformation of CEN.PK 113-5D by one of the following vectors are considered: pCfB2795 (multi-integrative, Ty1Cons1, *Kl*.URA3*-deg), pCfB2794 (multi-integrative, Ty1Cons2, *Kl*.URA3*-deg), pCfB2793 (multi-integrative, Ty2, *Kl*.URA3*-deg), pCfB2792 (multi-integrative, Ty3, *Kl*.URA3*-deg), pCfB2791 (multi-integrative, Ty4, *Kl*.URA3*-deg), or pCfB319 (episomal, 2micron, *URA3*). A) Sixteen isolates from each of the six strains were inoculated from solid drop out medium without uracil (transformation plate) into liquid drop out medium without uracil. The experiment was then realized in 96 deep well-plates, with 800 μL as cultivation volume. After 24h of cultivation, OD_600_ was measured and a new 96 deep-well-plate containing fresh liquid drop out medium without uracil was inoculated at an initial OD_600_ of 0.05. After 48h of cultivation, OD_600_ and green fluorescence (λ_Excitation_ 485nm, λ_Emission_ 515nm) were measured for each cultivation plate on a microtiter plate reader BioTek Synergy MX (BioTek). Results are reported as specific fluorescence which is obtained by dividing the measured fluorescence by the measured OD_600_. OD_600_ and specific fluorescence were followed for four subsequent cultivations. B) In this table, the specific fluorescence relative to the initial specific fluorescence measured in the first cultivation, cultivation 1, is compared for the sixteen isolates of each strain at two time points: 16 generations (cultivation 2) or 32 generations (cultivation 4). Percent of isolates characterized by a specific fluorescence above 75%, between 50 and 75% or below 50% of the initial specific fluorescence measured in cultivation 1 is reported for the abovementioned time points, as a function of the type of EasyCloneMulti vector.
(DOCX)

S1 File Supplementary methods and vector sequences. (DOCX)

S2 File Supplementary references. (DOCX)

S1 Table List of the consensus sequences. (DOCX)

S2 Table List of strains used in this study. (DOCX)

S3 Table Primer sequences. Sequences of primers (5´ to 3´) used in this study. Overhangs used for USER cloning are underlined.
(DOCX)

S4 Table List of plasmids used in this study. (DOCX)

S5 Table Putative insertion loci identified from isolate sequencing. Candidate insertion loci were identified by finding reads mapped to a specific chromosomal location whose paired end partner read mapped to a unique region (i.e. a region with no similarity to the yeast genome) on the vector insert. The table indicates all loci identified using this approach, but the candidate loci that either have narrow mapped read stacks or low coverage are not likely to be real insertion loci. The percentage of mapped reads indicates what percentage of reads identified as mapping to candidate insertion loci map to the particular location specified in the table.
(DOCX)

S6 Table Final biomass concentrations of *S*. *cerevisiae* strains bearing *T*. *castaneum panD* on different EasyCloneMulti vectors. (DOCX)
