# Supplementary material for: EasyCloneMulti: A Set of Vectors for Simultaneous and Multiple Genomic Integrations in Saccharomyces cerevisiae
Source: PLoS One. 2016 Mar 2;11(3):e0150394. doi: 10.1371/journal.pone.0150394 (PMC4775045; doi:10.1371/journal.pone.0150394)

**Supplementary Figure S1: Specific fluorescence levels of *S. cerevisiae* strains transformed with EasyCloneMulti vectors based on *Kl.URA3*, *Kl.LEU2* and *Sp.HIS5* markers*.**

Specific fluorescence measurements as a function of the type of EasyCloneMulti vectors and of the type of selective marker used, *Kl.URA3** (top), *Kl.LEU2** (middle) and *Sp.HIS5** (bottom) are shown. These three selective markers were ordered as synthetic DNA from GeneArt® (LifeTechnologies). Fluorescence levels were compared to a reference strain bearing a single integration of GFP reporter cassette at locus X-2 [4]. Top: CEN.PK 113-5D transformed with one of the following vectors: pCfB2795 (multi-integrative, Ty1Cons1, *Kl.URA3*-deg*), pCfB2794 (multi-integrative, Ty1Cons2, *Kl.URA3*-deg*), pCfB2793 (multi-integrative, Ty2, *Kl.URA3*-deg*), pCfB2792 (multi-integrative, Ty3, *Kl.URA3*-deg*), pCfB2791 (multi-integrative, Ty4, *Kl.URA3*-deg*), and pCfB329 (single integrative at Chr. X-2, *Kl.URA3*). Middle: CEN.PK 113-32D transformed with one of the following vectors: pCfB2802 (multi-integrative, Ty1Cons1, *Kl.LEU2*-deg*), pCfB2801 (multi-integrative, Ty1Cons2, *Kl.LEU2*-deg*), pCfB2800 (multi-integrative, Ty2, *Kl.LEU2*-deg*), pCfB2799 (multi-integrative, Ty3, *Kl.LEU2*-deg*), and pCfB2798 (multi-integrative, Ty4, *Kl.LEU2*-deg*). CEN.PK 113-5D transformed with pCfB329 (single integrative at Chr. X-2, *Kl.URA3*) was used as a control for single integration. Bottom: CEN.PK 113-11A transformed with one of the following vectors: pCfB2809 (multi-integrative, Ty1Cons1, *Sp.HIS5*-deg*), pCfB2808 (multi-integrative, Ty1Cons2, *Sp.HIS5*-deg*), pCfB2807 (multi-integrative, Ty2, *Sp.HIS5*-deg*), pCfB2806 (multi-integrative, Ty3, *Sp.HIS5*-deg*), and pCfB2805 (multi-integrative, Ty4, *Sp.HIS5*-deg*). CEN.PK 113-5D transformed with pCfB329 (single integrative at Chr. X-2, *Kl.URA3*) was used as a control for single integration based fluorescence levels. Average (red bars) and standard deviation (orange bar) are also represented.

* *Kl.URA3*, *Kl.LEU2* and *Sp.HIS5* were ordered as synthetic DNA from GeneArt® (LifeTechnologies).


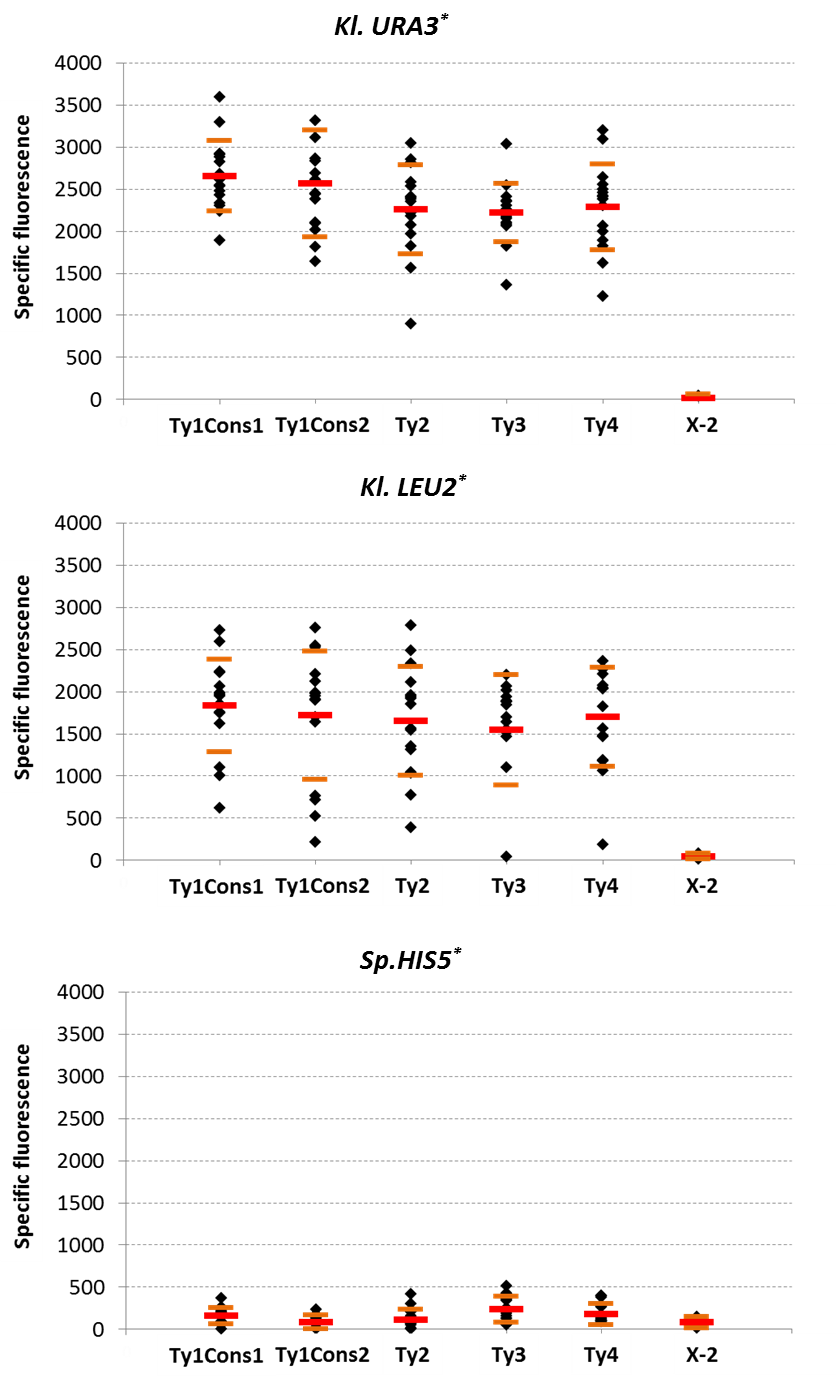

Supplement: S1 Fig — Specific fluorescence measurements as a function of the type of EasyCloneMulti vectors and of the type of selective marker used, Kl.URA3* (top), Kl.LEU2* (middle) and Sp.HIS5* (bottom) are shown. These three selective markers were ordered as synthetic DNA from GeneArt (LifeTechnologies). Fluorescence levels were compared to a reference strain bearing a single integration of GFP reporter cassette at locus X-2 [11]. Top: CEN.PK 113-5D transformed with one of the following vectors: pCfB2795 (multi-integrative, Ty1Cons1, Kl.URA3*-deg), pCfB2794 (multi-integrative, Ty1Cons2, Kl.URA3*-deg), pCfB2793 (multi-integrative, Ty2, Kl.URA3*-deg), pCfB2792 (multi-integrative, Ty3, Kl.URA3*-deg), pCfB2791 (multi-integrative, Ty4, Kl.URA3*-deg), and pCfB329 (single integrative at Chr. X-2, Kl.URA3). Middle: CEN.PK 113-32D transformed with one of the following vectors: pCfB2802 (multi-integrative, Ty1Cons1, Kl.LEU2*-deg), pCfB2801 (multi-integrative, Ty1Cons2, Kl.LEU2*-deg), pCfB2800 (multi-integrative, Ty2, Kl.LEU2*-deg), pCfB2799 (multi-integrative, Ty3, Kl.LEU2*-deg), and pCfB2798 (multi-integrative, Ty4, Kl.LEU2*-deg). CEN.PK 113-5D transformed with pCfB329 (single integrative at Chr. X-2, Kl.URA3) was used as a control for single integration. Bottom: CEN.PK 113-11A transformed with one of the following vectors: pCfB2809 (multi-integrative, Ty1Cons1, Sp.HIS5*-deg), pCfB2808 (multi-integrative, Ty1Cons2, Sp.HIS5*-deg), pCfB2807 (multi-integrative, Ty2, Sp.HIS5*-deg), pCfB2806 (multi-integrative, Ty3, Sp.HIS5*-deg), and pCfB2805 (multi-integrative, Ty4, Sp.HIS5*-deg). CEN.PK 113-5D transformed with pCfB329 (single integrative at Chr. X-2, Kl.URA3) was used as a control for single integration based fluorescence levels. Average (red bars) and standard deviation (orange bar) are also represented. * Kl.URA3, Kl.LEU2 and Sp.HIS5 were ordered as synthetic DNA from GeneArt (LifeTechnologies). (DOCX) [file pone.0150394.s001.docx]
