# Supplementary material for: EasyCloneMulti: A Set of Vectors for Simultaneous and Multiple Genomic Integrations in Saccharomyces cerevisiae
Source: PLoS One. 2016 Mar 2;11(3):e0150394. doi: 10.1371/journal.pone.0150394 (PMC4775045; doi:10.1371/journal.pone.0150394)
Supplement: S2 Fig — Top: Specific fluorescence as a function of the EasyCloneMulti vector backbones. This figure is adapted from Fig 5, where clones analysed for whole genome sequencing are marked in yellow. Average (red bars) and standard deviation (orange bar) are presented. Bottom: Specific fluorescence measured for each of the clones analysed plotted against the estimated copy number of the GFP gene based on Illumina sequencing data. (DOCX) [file pone.0150394.s002.docx]

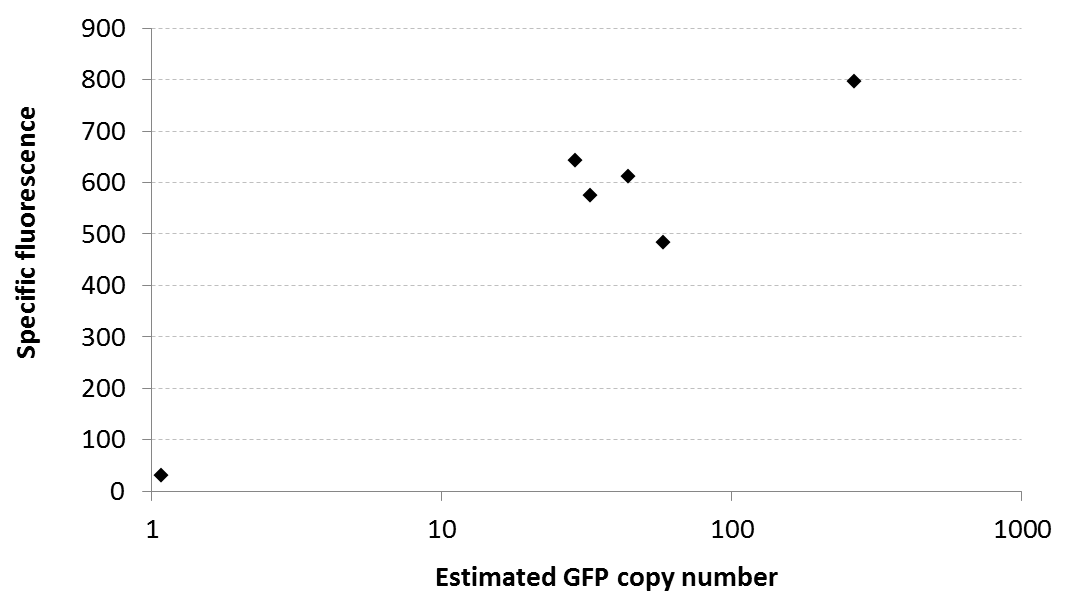

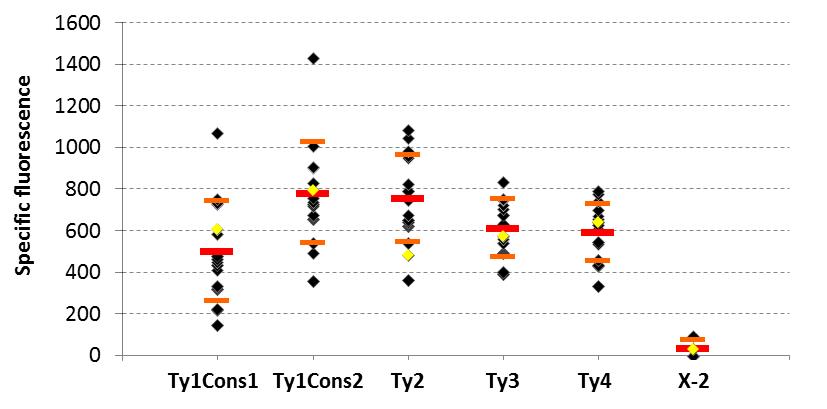
**Supplementary figure S2: Specific fluorescence and estimated copy number of the GFP gene based on Illumina sequencing data.** Top: Specific fluorescence as a function of the EasyCloneMulti vector backbones. This figure is adapted from figure 5, where clones analysed for whole genome sequencing are marked in yellow. Average (red bars) and standard deviation (orange bar) are presented. Bottom: Specific fluorescence measured for each of the clones analysed plotted against the estimated copy number of the GFP gene based on Illumina sequencing data.
