# Supplementary material for: EasyCloneMulti: A Set of Vectors for Simultaneous and Multiple Genomic Integrations in Saccharomyces cerevisiae
Source: PLoS One. 2016 Mar 2;11(3):e0150394. doi: 10.1371/journal.pone.0150394 (PMC4775045; doi:10.1371/journal.pone.0150394)
Supplement: S3 Fig — For this experiment, six different strains obtained after transformation of CEN.PK 113-5D by one of the following vectors are considered: pCfB2795 (multi-integrative, Ty1Cons1, Kl.URA3*-deg), pCfB2794 (multi-integrative, Ty1Cons2, Kl.URA3*-deg), pCfB2793 (multi-integrative, Ty2, Kl.URA3*-deg), pCfB2792 (multi-integrative, Ty3, Kl.URA3*-deg), pCfB2791 (multi-integrative, Ty4, Kl.URA3*-deg), or pCfB319 (episomal, 2micron, URA3). A) Sixteen isolates from each of the six strains were inoculated from solid drop out medium without uracil (transformation plate) into liquid drop out medium without uracil. The experiment was then realized in 96 deep well-plates, with 800 μL as cultivation volume. After 24h of cultivation, OD600 was measured and a new 96 deep-well-plate containing fresh liquid drop out medium without uracil was inoculated at an initial OD600 of 0.05. After 48h of cultivation, OD600 and green fluorescence (λExcitation 485nm, λEmission 515nm) were measured for each cultivation plate on a microtiter plate reader BioTek Synergy MX (BioTek). Results are reported as specific fluorescence which is obtained by dividing the measured fluorescence by the measured OD600. OD600 and specific fluorescence were followed for four subsequent cultivations. B) In this table, the specific fluorescence relative to the initial specific fluorescence measured in the first cultivation, cultivation 1, is compared for the sixteen isolates of each strain at two time points: 16 generations (cultivation 2) or 32 generations (cultivation 4). Percent of isolates characterized by a specific fluorescence above 75%, between 50 and 75% or below 50% of the initial specific fluorescence measured in cultivation 1 is reported for the abovementioned time points, as a function of the type of EasyCloneMulti vector. (DOCX) [file pone.0150394.s003.docx]

## Supplementary Figure S3: Evolution of specific green fluorescence during a serial transfer experiment.

For this experiment, six different strains obtained after transformation of CEN.PK 113-5D by one of the following vectors are considered: pCfB2795 (multi-integrative, Ty1Cons1, Kl.URA3*-deg), pCfB2794 (multi-integrative, Ty1Cons2, Kl.URA3*-deg), pCfB2793 (multi-integrative, Ty2, Kl.URA3*-deg), pCfB2792 (multi-integrative, Ty3, Kl.URA3*-deg), pCfB2791 (multi-integrative, Ty4, Kl.URA3*-deg), or pCfB319 (episomal, 2micron, URA3). **A)** Sixteen isolates from each of the six strains were inoculated from solid drop out medium without uracil (transformation plate) into liquid drop out medium without uracil. The experiment was then realized in 96 deep well-plates, with 800 μL as cultivation volume. After 24h of cultivation, OD_600_ was measured and a new 96 deep-well-plate containing fresh liquid drop out medium without uracil was inoculated at an initial OD_600_ of 0.05. After 48h of cultivation, OD_600_ and green fluorescence (λ_Excitation_ 485nm, λ_Emission_ 515nm) were measured for each cultivation plate on a microtiter plate reader BioTek Synergy MX (BioTek). Results are reported as specific fluorescence which is obtained by dividing the measured fluorescence by the measured OD_600_. OD_600_ and specific fluorescence were followed for four subsequent cultivations. **B)** In this table, the specific fluorescence relative to the initial specific fluorescence measured in the first cultivation, cultivation 1, is compared for the sixteen isolates of each strain at two time points: 16 generations (cultivation 2) or 32 generations (cultivation 4). Percent of isolates characterized by a specific fluorescence above 75%, between 50 and 75% or below 50% of the initial specific fluorescence measured in cultivation 1 is reported for the abovementioned time points, as a function of the type of EasyCloneMulti vector.


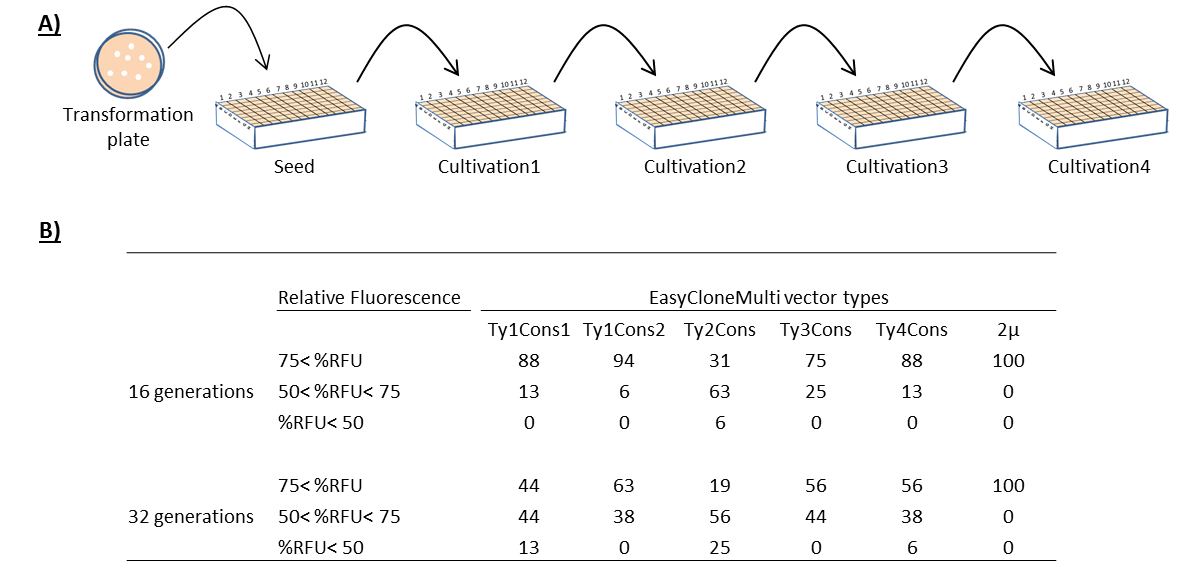


* *Kl.URA3*, was ordered as synthetic DNA from GeneArt® (LifeTechnologies).
