# Supplementary material for: EasyCloneMulti: A Set of Vectors for Simultaneous and Multiple Genomic Integrations in Saccharomyces cerevisiae
Source: PLoS One. 2016 Mar 2;11(3):e0150394. doi: 10.1371/journal.pone.0150394 (PMC4775045; doi:10.1371/journal.pone.0150394)
Supplement: S1 File — (DOCX) [file pone.0150394.s004.docx]

## Supplementary Methods

### Construction of EasyCloneMulti vectors with synthetic markers (*Kl.URA3*, Kl.LEU2*, Sp.HIS5**) and USER cassettes.

The gene fragments with overhangs for USER-cloning were generated by PCR amplification, USER-cloning reactions and *E. coli* transformations mainly performed on the Hamilton Vantage Cloning Robot. The PCR mix contained: 18 µl water, 10 µl HF phusion buffer (5x, BioLab), 5 µl 2mM dNTP, 2 µl PfuX7 polymerase [5], 2.5 µl forward primer (10 µM), 2.5 µl reverse primer (10 µM), and 1 µl DNA template. The cycling program was: 95°C for 2 min, 30 cycles of [95°C for 10 sec, 52°C for 20 sec, 68°C for (1min/kb)], 68°C for 5 min, pause at 10°C. The gene fragments were resolved on 1% agarose gel containing SYBR®-SAFE (Invitrogen) and purified using NucleoSpin® Gel and PCR Clean-up kit (Macherey-Nagel). The EasyCloneMulti plasmids were created by USER-cloning as described previously [2]. The plasmids of 4 clones/transformation were isolated from overnight *E. coli* cultures and confirmed by sequencing (Eurofins). The used primers are listed in Supplementary Table S3, and plasmids are listed in Supplementary Table S4.

For exchanging the *Kl.URA3* selection marker with *Kl.URA3** and *Kl.LEU2** synthetic DNA sequences from GeneArt® (LifeTechnologies), vectors pCfB326, pCfB1139, pCfB1138, pCfB1137, pCfB1136, pCfB322 and pCfB2047 were opened by PCR using the primer set PR-401/PR-9685. The synthetic markers *Kl.URA3** and *Kl.LEU2** were generated using primers PR-399/PR-9691 and PR-399/PR-9688 with pCfB2051 and pCfB2052 as a template, respectively. After uracil-excision reaction and transformation of chemically competent *E. coli* DH5α, the following vectors were obtained: pCfB2791, pCfB2792, pCfB2793, pCfB2794, pCfB2795, pCfB2796, pCfB2797, pCfB2798, pCfB2799, pCfB2800, pCfB2801, pCfB2802, pCfB2803, and pCfB2804. The features of the different vectors are summarized in Supplementary Table S4.

For exchanging the *Kl.URA3* selection marker with *Sp.HIS5** synthetic DNA from GeneArt® (LifeTechnologies), vectors pCfB326, pCfB1139, pCfB1138, pCfB1137, pCfB1136, pCfB322 and pCfB2047 were opened by PCR using the primer set PR-9689/PR-9690. The synthetic marker *Sp.HIS5** was generated using primers PR-9686/PR-9687 with pCfB2053 as a template. After uracil-excision reaction and transformation of chemically competent *E. coli* DH5α, the following vectors were obtained: pCfB2805, pCfB2806, pCfB2807, pCfB2808, pCfB2809, pCfB2810, and pCfB2811. The features of the different vectors are summarized in Supplementary Table S4.

For exchanging the GFP expression cassette with the USER cassette, vectors pCfB1137, pCfB2792, pCfB2794, pCfB2795, pCfB2799, pCfB2801, and pCfB2802 were opened by PCR using the primer set PR-2849/PR-2851. The USER cassette insert was generated using primers PR-2848/PR-2850 with pCfB257 as a template. After uracil-excision reaction and transformation of chemically competent *E. coli* DH5α, the following vectors were obtained: pCfB2046, pCfB2875, pCfB2988, pCfB2989, pCfB2990, pCfB2991, and pCfB2992, respectively. The features of the different vectors are summarized in Supplementary Table S4.

### Construction of EasyCloneMulti vectors for *Tc.panD* overexpression.

The gene encoding the *T. castaneum* L-aspartate-α-carboxylase *panD* used in this study has been published previously in [1], and had been synthesized by GeneArt (Life Technologies) in a codon-optimized version for yeast *S. cerevisiae* (GenBank: [ABU25221](http://www.ncbi.nlm.nih.gov/protein/ABU25221)). The synthetic gene construct had the structure: *AAAACA***ATG**NN…NN**TGA**, where AAAACA is the Kozak sequence, ATG is the start codon, NN…NN represents the protein coding sequence without start and stop codons, TGA is the stop codon.

For cloning of plasmids pCfB2097 and pCfB2096, the gene brick carrying the gene and correct overhangs for USER-cloning was generated by PCR using primers PR-652/PR-653 with the synthetic DNA as the template followed by DNA purification. The promoter fragment was also generated by PCR using primers PR-5/PR-6 with genomic *S. cerevisiae* DNA as a template, and followed by DNA purification. The terminators were already present on the EasyCloneMulti vectors. The expression plasmids were created by USER-cloning into the EasyCloneMulti vectors pCfB2046 and pCfB2047 as described previously [2], and the following vectors were obtained: pCfB2097 and pCfB2096, respectively.

For plasmid pCfB2099, we exchanged the GFP expression cassette with the *P_TEF1_*->*Tc.panD* expression cassette. Vector pCfB1136 was opened by PCR using the primer set PR-2849/PR-2851. The *P_TEF1_*->*Tc.panD* expression cassette insert was generated using primers PR-2848/PR-2850 with pCfB799 as a template. We then performed uracil-excision reaction and transformation of chemically competent *E. coli* DH5α to obtain pCfB2099.

**Vector sequences**

**pCfB2989_(pTY1Cons1-KlURA3-deg) 5472 bp DNA circular UNA**

FEATURES Location/Qualifiers

Misc._feature 1..14

/label="USER cassette"

Terminator 20..190

/label="T CYC1"

Misc._feature 257..316

/label=loxP

Terminator complement(317..439)

/label="T URA3"

Misc._structure complement(440..490)

/label="Degradation tag"

CDS complement(491..1291)

/label="K. lactis URA3syn without STOP codon"

/product="orotidine-5'-phosphate decarboxylase"

/protein_id="AAG34531.1"

/db_xref="GI:11344893"

Promoter complement(1292..1790)

/label="K. lactis URA3 promoter"

Misc._feature 1807..1840

/label=loxP

Misc._feature 1980..2187

/label="TY1 C1 3'"

/label="Consensus TY1Cons1 3'"

Replication_ori complement(2960..3603)

/label="pUC ori"

CDS complement(3706..4564)

/label=AmpR

Misc._feature 5008..5241

/label="TY1 C1 5'"

/label="Consensus TY1Cons1 5'"

Terminator complement(5268..5462)

/label="T ADH1"

ORIGIN

1 CGTGCGATCG CGTGCATTCA TCCGCTCTAA CCGAAAAGGA AGGAGTTAGA CAACCTGAAG

61 TCTAGGTCCC TATTTATTTT TTTATAGTTA TGTTAGTATT AAGAACGTTA TTTATATTTC

121 AAATTTTTCT TTTTTTTCTG TACAGACGCG TGTACGCATG TAACATTATA CTGAAAACCT

181 TGCTTGAGAA GGTTTTGGGA CGCTCGAAGA TCGCGTCAGC TGAAGCTTCG TACGCTGCAG

241 GTCGACAACC CTTAATGTCG ACAACCCTTA ATATAACTTC GTATAATGTA TGCTATACGA

301 AGTTATTAGG TCTAGAGATC CCAATACAAC AGATCACGTG ATCTTTTGTA AGATGAAGTT

361 GAAGTGAGTG TTGCACCGTG CCAATGCAGG TGGCTATTAG ATTAAATATG TGATTTGTTC

421 TATTAAGTTT CCTGTATAAT TACAAATGAA TAACGAAATG AGACAAAGAA GAGAACCAAU

481 TTTTACAAGC ATGGGGAGCG CTGATTCTCT TTTGGTACGC TTCCCATCCA GCATTTCTGT

541 ATCTTTCACC TTCAACCTTA GGATCTCTAC CCTTGGCGAA AAGTCCTCTG CCAACAATGA

601 TGATATCTGA TCCACCACTT ACAACTTCGT CGACGGTTCT GTACTGCTGA CCCAATGCAT

661 CGCCTTTGTC GTCTAAACCT ACACCTGGGG TCATGATTAG CCAATCAAAC CCTTCTTCTC

721 TTCCTCCCAT ATCGTTCTGA GCAATGAACC CAATAACGAA ATCTTTATCA CTCTTTGCAA

781 TATCAACGGT ACCCTTAGTA TATTCACCGT GTGCTAGAGA ACCCTTGGAA GATAATTCAG

841 CAAGCATCAA TAATCCCCTT GGTTCTTTGG TGACCTCTTG CGCACCTTGT TTCAAGCCAG

901 CAACAATACC AGCACCAGTA ACCCCGTGGG CGTTGGTGAT ATCAGACCAT TCTGCGATAC

961 GGTAAACGCC CGATGTATAT TGTAATTTGA CTGTGTTACC GATATCGGCG AATTTTCTGT

1021 CCTCAAATAT CAAGAACTTG TATTTCTCTG CCAATGCTTT CAATGGAACG ACAGTACCCT

1081 CATAACTGAA ATCATCCAAG ATATCAACGT GTGTTTTCAA AAGGCAAATG TATGGACCCA

1141 ACGTTTCAAC AAGTTTCAAT AGCTCATCAG TCGAACGAAC GTCAAGAGAA GCACACAAAT

1201 TGGTTTTCTT TTCATCCATT AAACGTAAAA GTTTCGATGC AACCGGACTT GCATGAGTCT

1261 CAGCTCTACT GGTATATGAT TTTGTGGACA TGGTGCAACT AATTGACGGG AGTGTATTGA

1321 CGCTGGCGTA CTGGCTTTCA CAAAATGGCC CAATCACAAC CACATCTTAG ATAGTTGAAA

1381 TGACTTTAGA TAACATCAAT TGAGATGAGC TTAATCATGT CAAAGCTAAA AGTGTCACCA

1441 TGAACGACAA TTCTTAAGCA AATCACGTGA TATAGATCCA CGAATAACCA CCATTTGATG

1501 CTCGAGGCAA GTAATGTGTG TAAAAAAATG CGTTACCACC ATCCAATGCA GACCGATCTT

1561 CTACCCAGAA TCACATATAT TTATGTACCG AGTACCTTTT TTCTATCTTC CAATTGCTTC

1621 TCCCATATGA TTGTCTCCGT AAGCTCGAAA TTTCTAAGTT GGATTTTAAT CTTCACGCAG

1681 GATGACAGTT CGATGAGCTT CTGAGGAGTG TTTAGAACAT AATCAGTTTA TCCATGGTCT

1741 ATCTCTTCTT GTCGCTTTTT CTCCTCGATA GAACCTAAAT AAAACGAGCT CTCGAGAACC

1801 CTTAATATAA CTTCGTATAA TGTATGCTAT ACGAAGTTAT TAGGTGATAT CAGATCCACT

1861 AGTGGCCTAT GCACCCAATT CGCCCTATAG TGAGTCGTAT TACGCGCGCT CACTGGCCGT

1921 CGTTTTACAA CGTCGTGACT GGGAAAACCC TGGCGTTACC CCTGCAGGAC AGACGTCATT

1981 AGTGCTGAGG CATTAATTGA TCAAAAACGG AATGAGGAAT AATCGTAATA TTAGTATGTA

2041 GAAATATAGA TTCCATTTTG AGGATTCCTA TATCCTCGAG GAGAACTTCT AGTGTATATT

2101 CTGTATACCT AATATTATAG CCTTTATCAA CAATGGAATC CCAACAATTA TCTAATTACC

2161 CACAAATTTC TCAAGATCTG CGGCCGCACT CAGACCTGAA GTGAAGTTCC TATACTTTCT

2221 AGAGAATAGG AACTTCTATA GTGAGTCGAA TAAGGGCGAC ACAAAATTTA TTCTAAATGC

2281 ATAATAAATA CTGATAACAT CTTATAGTTT GTATTATATT TTGTATTATC GTTGACATGT

2341 ATAATTTTGA TATCAAAAAC TGATTTTCCC TTTATTATTT TCGAGATTTA TTTTCTTAAT

2401 TCTCTTTAAC AAACTAGAAA TATTGTATAT ACAAAAAATC ATAAATAATA GATGAATAGT

2461 TTAATTATAG GTGTTCATCA ATCGAAAAAG CAACGTATCT TATTTAAAGT GCGTTGCTTT

2521 TTTCTCATTT ATAAGGTTAA ATAATTCTCA TATATCAAGC AAAGTGACAG GCGCCCTTAA

2581 ATATTCTGAC AAATGCTCTT TCCCTAAACT CCCCCCATAA AAAAACCCGC CGAAGCGGGT

2641 TTTTACGTTA TTTGCGGATT AACGATTACT CGTTATCAGA ACCGCCCAGG GGGCCCGAGC

2701 TTAAGACTGG CCGTCGTTTT ACAACACAGA AAGAGTTTGT AGAAACGCAA AAAGGCCATC

2761 CGTCAGGGGC CTTCTGCTTA GTTTGATGCC TGGCAGTTCC CTACTCTCGC CTTCCGCTTC

2821 CTCGCTCACT GACTCGCTGC GCTCGGTCGT TCGGCTGCGG CGAGCGGTAT CAGCTCACTC

2881 AAAGGCGGTA ATACGGTTAT CCACAGAATC AGGGGATAAC GCAGGAAAGA ACATGTGAGC

2941 AAAAGGCCAG CAAAAGGCCA GGAACCGTAA AAAGGCCGCG TTGCTGGCGT TTTTCCATAG

3001 GCTCCGCCCC CCTGACGAGC ATCACAAAAA TCGACGCTCA AGTCAGAGGT GGCGAAACCC

3061 GACAGGACTA TAAAGATACC AGGCGTTTCC CCCTGGAAGC TCCCTCGTGC GCTCTCCTGT

3121 TCCGACCCTG CCGCTTACCG GATACCTGTC CGCCTTTCTC CCTTCGGGAA GCGTGGCGCT

3181 TTCTCATAGC TCACGCTGTA GGTATCTCAG TTCGGTGTAG GTCGTTCGCT CCAAGCTGGG

3241 CTGTGTGCAC GAACCCCCCG TTCAGCCCGA CCGCTGCGCC TTATCCGGTA ACTATCGTCT

3301 TGAGTCCAAC CCGGTAAGAC ACGACTTATC GCCACTGGCA GCAGCCACTG GTAACAGGAT

3361 TAGCAGAGCG AGGTATGTAG GCGGTGCTAC AGAGTTCTTG AAGTGGTGGG CTAACTACGG

3421 CTACACTAGA AGAACAGTAT TTGGTATCTG CGCTCTGCTG AAGCCAGTTA CCTTCGGAAA

3481 AAGAGTTGGT AGCTCTTGAT CCGGCAAACA AACCACCGCT GGTAGCGGTG GTTTTTTTGT

3541 TTGCAAGCAG CAGATTACGC GCAGAAAAAA AGGATCTCAA GAAGATCCTT TGATCTTTTC

3601 TACGGGGTCT GACGCTCAGT GGAACGACGC GCGCGTAACT CACGTTAAGG GATTTTGGTC

3661 ATGAGCTTGC GCCGTCCCGT CAAGTCAGCG TAATGCTCTG CTTTTACCAA TGCTTAATCA

3721 GTGAGGCACC TATCTCAGCG ATCTGTCTAT TTCGTTCATC CATAGTTGCC TGACTCCCCG

3781 TCGTGTAGAT AACTACGATA CGGGAGGGCT TACCATCTGG CCCCAGCGCT GCGATGATAC

3841 CGCGAGAACC ACGCTCACCG GCTCCGGATT TATCAGCAAT AAACCAGCCA GCCGGAAGGG

3901 CCGAGCGCAG AAGTGGTCCT GCAACTTTAT CCGCCTCCAT CCAGTCTATT AATTGTTGCC

3961 GGGAAGCTAG AGTAAGTAGT TCGCCAGTTA ATAGTTTGCG CAACGTTGTT GCCATCGCTA

4021 CAGGCATCGT GGTGTCACGC TCGTCGTTTG GTATGGCTTC ATTCAGCTCC GGTTCCCAAC

4081 GATCAAGGCG AGTTACATGA TCCCCCATGT TGTGCAAAAA AGCGGTTAGC TCCTTCGGTC

4141 CTCCGATCGT TGTCAGAAGT AAGTTGGCCG CAGTGTTATC ACTCATGGTT ATGGCAGCAC

4201 TGCATAATTC TCTTACTGTC ATGCCATCCG TAAGATGCTT TTCTGTGACT GGTGAGTACT

4261 CAACCAAGTC ATTCTGAGAA TAGTGTATGC GGCGACCGAG TTGCTCTTGC CCGGCGTCAA

4321 TACGGGATAA TACCGCGCCA CATAGCAGAA CTTTAAAAGT GCTCATCATT GGAAAACGTT

4381 CTTCGGGGCG AAAACTCTCA AGGATCTTAC CGCTGTTGAG ATCCAGTTCG ATGTAACCCA

4441 CTCGTGCACC CAACTGATCT TCAGCATCTT TTACTTTCAC CAGCGTTTCT GGGTGAGCAA

4501 AAACAGGAAG GCAAAATGCC GCAAAAAAGG GAATAAGGGC GACACGGAAA TGTTGAATAC

4561 TCATATTCTT CCTTTTTCAA TATTATTGAA GCATTTATCA GGGTTATTGT CTCATGAGCG

4621 GATACATATT TGAATGTATT TAGAAAAATA AACAAATAGG GGTCAGTGTT ACAACCAATT

4681 AACCAATTCT GAACATTATC GCGAGCCCAT TTATACCTGA ATATGGCTCA TAACACCCCT

4741 TGTTTGCCTG GCGGCAGTAG CGCGGTGGTC CCACCTGACC CCATGCCGAA CTCAGAAGTG

4801 AAACGCCGTA GCGCCGATGG TAGTGTGGGG ACTCCCCATG CGAGAGTAGG GAACTGCCAG

4861 GCATCAAATA AAACGAAAGG CTCAGTCGAA AGACTGGGCC TTTCGCCCGG GCTAATTATG

4921 GGGTGTCGCC CTTATTCGAC TCTATAGTGA AGTTCCTATT CTCTAGAAAG TATAGGAACT

4981 TCTGAAGTGG GGATTTAAAG TCGGTGTCCG CGCTGAGGGT TTAATGGCGC GCCGCGGCCG

5041 CCCGCGGTGT TGGAATAAAA ATCCACTATC GTCTATCAAC TAATAGTTAT ATTATCAATA

5101 TATTATCATA TACGGTGTTA AGATGATGAC ATAAGTTATG AGAAGCTGTC ATCGAAGTTA

5161 GAGGAAGCTG AAGTGCAAGG ATTGATAATG TAATAGGATC AATGAATATA AACATATGCT

5221 AGCATTAAGT CCTCAGCGAG CACGCTGCTT CATGGAATGC GTGCGATGAG CGACCTCATG

5281 CTATACCTGA GAAAGCAACC TGACCTACAG GAAAGAGTTA CTCAAGAATA AGAATTTTCG

5341 TTTTAAAACC TAAGAGTCAC TTTAAAATTT GTATACACTT ATTTTTTTTA TAACTTATTT

5401 AATAATAAAA ATCATAAATC ATAAGAAATT CGCTTATTTA GAAGTGTCAA CAACGTATCT

5461 ACCAACGGAA TG

**pCfB2988_(pTY1Cons2-KlURA3-deg) 5469 bp DNA circular UNA**

FEATURES Location/Qualifiers

Misc._feature 1..14

/label="USER cassette"

Terminator 20..190

/label="T CYC1"

Misc._feature 257..316

/label=loxP

Terminator complement(317..439)

/label="T URA3"

Misc._structure complement(440..490)

/label="Degradation tag"

CDS complement(491..1291)

/label="K. lactis URA3syn without STOP codon"

/product="orotidine-5'-phosphate decarboxylase"

/protein_id="AAG34531.1"

/db_xref="GI:11344893"

Promoter complement(1292..1790)

/label="K. lactis URA3 promoter"

Misc._feature 1807..1840

/label=loxP

Misc._feature 1980..2185

/label="TY1 C234 3'"

/label="Consensus TY1Cons2 3'"

Replication_ori complement(2959..3602)

/label="pUC ori"

CDS complement(3705..4563)

/label=AmpR

Misc._feature 5007..5238

/label="TY1 C234 5'"

/label="Consensus TY1Cons2 5'"

Terminator complement(5265..5459)

/label="T ADH1"

ORIGIN

1 CGTGCGATCG CGTGCATTCA TCCGCTCTAA CCGAAAAGGA AGGAGTTAGA CAACCTGAAG

61 TCTAGGTCCC TATTTATTTT TTTATAGTTA TGTTAGTATT AAGAACGTTA TTTATATTTC

121 AAATTTTTCT TTTTTTTCTG TACAGACGCG TGTACGCATG TAACATTATA CTGAAAACCT

181 TGCTTGAGAA GGTTTTGGGA CGCTCGAAGA TCGCGTCAGC TGAAGCTTCG TACGCTGCAG

241 GTCGACAACC CTTAATGTCG ACAACCCTTA ATATAACTTC GTATAATGTA TGCTATACGA

301 AGTTATTAGG TCTAGAGATC CCAATACAAC AGATCACGTG ATCTTTTGTA AGATGAAGTT

361 GAAGTGAGTG TTGCACCGTG CCAATGCAGG TGGCTATTAG ATTAAATATG TGATTTGTTC

421 TATTAAGTTT CCTGTATAAT TACAAATGAA TAACGAAATG AGACAAAGAA GAGAACCAAU

481 TTTTACAAGC ATGGGGAGCG CTGATTCTCT TTTGGTACGC TTCCCATCCA GCATTTCTGT

541 ATCTTTCACC TTCAACCTTA GGATCTCTAC CCTTGGCGAA AAGTCCTCTG CCAACAATGA

601 TGATATCTGA TCCACCACTT ACAACTTCGT CGACGGTTCT GTACTGCTGA CCCAATGCAT

661 CGCCTTTGTC GTCTAAACCT ACACCTGGGG TCATGATTAG CCAATCAAAC CCTTCTTCTC

721 TTCCTCCCAT ATCGTTCTGA GCAATGAACC CAATAACGAA ATCTTTATCA CTCTTTGCAA

781 TATCAACGGT ACCCTTAGTA TATTCACCGT GTGCTAGAGA ACCCTTGGAA GATAATTCAG

841 CAAGCATCAA TAATCCCCTT GGTTCTTTGG TGACCTCTTG CGCACCTTGT TTCAAGCCAG

901 CAACAATACC AGCACCAGTA ACCCCGTGGG CGTTGGTGAT ATCAGACCAT TCTGCGATAC

961 GGTAAACGCC CGATGTATAT TGTAATTTGA CTGTGTTACC GATATCGGCG AATTTTCTGT

1021 CCTCAAATAT CAAGAACTTG TATTTCTCTG CCAATGCTTT CAATGGAACG ACAGTACCCT

1081 CATAACTGAA ATCATCCAAG ATATCAACGT GTGTTTTCAA AAGGCAAATG TATGGACCCA

1141 ACGTTTCAAC AAGTTTCAAT AGCTCATCAG TCGAACGAAC GTCAAGAGAA GCACACAAAT

1201 TGGTTTTCTT TTCATCCATT AAACGTAAAA GTTTCGATGC AACCGGACTT GCATGAGTCT

1261 CAGCTCTACT GGTATATGAT TTTGTGGACA TGGTGCAACT AATTGACGGG AGTGTATTGA

1321 CGCTGGCGTA CTGGCTTTCA CAAAATGGCC CAATCACAAC CACATCTTAG ATAGTTGAAA

1381 TGACTTTAGA TAACATCAAT TGAGATGAGC TTAATCATGT CAAAGCTAAA AGTGTCACCA

1441 TGAACGACAA TTCTTAAGCA AATCACGTGA TATAGATCCA CGAATAACCA CCATTTGATG

1501 CTCGAGGCAA GTAATGTGTG TAAAAAAATG CGTTACCACC ATCCAATGCA GACCGATCTT

1561 CTACCCAGAA TCACATATAT TTATGTACCG AGTACCTTTT TTCTATCTTC CAATTGCTTC

1621 TCCCATATGA TTGTCTCCGT AAGCTCGAAA TTTCTAAGTT GGATTTTAAT CTTCACGCAG

1681 GATGACAGTT CGATGAGCTT CTGAGGAGTG TTTAGAACAT AATCAGTTTA TCCATGGTCT

1741 ATCTCTTCTT GTCGCTTTTT CTCCTCGATA GAACCTAAAT AAAACGAGCT CTCGAGAACC

1801 CTTAATATAA CTTCGTATAA TGTATGCTAT ACGAAGTTAT TAGGTGATAT CAGATCCACT

1861 AGTGGCCTAT GCACCCAATT CGCCCTATAG TGAGTCGTAT TACGCGCGCT CACTGGCCGT

1921 CGTTTTACAA CGTCGTGACT GGGAAAACCC TGGCGTTACC CCTGCAGGAC AGACGTCATT

1981 AGTGCTGAGG CATTAATTGA TCATAAAACG GAATGATGAA TAATATTTAT AGAATTGTGT

2041 AGAATTGCAG ATTCCCTTTT ATGGATTCCT AAATCCTCGA GGAGAACTTC TAGTATATTC

2101 TGTATACCTA ATATTATAGC CTTTATCAAC AATGGAATCC CAACAATTAT CTCAAAATTC

2161 ACATATTTCT CAAGATCTGC GGCCGCACTC AGACCTGAAG TGAAGTTCCT ATACTTTCTA

2221 GAGAATAGGA ACTTCTATAG TGAGTCGAAT AAGGGCGACA CAAAATTTAT TCTAAATGCA

2281 TAATAAATAC TGATAACATC TTATAGTTTG TATTATATTT TGTATTATCG TTGACATGTA

2341 TAATTTTGAT ATCAAAAACT GATTTTCCCT TTATTATTTT CGAGATTTAT TTTCTTAATT

2401 CTCTTTAACA AACTAGAAAT ATTGTATATA CAAAAAATCA TAAATAATAG ATGAATAGTT

2461 TAATTATAGG TGTTCATCAA TCGAAAAAGC AACGTATCTT ATTTAAAGTG CGTTGCTTTT

2521 TTCTCATTTA TAAGGTTAAA TAATTCTCAT ATATCAAGCA AAGTGACAGG CGCCCTTAAA

2581 TATTCTGACA AATGCTCTTT CCCTAAACTC CCCCCATAAA AAAACCCGCC GAAGCGGGTT

2641 TTTACGTTAT TTGCGGATTA ACGATTACTC GTTATCAGAA CCGCCCAGGG GGCCCGAGCT

2701 TAAGACTGGC CGTCGTTTTA CAACACAGAA AGAGTTTGTA GAAACGCAAA AAGGCCATCC

2761 GTCAGGGGCC TTCTGCTTAG TTTGATGCCT GGCAGTTCCC TACTCTCGCC TTCCGCTTCC

2821 TCGCTCACTG ACTCGCTGCG CTCGGTCGTT CGGCTGCGGC GAGCGGTATC AGCTCACTCA

2881 AAGGCGGTAA TACGGTTATC CACAGAATCA GGGGATAACG CAGGAAAGAA CATGTGAGCA

2941 AAAGGCCAGC AAAAGGCCAG GAACCGTAAA AAGGCCGCGT TGCTGGCGTT TTTCCATAGG

3001 CTCCGCCCCC CTGACGAGCA TCACAAAAAT CGACGCTCAA GTCAGAGGTG GCGAAACCCG

3061 ACAGGACTAT AAAGATACCA GGCGTTTCCC CCTGGAAGCT CCCTCGTGCG CTCTCCTGTT

3121 CCGACCCTGC CGCTTACCGG ATACCTGTCC GCCTTTCTCC CTTCGGGAAG CGTGGCGCTT

3181 TCTCATAGCT CACGCTGTAG GTATCTCAGT TCGGTGTAGG TCGTTCGCTC CAAGCTGGGC

3241 TGTGTGCACG AACCCCCCGT TCAGCCCGAC CGCTGCGCCT TATCCGGTAA CTATCGTCTT

3301 GAGTCCAACC CGGTAAGACA CGACTTATCG CCACTGGCAG CAGCCACTGG TAACAGGATT

3361 AGCAGAGCGA GGTATGTAGG CGGTGCTACA GAGTTCTTGA AGTGGTGGGC TAACTACGGC

3421 TACACTAGAA GAACAGTATT TGGTATCTGC GCTCTGCTGA AGCCAGTTAC CTTCGGAAAA

3481 AGAGTTGGTA GCTCTTGATC CGGCAAACAA ACCACCGCTG GTAGCGGTGG TTTTTTTGTT

3541 TGCAAGCAGC AGATTACGCG CAGAAAAAAA GGATCTCAAG AAGATCCTTT GATCTTTTCT

3601 ACGGGGTCTG ACGCTCAGTG GAACGACGCG CGCGTAACTC ACGTTAAGGG ATTTTGGTCA

3661 TGAGCTTGCG CCGTCCCGTC AAGTCAGCGT AATGCTCTGC TTTTACCAAT GCTTAATCAG

3721 TGAGGCACCT ATCTCAGCGA TCTGTCTATT TCGTTCATCC ATAGTTGCCT GACTCCCCGT

3781 CGTGTAGATA ACTACGATAC GGGAGGGCTT ACCATCTGGC CCCAGCGCTG CGATGATACC

3841 GCGAGAACCA CGCTCACCGG CTCCGGATTT ATCAGCAATA AACCAGCCAG CCGGAAGGGC

3901 CGAGCGCAGA AGTGGTCCTG CAACTTTATC CGCCTCCATC CAGTCTATTA ATTGTTGCCG

3961 GGAAGCTAGA GTAAGTAGTT CGCCAGTTAA TAGTTTGCGC AACGTTGTTG CCATCGCTAC

4021 AGGCATCGTG GTGTCACGCT CGTCGTTTGG TATGGCTTCA TTCAGCTCCG GTTCCCAACG

4081 ATCAAGGCGA GTTACATGAT CCCCCATGTT GTGCAAAAAA GCGGTTAGCT CCTTCGGTCC

4141 TCCGATCGTT GTCAGAAGTA AGTTGGCCGC AGTGTTATCA CTCATGGTTA TGGCAGCACT

4201 GCATAATTCT CTTACTGTCA TGCCATCCGT AAGATGCTTT TCTGTGACTG GTGAGTACTC

4261 AACCAAGTCA TTCTGAGAAT AGTGTATGCG GCGACCGAGT TGCTCTTGCC CGGCGTCAAT

4321 ACGGGATAAT ACCGCGCCAC ATAGCAGAAC TTTAAAAGTG CTCATCATTG GAAAACGTTC

4381 TTCGGGGCGA AAACTCTCAA GGATCTTACC GCTGTTGAGA TCCAGTTCGA TGTAACCCAC

4441 TCGTGCACCC AACTGATCTT CAGCATCTTT TACTTTCACC AGCGTTTCTG GGTGAGCAAA

4501 AACAGGAAGG CAAAATGCCG CAAAAAAGGG AATAAGGGCG ACACGGAAAT GTTGAATACT

4561 CATATTCTTC CTTTTTCAAT ATTATTGAAG CATTTATCAG GGTTATTGTC TCATGAGCGG

4621 ATACATATTT GAATGTATTT AGAAAAATAA ACAAATAGGG GTCAGTGTTA CAACCAATTA

4681 ACCAATTCTG AACATTATCG CGAGCCCATT TATACCTGAA TATGGCTCAT AACACCCCTT

4741 GTTTGCCTGG CGGCAGTAGC GCGGTGGTCC CACCTGACCC CATGCCGAAC TCAGAAGTGA

4801 AACGCCGTAG CGCCGATGGT AGTGTGGGGA CTCCCCATGC GAGAGTAGGG AACTGCCAGG

4861 CATCAAATAA AACGAAAGGC TCAGTCGAAA GACTGGGCCT TTCGCCCGGG CTAATTATGG

4921 GGTGTCGCCC TTATTCGACT CTATAGTGAA GTTCCTATTC TCTAGAAAGT ATAGGAACTT

4981 CTGAAGTGGG GATTTAAAGT CGGTGTCCGC GCTGAGGGTT TAATGGCGCG CCGCGGCCGC

5041 CCGCGGTGTT GGAATAAAAA TCAACTATCA TCTACTAACT AGTATTTACG TTACTAGTAT

5101 ATTATCATAT ACGGTGTTAG AAGATGACGC AAATGATGAG AAATAGTCAT CTAAATTAGT

5161 GGAAGCTGAA ACGCAAGGAT TGATAATGTA ATAGGATCAA TGAATATTAA CATAGCTAGC

5221 ATTAAGTCCT CAGCGAGCAC GCTGCTTCAT GGAATGCGTG CGATGAGCGA CCTCATGCTA

5281 TACCTGAGAA AGCAACCTGA CCTACAGGAA AGAGTTACTC AAGAATAAGA ATTTTCGTTT

5341 TAAAACCTAA GAGTCACTTT AAAATTTGTA TACACTTATT TTTTTTATAA CTTATTTAAT

5401 AATAAAAATC ATAAATCATA AGAAATTCGC TTATTTAGAA GTGTCAACAA CGTATCTACC

5461 AACGGAATG

**pCfB2797\(pTY2-KlURA3-deg) 5441 bp DNA circular UNA**

FEATURES Location/Qualifiers

Misc._feature 1..14

/label="USER cassette"

Terminator 20..190

/label="T CYC1"

Misc._feature 238..297

/label=loxP

Terminator complement(298..420)

/label="T URA3"

Misc._structure complement(421..471)

/label="Degradation tag"

CDS complement(472..1272)

/label="K. lactis URA3syn without STOP codon"

/product="orotidine-5'-phosphate decarboxylase"

/protein_id="AAG34531.1"

/db_xref="GI:11344893"

Promoter complement(1273..1771)

/label="K. lactis URA3 promoter"

Misc._feature 1788..1821

/label=loxP

Misc._feature 1961..2147

/label="TY2 3'"

/label="Consensus TY2 3'"

Replication_ori complement(2934..3577)

/label="pUC ori"

CDS complement(3680..4538)

/label=AmpR

Misc._feature 4982..5210

/label="TY2 5'"

/label="Consensus TY2 5'"

Terminator complement(5237..5431)

/label="T ADH1"

ORIGIN

1 CGTGCGATCG CGTGCATTCA TCCGCTCTAA CCGAAAAGGA AGGAGTTAGA CAACCTGAAG

61 TCTAGGTCCC TATTTATTTT TTTATAGTTA TGTTAGTATT AAGAACGTTA TTTATATTTC

121 AAATTTTTCT TTTTTTTCTG TACAGACGCG TGTACGCATG TAACATTATA CTGAAAACCT

181 TGCTTGAGAA ATCGCGTCAG CTGAAGCTTC GTACGCTGCA GGTCGACAAC CCTTAATGTC

241 GACAACCCTT AATATAACTT CGTATAATGT ATGCTATACG AAGTTATTAG GTCTAGAGAT

301 CCCAATACAA CAGATCACGT GATCTTTTGT AAGATGAAGT TGAAGTGAGT GTTGCACCGT

361 GCCAATGCAG GTGGCTATTA GATTAAATAT GTGATTTGTT CTATTAAGTT TCCTGTATAA

421 TTACAAATGA ATAACGAAAT GAGACAAAGA AGAGAACCAA UTTTTACAAG CATGGGGAGC

481 GCTGATTCTC TTTTGGTACG CTTCCCATCC AGCATTTCTG TATCTTTCAC CTTCAACCTT

541 AGGATCTCTA CCCTTGGCGA AAAGTCCTCT GCCAACAATG ATGATATCTG ATCCACCACT

601 TACAACTTCG TCGACGGTTC TGTACTGCTG ACCCAATGCA TCGCCTTTGT CGTCTAAACC

661 TACACCTGGG GTCATGATTA GCCAATCAAA CCCTTCTTCT CTTCCTCCCA TATCGTTCTG

721 AGCAATGAAC CCAATAACGA AATCTTTATC ACTCTTTGCA ATATCAACGG TACCCTTAGT

781 ATATTCACCG TGTGCTAGAG AACCCTTGGA AGATAATTCA GCAAGCATCA ATAATCCCCT

841 TGGTTCTTTG GTGACCTCTT GCGCACCTTG TTTCAAGCCA GCAACAATAC CAGCACCAGT

901 AACCCCGTGG GCGTTGGTGA TATCAGACCA TTCTGCGATA CGGTAAACGC CCGATGTATA

961 TTGTAATTTG ACTGTGTTAC CGATATCGGC GAATTTTCTG TCCTCAAATA TCAAGAACTT

1021 GTATTTCTCT GCCAATGCTT TCAATGGAAC GACAGTACCC TCATAACTGA AATCATCCAA

1081 GATATCAACG TGTGTTTTCA AAAGGCAAAT GTATGGACCC AACGTTTCAA CAAGTTTCAA

1141 TAGCTCATCA GTCGAACGAA CGTCAAGAGA AGCACACAAA TTGGTTTTCT TTTCATCCAT

1201 TAAACGTAAA AGTTTCGATG CAACCGGACT TGCATGAGTC TCAGCTCTAC TGGTATATGA

1261 TTTTGTGGAC ATGGTGCAAC TAATTGACGG GAGTGTATTG ACGCTGGCGT ACTGGCTTTC

1321 ACAAAATGGC CCAATCACAA CCACATCTTA GATAGTTGAA ATGACTTTAG ATAACATCAA

1381 TTGAGATGAG CTTAATCATG TCAAAGCTAA AAGTGTCACC ATGAACGACA ATTCTTAAGC

1441 AAATCACGTG ATATAGATCC ACGAATAACC ACCATTTGAT GCTCGAGGCA AGTAATGTGT

1501 GTAAAAAAAT GCGTTACCAC CATCCAATGC AGACCGATCT TCTACCCAGA ATCACATATA

1561 TTTATGTACC GAGTACCTTT TTTCTATCTT CCAATTGCTT CTCCCATATG ATTGTCTCCG

1621 TAAGCTCGAA ATTTCTAAGT TGGATTTTAA TCTTCACGCA GGATGACAGT TCGATGAGCT

1681 TCTGAGGAGT GTTTAGAACA TAATCAGTTT ATCCATGGTC TATCTCTTCT TGTCGCTTTT

1741 TCTCCTCGAT AGAACCTAAA TAAAACGAGC TCTCGAGAAC CCTTAATATA ACTTCGTATA

1801 ATGTATGCTA TACGAAGTTA TTAGGTGATA TCAGATCCAC TAGTGGCCTA TGCACCCAAT

1861 TCGCCCTATA GTGAGTCGTA TTACGCGCGC TCACTGGCCG TCGTTTTACA ACGTCGTGAC

1921 TGGGAAAACC CTGGCGTTAC CCCTGCAGGA CAGACGTCAT TAGTGCTGAG GCATTAATTG

1981 ATCAATATAA AATGATGATA ATAATATTTA TAGAATTGTG TAGAATTGCA GATTCCCTTT

2041 TATGGATTCC TAAATCCTGA GGAGAACTTC TAGTATATTC TACATACCTA ATATTATTGC

2101 CTTATTAAAA ATGGAATCCC AACAATTACA TCAAAATCCA CATTCTCAGA TCTGCGGCCG

2161 CACTCAGACC TGAAGTGAAG TTCCTATACT TTCTAGAGAA TAGGAACTTC TATAGTGAGT

2221 CGAATAAGGG CGACACAAAA TTTATTCTAA ATGCATAATA AATACTGATA ACATCTTATA

2281 GTTTGTATTA TATTTTGTAT TATCGTTGAC ATGTATAATT TTGATATCAA AAACTGATTT

2341 TCCCTTTATT ATTTTCGAGA TTTATTTTCT TAATTCTCTT TAACAAACTA GAAATATTGT

2401 ATATACAAAA AATCATAAAT AATAGATGAA TAGTTTAATT ATAGGTGTTC ATCAATCGAA

2461 AAAGCAACGT ATCTTATTTA AAGTGCGTTG CTTTTTTCTC ATTTATAAGG TTAAATAATT

2521 CTCATATATC AAGCAAAGTG ACAGGCGCCC TTAAATATTC TGACAAATGC TCTTTCCCTA

2581 AACTCCCCCC ATAAAAAAAC CCGCCGAAGC GGGTTTTTAC GTTATTTGCG GATTAACGAT

2641 TACTCGTTAT CAGAACCGCC CAGGGGGCCC GAGCTTAAGA CTGGCCGTCG TTTTACAACA

2701 CAGAAAGAGT TTGTAGAAAC GCAAAAAGGC CATCCGTCAG GGGCCTTCTG CTTAGTTTGA

2761 TGCCTGGCAG TTCCCTACTC TCGCCTTCCG CTTCCTCGCT CACTGACTCG CTGCGCTCGG

2821 TCGTTCGGCT GCGGCGAGCG GTATCAGCTC ACTCAAAGGC GGTAATACGG TTATCCACAG

2881 AATCAGGGGA TAACGCAGGA AAGAACATGT GAGCAAAAGG CCAGCAAAAG GCCAGGAACC

2941 GTAAAAAGGC CGCGTTGCTG GCGTTTTTCC ATAGGCTCCG CCCCCCTGAC GAGCATCACA

3001 AAAATCGACG CTCAAGTCAG AGGTGGCGAA ACCCGACAGG ACTATAAAGA TACCAGGCGT

3061 TTCCCCCTGG AAGCTCCCTC GTGCGCTCTC CTGTTCCGAC CCTGCCGCTT ACCGGATACC

3121 TGTCCGCCTT TCTCCCTTCG GGAAGCGTGG CGCTTTCTCA TAGCTCACGC TGTAGGTATC

3181 TCAGTTCGGT GTAGGTCGTT CGCTCCAAGC TGGGCTGTGT GCACGAACCC CCCGTTCAGC

3241 CCGACCGCTG CGCCTTATCC GGTAACTATC GTCTTGAGTC CAACCCGGTA AGACACGACT

3301 TATCGCCACT GGCAGCAGCC ACTGGTAACA GGATTAGCAG AGCGAGGTAT GTAGGCGGTG

3361 CTACAGAGTT CTTGAAGTGG TGGGCTAACT ACGGCTACAC TAGAAGAACA GTATTTGGTA

3421 TCTGCGCTCT GCTGAAGCCA GTTACCTTCG GAAAAAGAGT TGGTAGCTCT TGATCCGGCA

3481 AACAAACCAC CGCTGGTAGC GGTGGTTTTT TTGTTTGCAA GCAGCAGATT ACGCGCAGAA

3541 AAAAAGGATC TCAAGAAGAT CCTTTGATCT TTTCTACGGG GTCTGACGCT CAGTGGAACG

3601 ACGCGCGCGT AACTCACGTT AAGGGATTTT GGTCATGAGC TTGCGCCGTC CCGTCAAGTC

3661 AGCGTAATGC TCTGCTTTTA CCAATGCTTA ATCAGTGAGG CACCTATCTC AGCGATCTGT

3721 CTATTTCGTT CATCCATAGT TGCCTGACTC CCCGTCGTGT AGATAACTAC GATACGGGAG

3781 GGCTTACCAT CTGGCCCCAG CGCTGCGATG ATACCGCGAG AACCACGCTC ACCGGCTCCG

3841 GATTTATCAG CAATAAACCA GCCAGCCGGA AGGGCCGAGC GCAGAAGTGG TCCTGCAACT

3901 TTATCCGCCT CCATCCAGTC TATTAATTGT TGCCGGGAAG CTAGAGTAAG TAGTTCGCCA

3961 GTTAATAGTT TGCGCAACGT TGTTGCCATC GCTACAGGCA TCGTGGTGTC ACGCTCGTCG

4021 TTTGGTATGG CTTCATTCAG CTCCGGTTCC CAACGATCAA GGCGAGTTAC ATGATCCCCC

4081 ATGTTGTGCA AAAAAGCGGT TAGCTCCTTC GGTCCTCCGA TCGTTGTCAG AAGTAAGTTG

4141 GCCGCAGTGT TATCACTCAT GGTTATGGCA GCACTGCATA ATTCTCTTAC TGTCATGCCA

4201 TCCGTAAGAT GCTTTTCTGT GACTGGTGAG TACTCAACCA AGTCATTCTG AGAATAGTGT

4261 ATGCGGCGAC CGAGTTGCTC TTGCCCGGCG TCAATACGGG ATAATACCGC GCCACATAGC

4321 AGAACTTTAA AAGTGCTCAT CATTGGAAAA CGTTCTTCGG GGCGAAAACT CTCAAGGATC

4381 TTACCGCTGT TGAGATCCAG TTCGATGTAA CCCACTCGTG CACCCAACTG ATCTTCAGCA

4441 TCTTTTACTT TCACCAGCGT TTCTGGGTGA GCAAAAACAG GAAGGCAAAA TGCCGCAAAA

4501 AAGGGAATAA GGGCGACACG GAAATGTTGA ATACTCATAT TCTTCCTTTT TCAATATTAT

4561 TGAAGCATTT ATCAGGGTTA TTGTCTCATG AGCGGATACA TATTTGAATG TATTTAGAAA

4621 AATAAACAAA TAGGGGTCAG TGTTACAACC AATTAACCAA TTCTGAACAT TATCGCGAGC

4681 CCATTTATAC CTGAATATGG CTCATAACAC CCCTTGTTTG CCTGGCGGCA GTAGCGCGGT

4741 GGTCCCACCT GACCCCATGC CGAACTCAGA AGTGAAACGC CGTAGCGCCG ATGGTAGTGT

4801 GGGGACTCCC CATGCGAGAG TAGGGAACTG CCAGGCATCA AATAAAACGA AAGGCTCAGT

4861 CGAAAGACTG GGCCTTTCGC CCGGGCTAAT TATGGGGTGT CGCCCTTATT CGACTCTATA

4921 GTGAAGTTCC TATTCTCTAG AAAGTATAGG AACTTCTGAA GTGGGGATTT AAAGTCGGTG

4981 TCCGCGCTGA GGGTTTAATG GCGCGCCGCG GCCGCCCGCG GTGTTGGAAT AAAAATCAAC

5041 TATCATCTAC TAACTAGTAT TTACGTTACT AGTATATTAT CATATACGGT GTTAGAAGAT

5101 GACGCAAATG ATGAGAAATA GTCATCTAAA TTAGTGGAAG CTGAAACGCA AGGATTGATA

5161 ATGTAATAGG ATCAATGAAT ATTAACGCTA GCATTAAGTC CTCAGCGAGC ACGCTGCTTC

5221 ATGGAATGCG TGCGATGAGC GACCTCATGC TATACCTGAG AAAGCAACCT GACCTACAGG

5281 AAAGAGTTAC TCAAGAATAA GAATTTTCGT TTTAAAACCT AAGAGTCACT TTAAAATTTG

5341 TATACACTTA TTTTTTTTAT AACTTATTTA ATAATAAAAA TCATAAATCA TAAGAAATTC

5401 GCTTATTTAG AAGTGTCAAC AACGTATCTA CCAACGGAAT G

**pCfB2875_(pTY3-KlURA3-deg) 5473 bp DNA circular UNA**

FEATURES Location/Qualifiers

Misc._feature 1..14

/label="USER cassette"

Terminator 20..190

/label="T CYC1"

Misc._feature 257..316

/label=loxP

Terminator complement(317..439)

/label="T URA3"

Misc._structure complement(440..490)

/label="Degradation tag"

CDS complement(491..1291)

/label="K. lactis URA3syn without STOP codon"

/product="orotidine-5'-phosphate decarboxylase"

/protein_id="AAG34531.1"

/db_xref="GI:11344893"

Promoter complement(1292..1790)

/label="K. lactis URA3 promoter"

Misc._feature 1807..1840

/label=loxP

Misc._feature 1980..2180

/label="TY3 3'"

/label="Consensus TY3 3'"

Replication_ori complement(2961..3604)

/label="pUC ori"

CDS complement(3707..4565)

/label=AmpR

Misc._feature 5009..5242

/label="TY3 5'"

/label="Consensus TY3 5'"

Terminator complement(5269..5463)

/label="T ADH1"

ORIGIN

1 CGTGCGATCG CGTGCATTCA TCCGCTCTAA CCGAAAAGGA AGGAGTTAGA CAACCTGAAG

61 TCTAGGTCCC TATTTATTTT TTTATAGTTA TGTTAGTATT AAGAACGTTA TTTATATTTC

121 AAATTTTTCT TTTTTTTCTG TACAGACGCG TGTACGCATG TAACATTATA CTGAAAACCT

181 TGCTTGAGAA GGTTTTGGGA CGCTCGAAGA TCGCGTCAGC TGAAGCTTCG TACGCTGCAG

241 GTCGACAACC CTTAATGTCG ACAACCCTTA ATATAACTTC GTATAATGTA TGCTATACGA

301 AGTTATTAGG TCTAGAGATC CCAATACAAC AGATCACGTG ATCTTTTGTA AGATGAAGTT

361 GAAGTGAGTG TTGCACCGTG CCAATGCAGG TGGCTATTAG ATTAAATATG TGATTTGTTC

421 TATTAAGTTT CCTGTATAAT TACAAATGAA TAACGAAATG AGACAAAGAA GAGAACCAAU

481 TTTTACAAGC ATGGGGAGCG CTGATTCTCT TTTGGTACGC TTCCCATCCA GCATTTCTGT

541 ATCTTTCACC TTCAACCTTA GGATCTCTAC CCTTGGCGAA AAGTCCTCTG CCAACAATGA

601 TGATATCTGA TCCACCACTT ACAACTTCGT CGACGGTTCT GTACTGCTGA CCCAATGCAT

661 CGCCTTTGTC GTCTAAACCT ACACCTGGGG TCATGATTAG CCAATCAAAC CCTTCTTCTC

721 TTCCTCCCAT ATCGTTCTGA GCAATGAACC CAATAACGAA ATCTTTATCA CTCTTTGCAA

781 TATCAACGGT ACCCTTAGTA TATTCACCGT GTGCTAGAGA ACCCTTGGAA GATAATTCAG

841 CAAGCATCAA TAATCCCCTT GGTTCTTTGG TGACCTCTTG CGCACCTTGT TTCAAGCCAG

901 CAACAATACC AGCACCAGTA ACCCCGTGGG CGTTGGTGAT ATCAGACCAT TCTGCGATAC

961 GGTAAACGCC CGATGTATAT TGTAATTTGA CTGTGTTACC GATATCGGCG AATTTTCTGT

1021 CCTCAAATAT CAAGAACTTG TATTTCTCTG CCAATGCTTT CAATGGAACG ACAGTACCCT

1081 CATAACTGAA ATCATCCAAG ATATCAACGT GTGTTTTCAA AAGGCAAATG TATGGACCCA

1141 ACGTTTCAAC AAGTTTCAAT AGCTCATCAG TCGAACGAAC GTCAAGAGAA GCACACAAAT

1201 TGGTTTTCTT TTCATCCATT AAACGTAAAA GTTTCGATGC AACCGGACTT GCATGAGTCT

1261 CAGCTCTACT GGTATATGAT TTTGTGGACA TGGTGCAACT AATTGACGGG AGTGTATTGA

1321 CGCTGGCGTA CTGGCTTTCA CAAAATGGCC CAATCACAAC CACATCTTAG ATAGTTGAAA

1381 TGACTTTAGA TAACATCAAT TGAGATGAGC TTAATCATGT CAAAGCTAAA AGTGTCACCA

1441 TGAACGACAA TTCTTAAGCA AATCACGTGA TATAGATCCA CGAATAACCA CCATTTGATG

1501 CTCGAGGCAA GTAATGTGTG TAAAAAAATG CGTTACCACC ATCCAATGCA GACCGATCTT

1561 CTACCCAGAA TCACATATAT TTATGTACCG AGTACCTTTT TTCTATCTTC CAATTGCTTC

1621 TCCCATATGA TTGTCTCCGT AAGCTCGAAA TTTCTAAGTT GGATTTTAAT CTTCACGCAG

1681 GATGACAGTT CGATGAGCTT CTGAGGAGTG TTTAGAACAT AATCAGTTTA TCCATGGTCT

1741 ATCTCTTCTT GTCGCTTTTT CTCCTCGATA GAACCTAAAT AAAACGAGCT CTCGAGAACC

1801 CTTAATATAA CTTCGTATAA TGTATGCTAT ACGAAGTTAT TAGGTGATAT CAGATCCACT

1861 AGTGGCCTAT GCACCCAATT CGCCCTATAG TGAGTCGTAT TACGCGCGCT CACTGGCCGT

1921 CGTTTTACAA CGTCGTGACT GGGAAAACCC TGGCGTTACC CCTGCAGGAC AGACGTCATT

1981 AGTGCTGAGG CATTAATTGA TCAGATTCCG CGCTTCCACC ACTTAGTATG ATTCATATTT

2041 TATATAATAT ATAAGATAAG TAACATTCCG TGAATTAATC TGATAAACTG TTTTGACAAC

2101 TGGTTACTTC CCTAAGACTG TTTATATTAG GATTGTCAAG ACACTCCGGT ATTACTCGAG

2161 CCCGTAATAC AACAAGATCT GCGGCCGCAC TCAGACCTGA AGTGAAGTTC CTATACTTTC

2221 TAGAGAATAG GAACTTCTAT AGTGAGTCGA ATAAGGGCGA CACAAAATTT ATTCTAAATG

2281 CATAATAAAT ACTGATAACA TCTTATAGTT TGTATTATAT TTTGTATTAT CGTTGACATG

2341 TATAATTTTG ATATCAAAAA CTGATTTTCC CTTTATTATT TTCGAGATTT ATTTTCTTAA

2401 TTCTCTTTAA CAAACTAGAA ATATTGTATA TACAAAAAAT CATAAATAAT AGATGAATAG

2461 TTTAATTATA GGTGTTCATC AATCGAAAAA GCAACGTATC TTATTTAAAG TGCGTTGCTT

2521 TTTTCTCATT TATAAGGTTA AATAATTCTC ATATATCAAG CAAAGTGACA GGCGCCCTTA

2581 AATATTCTGA CAAATGCTCT TTCCCTAAAC TCCCCCCATA AAAAAACCCG CCGAAGCGGG

2641 TTTTTACGTT ATTTGCGGAT TAACGATTAC TCGTTATCAG AACCGCCCAG GGGGCCCGAG

2701 CTTAAGACTG GCCGTCGTTT TACAACACAG AAAGAGTTTG TAGAAACGCA AAAAGGCCAT

2761 CCGTCAGGGG CCTTCTGCTT AGTTTGATGC CTGGCAGTTC CCTACTCTCG CCTTCCGCTT

2821 CCTCGCTCAC TGACTCGCTG CGCTCGGTCG TTCGGCTGCG GCGAGCGGTA TCAGCTCACT

2881 CAAAGGCGGT AATACGGTTA TCCACAGAAT CAGGGGATAA CGCAGGAAAG AACATGTGAG

2941 CAAAAGGCCA GCAAAAGGCC AGGAACCGTA AAAAGGCCGC GTTGCTGGCG TTTTTCCATA

3001 GGCTCCGCCC CCCTGACGAG CATCACAAAA ATCGACGCTC AAGTCAGAGG TGGCGAAACC

3061 CGACAGGACT ATAAAGATAC CAGGCGTTTC CCCCTGGAAG CTCCCTCGTG CGCTCTCCTG

3121 TTCCGACCCT GCCGCTTACC GGATACCTGT CCGCCTTTCT CCCTTCGGGA AGCGTGGCGC

3181 TTTCTCATAG CTCACGCTGT AGGTATCTCA GTTCGGTGTA GGTCGTTCGC TCCAAGCTGG

3241 GCTGTGTGCA CGAACCCCCC GTTCAGCCCG ACCGCTGCGC CTTATCCGGT AACTATCGTC

3301 TTGAGTCCAA CCCGGTAAGA CACGACTTAT CGCCACTGGC AGCAGCCACT GGTAACAGGA

3361 TTAGCAGAGC GAGGTATGTA GGCGGTGCTA CAGAGTTCTT GAAGTGGTGG GCTAACTACG

3421 GCTACACTAG AAGAACAGTA TTTGGTATCT GCGCTCTGCT GAAGCCAGTT ACCTTCGGAA

3481 AAAGAGTTGG TAGCTCTTGA TCCGGCAAAC AAACCACCGC TGGTAGCGGT GGTTTTTTTG

3541 TTTGCAAGCA GCAGATTACG CGCAGAAAAA AAGGATCTCA AGAAGATCCT TTGATCTTTT

3601 CTACGGGGTC TGACGCTCAG TGGAACGACG CGCGCGTAAC TCACGTTAAG GGATTTTGGT

3661 CATGAGCTTG CGCCGTCCCG TCAAGTCAGC GTAATGCTCT GCTTTTACCA ATGCTTAATC

3721 AGTGAGGCAC CTATCTCAGC GATCTGTCTA TTTCGTTCAT CCATAGTTGC CTGACTCCCC

3781 GTCGTGTAGA TAACTACGAT ACGGGAGGGC TTACCATCTG GCCCCAGCGC TGCGATGATA

3841 CCGCGAGAAC CACGCTCACC GGCTCCGGAT TTATCAGCAA TAAACCAGCC AGCCGGAAGG

3901 GCCGAGCGCA GAAGTGGTCC TGCAACTTTA TCCGCCTCCA TCCAGTCTAT TAATTGTTGC

3961 CGGGAAGCTA GAGTAAGTAG TTCGCCAGTT AATAGTTTGC GCAACGTTGT TGCCATCGCT

4021 ACAGGCATCG TGGTGTCACG CTCGTCGTTT GGTATGGCTT CATTCAGCTC CGGTTCCCAA

4081 CGATCAAGGC GAGTTACATG ATCCCCCATG TTGTGCAAAA AAGCGGTTAG CTCCTTCGGT

4141 CCTCCGATCG TTGTCAGAAG TAAGTTGGCC GCAGTGTTAT CACTCATGGT TATGGCAGCA

4201 CTGCATAATT CTCTTACTGT CATGCCATCC GTAAGATGCT TTTCTGTGAC TGGTGAGTAC

4261 TCAACCAAGT CATTCTGAGA ATAGTGTATG CGGCGACCGA GTTGCTCTTG CCCGGCGTCA

4321 ATACGGGATA ATACCGCGCC ACATAGCAGA ACTTTAAAAG TGCTCATCAT TGGAAAACGT

4381 TCTTCGGGGC GAAAACTCTC AAGGATCTTA CCGCTGTTGA GATCCAGTTC GATGTAACCC

4441 ACTCGTGCAC CCAACTGATC TTCAGCATCT TTTACTTTCA CCAGCGTTTC TGGGTGAGCA

4501 AAAACAGGAA GGCAAAATGC CGCAAAAAAG GGAATAAGGG CGACACGGAA ATGTTGAATA

4561 CTCATATTCT TCCTTTTTCA ATATTATTGA AGCATTTATC AGGGTTATTG TCTCATGAGC

4621 GGATACATAT TTGAATGTAT TTAGAAAAAT AAACAAATAG GGGTCAGTGT TACAACCAAT

4681 TAACCAATTC TGAACATTAT CGCGAGCCCA TTTATACCTG AATATGGCTC ATAACACCCC

4741 TTGTTTGCCT GGCGGCAGTA GCGCGGTGGT CCCACCTGAC CCCATGCCGA ACTCAGAAGT

4801 GAAACGCCGT AGCGCCGATG GTAGTGTGGG GACTCCCCAT GCGAGAGTAG GGAACTGCCA

4861 GGCATCAAAT AAAACGAAAG GCTCAGTCGA AAGACTGGGC CTTTCGCCCG GGCTAATTAT

4921 GGGGTGTCGC CCTTATTCGA CTCTATAGTG AAGTTCCTAT TCTCTAGAAA GTATAGGAAC

4981 TTCTGAAGTG GGGATTTAAA GTCGGTGTCC GCGCTGAGGG TTTAATGGCG CGCCGCGGCC

5041 GCCCGCGGTG TTGTATCTCA AAATGAGATA TGTCAGTATG ACAATACGTC ATCCTGAACG

5101 TTCATAAAAC ACATATGAAA CAACCTTATA ACAAAACGAA CAACATGAGA CAAAACCCGT

5161 CCTTCCCTAG CTGAACTACC CAAAAGTATA AATGCCTGAA CAATTAGTTT AGATCCGAGC

5221 TAGCATTAAG TCCTCAGCGA GCACGCTGCT TCATGGAATG CGTGCGATGA GCGACCTCAT

5281 GCTATACCTG AGAAAGCAAC CTGACCTACA GGAAAGAGTT ACTCAAGAAT AAGAATTTTC

5341 GTTTTAAAAC CTAAGAGTCA CTTTAAAATT TGTATACACT TATTTTTTTT ATAACTTATT

5401 TAATAATAAA AATCATAAAT CATAAGAAAT TCGCTTATTT AGAAGTGTCA ACAACGTATC

5461 TACCAACGGA ATG

**pCfB2796\(pTY4-KlURA3-deg) 5496 bp DNA circular UNA**

FEATURES Location/Qualifiers

Misc._feature 1..14

/label="USER cassette"

Terminator 20..209

/label="T CYC1"

Misc._feature 257..316

/label=loxP

Terminator complement(317..439)

/label="T URA3"

Misc._structure complement(440..490)

/label="Degradation tag"

CDS complement(491..1291)

/label="K. lactis URA3syn without STOP codon"

/product="orotidine-5'-phosphate decarboxylase"

/protein_id="AAG34531.1"

/db_xref="GI:11344893"

Promoter complement(1292..1790)

/label="K. lactis URA3 promoter"

Misc._feature 1807..1840

/label=loxP

Misc._feature 1995..2180

/label="Consensus TY4 3'"

Replication_ori complement(2978..3621)

/label="pUC ori"

CDS complement(3724..4582)

/label=AmpR

Misc._feature 5063..5247

/label="Consensus TY4 5'"

Terminator complement(5292..5486)

/label="T ADH1"

ORIGIN

1 CGTGCGATCG CGTGCATTCA TCCGCTCTAA CCGAAAAGGA AGGAGTTAGA CAACCTGAAG

61 TCTAGGTCCC TATTTATTTT TTTATAGTTA TGTTAGTATT AAGAACGTTA TTTATATTTC

121 AAATTTTTCT TTTTTTTCTG TACAGACGCG TGTACGCATG TAACATTATA CTGAAAACCT

181 TGCTTGAGAA GGTTTTGGGA CGCTCGAAGA TCGCGTCAGC TGAAGCTTCG TACGCTGCAG

241 GTCGACAACC CTTAATGTCG ACAACCCTTA ATATAACTTC GTATAATGTA TGCTATACGA

301 AGTTATTAGG TCTAGAGATC CCAATACAAC AGATCACGTG ATCTTTTGTA AGATGAAGTT

361 GAAGTGAGTG TTGCACCGTG CCAATGCAGG TGGCTATTAG ATTAAATATG TGATTTGTTC

421 TATTAAGTTT CCTGTATAAT TACAAATGAA TAACGAAATG AGACAAAGAA GAGAACCAAU

481 TTTTACAAGC ATGGGGAGCG CTGATTCTCT TTTGGTACGC TTCCCATCCA GCATTTCTGT

541 ATCTTTCACC TTCAACCTTA GGATCTCTAC CCTTGGCGAA AAGTCCTCTG CCAACAATGA

601 TGATATCTGA TCCACCACTT ACAACTTCGT CGACGGTTCT GTACTGCTGA CCCAATGCAT

661 CGCCTTTGTC GTCTAAACCT ACACCTGGGG TCATGATTAG CCAATCAAAC CCTTCTTCTC

721 TTCCTCCCAT ATCGTTCTGA GCAATGAACC CAATAACGAA ATCTTTATCA CTCTTTGCAA

781 TATCAACGGT ACCCTTAGTA TATTCACCGT GTGCTAGAGA ACCCTTGGAA GATAATTCAG

841 CAAGCATCAA TAATCCCCTT GGTTCTTTGG TGACCTCTTG CGCACCTTGT TTCAAGCCAG

901 CAACAATACC AGCACCAGTA ACCCCGTGGG CGTTGGTGAT ATCAGACCAT TCTGCGATAC

961 GGTAAACGCC CGATGTATAT TGTAATTTGA CTGTGTTACC GATATCGGCG AATTTTCTGT

1021 CCTCAAATAT CAAGAACTTG TATTTCTCTG CCAATGCTTT CAATGGAACG ACAGTACCCT

1081 CATAACTGAA ATCATCCAAG ATATCAACGT GTGTTTTCAA AAGGCAAATG TATGGACCCA

1141 ACGTTTCAAC AAGTTTCAAT AGCTCATCAG TCGAACGAAC GTCAAGAGAA GCACACAAAT

1201 TGGTTTTCTT TTCATCCATT AAACGTAAAA GTTTCGATGC AACCGGACTT GCATGAGTCT

1261 CAGCTCTACT GGTATATGAT TTTGTGGACA TGGTGCAACT AATTGACGGG AGTGTATTGA

1321 CGCTGGCGTA CTGGCTTTCA CAAAATGGCC CAATCACAAC CACATCTTAG ATAGTTGAAA

1381 TGACTTTAGA TAACATCAAT TGAGATGAGC TTAATCATGT CAAAGCTAAA AGTGTCACCA

1441 TGAACGACAA TTCTTAAGCA AATCACGTGA TATAGATCCA CGAATAACCA CCATTTGATG

1501 CTCGAGGCAA GTAATGTGTG TAAAAAAATG CGTTACCACC ATCCAATGCA GACCGATCTT

1561 CTACCCAGAA TCACATATAT TTATGTACCG AGTACCTTTT TTCTATCTTC CAATTGCTTC

1621 TCCCATATGA TTGTCTCCGT AAGCTCGAAA TTTCTAAGTT GGATTTTAAT CTTCACGCAG

1681 GATGACAGTT CGATGAGCTT CTGAGGAGTG TTTAGAACAT AATCAGTTTA TCCATGGTCT

1741 ATCTCTTCTT GTCGCTTTTT CTCCTCGATA GAACCTAAAT AAAACGAGCT CTCGAGAACC

1801 CTTAATATAA CTTCGTATAA TGTATGCTAT ACGAAGTTAT TAGGTGATAT CAGATCCACT

1861 AGTGGCCTAT GCACCCAATT CGCCCTATAG TGAGTCGTAT TACGCGCGCT CACTGGCCGT

1921 CGTTTTACAA CGTCGTGACT GGGAAAACCC TGGCGTTACC CCTGCAGGAC TAGTGCTGAG

1981 GCATTAATTG ATCAGGTAGG TACATATATG AGGAATATGA GTCGTCACAT CAATGTATAG

2041 TAACTACCGG AATCACTATT ATATTGGTCA TGATTAATAT GACCAATCGG CGTGTGTTTT

2101 ATATACCTCT CTTATTTAGT ATAAGAAGAT CAGTACTCAC TTCTTCATTA ATACTAATTT

2161 TTAACCTCTA ATTATCAACA AGATCTGCGG CCGCGGCCGC AAATTTAAAT AAAATGAAGT

2221 GAAGTTCCTA TACTTTCTAG AGAATAGGAA CTTCTATAGT GAGTCGAATA AGGGCGACAC

2281 AAAATTTATT CTAAATGCAT AATAAATACT GATAACATCT TATAGTTTGT ATTATATTTT

2341 GTATTATCGT TGACATGTAT AATTTTGATA TCAAAAACTG ATTTTCCCTT TATTATTTTC

2401 GAGATTTATT TTCTTAATTC TCTTTAACAA ACTAGAAATA TTGTATATAC AAAAAATCAT

2461 AAATAATAGA TGAATAGTTT AATTATAGGT GTTCATCAAT CGAAAAAGCA ACGTATCTTA

2521 TTTAAAGTGC GTTGCTTTTT TCTCATTTAT AAGGTTAAAT AATTCTCATA TATCAAGCAA

2581 AGTGACAGGC GCCCTTAAAT ATTCTGACAA ATGCTCTTTC CCTAAACTCC CCCCATAAAA

2641 AAACCCGCCG AAGCGGGTTT TTACGTTATT TGCGGATTAA CGATTACTCG TTATCAGAAC

2701 CGCCCAGGGG GCCCGAGCTT AAGACTGGCC GTCGTTTTAC AACACAGAAA GAGTTTGTAG

2761 AAACGCAAAA AGGCCATCCG TCAGGGGCCT TCTGCTTAGT TTGATGCCTG GCAGTTCCCT

2821 ACTCTCGCCT TCCGCTTCCT CGCTCACTGA CTCGCTGCGC TCGGTCGTTC GGCTGCGGCG

2881 AGCGGTATCA GCTCACTCAA AGGCGGTAAT ACGGTTATCC ACAGAATCAG GGGATAACGC

2941 AGGAAAGAAC ATGTGAGCAA AAGGCCAGCA AAAGGCCAGG AACCGTAAAA AGGCCGCGTT

3001 GCTGGCGTTT TTCCATAGGC TCCGCCCCCC TGACGAGCAT CACAAAAATC GACGCTCAAG

3061 TCAGAGGTGG CGAAACCCGA CAGGACTATA AAGATACCAG GCGTTTCCCC CTGGAAGCTC

3121 CCTCGTGCGC TCTCCTGTTC CGACCCTGCC GCTTACCGGA TACCTGTCCG CCTTTCTCCC

3181 TTCGGGAAGC GTGGCGCTTT CTCATAGCTC ACGCTGTAGG TATCTCAGTT CGGTGTAGGT

3241 CGTTCGCTCC AAGCTGGGCT GTGTGCACGA ACCCCCCGTT CAGCCCGACC GCTGCGCCTT

3301 ATCCGGTAAC TATCGTCTTG AGTCCAACCC GGTAAGACAC GACTTATCGC CACTGGCAGC

3361 AGCCACTGGT AACAGGATTA GCAGAGCGAG GTATGTAGGC GGTGCTACAG AGTTCTTGAA

3421 GTGGTGGGCT AACTACGGCT ACACTAGAAG AACAGTATTT GGTATCTGCG CTCTGCTGAA

3481 GCCAGTTACC TTCGGAAAAA GAGTTGGTAG CTCTTGATCC GGCAAACAAA CCACCGCTGG

3541 TAGCGGTGGT TTTTTTGTTT GCAAGCAGCA GATTACGCGC AGAAAAAAAG GATCTCAAGA

3601 AGATCCTTTG ATCTTTTCTA CGGGGTCTGA CGCTCAGTGG AACGACGCGC GCGTAACTCA

3661 CGTTAAGGGA TTTTGGTCAT GAGCTTGCGC CGTCCCGTCA AGTCAGCGTA ATGCTCTGCT

3721 TTTACCAATG CTTAATCAGT GAGGCACCTA TCTCAGCGAT CTGTCTATTT CGTTCATCCA

3781 TAGTTGCCTG ACTCCCCGTC GTGTAGATAA CTACGATACG GGAGGGCTTA CCATCTGGCC

3841 CCAGCGCTGC GATGATACCG CGAGAACCAC GCTCACCGGC TCCGGATTTA TCAGCAATAA

3901 ACCAGCCAGC CGGAAGGGCC GAGCGCAGAA GTGGTCCTGC AACTTTATCC GCCTCCATCC

3961 AGTCTATTAA TTGTTGCCGG GAAGCTAGAG TAAGTAGTTC GCCAGTTAAT AGTTTGCGCA

4021 ACGTTGTTGC CATCGCTACA GGCATCGTGG TGTCACGCTC GTCGTTTGGT ATGGCTTCAT

4081 TCAGCTCCGG TTCCCAACGA TCAAGGCGAG TTACATGATC CCCCATGTTG TGCAAAAAAG

4141 CGGTTAGCTC CTTCGGTCCT CCGATCGTTG TCAGAAGTAA GTTGGCCGCA GTGTTATCAC

4201 TCATGGTTAT GGCAGCACTG CATAATTCTC TTACTGTCAT GCCATCCGTA AGATGCTTTT

4261 CTGTGACTGG TGAGTACTCA ACCAAGTCAT TCTGAGAATA GTGTATGCGG CGACCGAGTT

4321 GCTCTTGCCC GGCGTCAATA CGGGATAATA CCGCGCCACA TAGCAGAACT TTAAAAGTGC

4381 TCATCATTGG AAAACGTTCT TCGGGGCGAA AACTCTCAAG GATCTTACCG CTGTTGAGAT

4441 CCAGTTCGAT GTAACCCACT CGTGCACCCA ACTGATCTTC AGCATCTTTT ACTTTCACCA

4501 GCGTTTCTGG GTGAGCAAAA ACAGGAAGGC AAAATGCCGC AAAAAAGGGA ATAAGGGCGA

4561 CACGGAAATG TTGAATACTC ATATTCTTCC TTTTTCAATA TTATTGAAGC ATTTATCAGG

4621 GTTATTGTCT CATGAGCGGA TACATATTTG AATGTATTTA GAAAAATAAA CAAATAGGGG

4681 TCAGTGTTAC AACCAATTAA CCAATTCTGA ACATTATCGC GAGCCCATTT ATACCTGAAT

4741 ATGGCTCATA ACACCCCTTG TTTGCCTGGC GGCAGTAGCG CGGTGGTCCC ACCTGACCCC

4801 ATGCCGAACT CAGAAGTGAA ACGCCGTAGC GCCGATGGTA GTGTGGGGAC TCCCCATGCG

4861 AGAGTAGGGA ACTGCCAGGC ATCAAATAAA ACGAAAGGCT CAGTCGAAAG ACTGGGCCTT

4921 TCGCCCGGGC TAATTATGGG GTGTCGCCCT TATTCGACTC TATAGTGAAG TTCCTATTCT

4981 CTAGAAAGTA TAGGAACTTC TGAAGTGGGG ATTTAAATGC GGCCGCGCTG AGGGTTTAAT

5041 GGCGCGCCGC GGCCGCCCGC GGTGTTGGAA CGAGAGTAAT TAATAGTGAC ATGAGTTGCT

5101 ATGGTAACAA TCTAATGCTT ACATCGTATA TTAATGTACA ACTCGTATAC GTTTAAGTGT

5161 GATTGCGCCT ATTGCAGAAG GAATGTTAAA CGAGAAGCTC AGACAATACT GAAGCTGTGT

5221 TAAAGACCTA TTAGTTGAAC ATGTTATGCT AGCATTAAGT CCTCAGCGAG CTCGCATGGA

5281 ATGCGTGCGA TGAGCGACCT CATGCTATAC CTGAGAAAGC AACCTGACCT ACAGGAAAGA

5341 GTTACTCAAG AATAAGAATT TTCGTTTTAA AACCTAAGAG TCACTTTAAA ATTTGTATAC

5401 ACTTATTTTT TTTATAACTT ATTTAATAAT AAAAATCATA AATCATAAGA AATTCGCTTA

5461 TTTAGAAGTG TCAACAACGT ATCTACCAAC GGAATG

**pCfB2992_(pTY1Cons1-KlLEU2-deg) 6288 bp DNA circular UNA**

FEATURES Location/Qualifiers

Misc._feature 1..14

/label="USER cassette"

Terminator 20..190

/label="T CYC1"

Misc._feature 257..316

/label=loxP

Terminator complement(317..439)

/label="T URA3"

Misc._structure complement(440..490)

/label="Degradation tag"

CDS complement(491..1576)

/label="KlLEU2syn without STOP codon"

Promoter complement(1577..2606)

/label="P LEU2"

Misc._feature 2623..2656

/label=loxP

Misc._feature 2796..3003

/label="TY1 C1 3'"

/label="Consensus TY1Cons1 3'"

Replication_ori complement(3776..4419)

/label="pUC ori"

CDS complement(4522..5380)

/label=AmpR

Misc._feature 5824..6057

/label="TY1 C1 5'"

/label="Consensus TY1Cons1 5'"

Terminator complement(6084..6278)

/label="T ADH1"

ORIGIN

1 CGTGCGATCG CGTGCATTCA TCCGCTCTAA CCGAAAAGGA AGGAGTTAGA CAACCTGAAG

61 TCTAGGTCCC TATTTATTTT TTTATAGTTA TGTTAGTATT AAGAACGTTA TTTATATTTC

121 AAATTTTTCT TTTTTTTCTG TACAGACGCG TGTACGCATG TAACATTATA CTGAAAACCT

181 TGCTTGAGAA GGTTTTGGGA CGCTCGAAGA TCGCGTCAGC TGAAGCTTCG TACGCTGCAG

241 GTCGACAACC CTTAATGTCG ACAACCCTTA ATATAACTTC GTATAATGTA TGCTATACGA

301 AGTTATTAGG TCTAGAGATC CCAATACAAC AGATCACGTG ATCTTTTGTA AGATGAAGTT

361 GAAGTGAGTG TTGCACCGTG CCAATGCAGG TGGCTATTAG ATTAAATATG TGATTTGTTC

421 TATTAAGTTT CCTGTATAAT TACAAATGAA TAACGAAATG AGACAAAGAA GAGAACCAAU

481 TTTTACAAGC AGCCAAGATT TCCTTGACAG CCTTGGCGAT AGCATCGCCA ACCTCAGTGG

541 TAGAGTTAGA ACCACCAAGG TCACCGGTTC TGACACCTGC ATCCAAGACA TTTCTAACAG

601 CTTCTTCAAG AGCCCTACCT TCTTCAACCA AATCCAAGGA TAACTTCAAC ATCATAGCTG

661 CAGATAAGAT GGTAGCAATT GGGTTAACCT TGTTTGCTGG TAAATCTGGG GCAGAACCAT

721 GACATGGTTC GTACAAACCG AATGCCTTGT TAGTGTCAGG TAGGGAAGCT AGAGATGCAG

781 AAGGTAATAA ACCCAAAGAA CCTGGAATAA CAGAGGCTTC ATCGGAGATA ATATCACCAA

841 ACATGTTGTT GGTAATAACA ACACCGTTTA GCTTAGTTGG TGATTTAACC AAAATCATAG

901 CAGCAGAGTC GATCAATTGG TGCTGAACAG TTAATTGTGG GAACTCAGTC TTGATGGTTT

961 CTTCAACAGT CTTTCTCCAC AATCTGGAAG AGGCAAGCAC GTTAGCCTTG TCAAGTGACC

1021 AGATTGGTAA TGGTGGGTTT TGTTGCAATG CCAAGAAAGC AGCCATTCTT GTAATTCTTT

1081 GAACTTCAGG AACACTGTAT TTCTCAGAGT CCCAAGCAAC TCCGTCACCT TCATCTTCTT

1141 TTCTTTCACC AAAGTAGATA CCACCAACCA ATTCTCTAAC GACGACGAAA TCGGTACCCT

1201 TTGCATATTC AGGCTTCAAA GGAGAAAGAT CTAGTAAAGA ATCAGAAGCA AAGTTACATG

1261 GTCTTAAGTT GGCGTATAGA CCCAATTCCT TTCTGATCTT CAATAGACCT TGTTCTGGTC

1321 TAACTGCGCC CGTACCCCAT TTTGGACCAC CAACAGCACC TAGTAAGACA GCATCGGCTT

1381 TCTTAGAGGC TTCTAGAGCT TCATCTGGTA AAGGAGTGCC AGTGGCATCG ATGGCAGCAC

1441 CCCCGATCAA GTGATGTTGG AAATTGAACT TAATTTCTGG ACGGACTTCA GCAATGGCAT

1501 TCAAGACCTT AATAGCTTCG TCAGTAACTT CTTTACCGAC GTGATCACCC GGTAGGACAA

1561 CGATATTCTT AGACATGGTA TAATCTGTGT AGTGTGGGAT ACTTTTTACT TCTTCAAATA

1621 GGTATCAACA ATAAAAATAA ATCGAAGCAA ATGTAGGAAT GCGTTAAAGC AGATGTACTT

1681 ACTTAGAGTA CATAGATATA TTTATATAAT TCAATATATA AAAGTATATG AAGCATCTGA

1741 TGTTGAACCT GTCATGACTC TAAACTGGAA TGGGTAGTTA TGCTTCTGAA GGTTTTCCGT

1801 TAGAAAATGA GTGCAGGGAT CAGGCCCGGA ACCGGTTTTA GCCTGAAAGG AAAAGAAAAA

1861 AATGCGGTGA TTACTAGCAC GTGACTGCGC TGAATTGGAA TCCACCACAA GTACAGCGAA

1921 TGGCAAGAGG GAAGGGGAAT ATATTGTGCC TGGCGGTAGT CTGCTATATC AAATGTTAAA

1981 CATAACTAAA TGCAGTAGTG AATAGAGTAA TGTATTTCTA CATATGTGGG TAGGGCCAAT

2041 GGGAGCCCGA TGTCTATTGT ACAGATATTT TCCTTTTATA GTTAATCAAT TCAACTTGGA

2101 AATCATTGAG TCGATATCTA CTTGGATAAC TGGAGCCGTG GCAGCGTTTG AAATTTGACG

2161 AGTCTCCTTG TTTTGTTCGC GCATTTTCAG TTTATTCAAA CGGGAGATGG CACCGAATCC

2221 ACTGACTTCT CTGTTCAATT TGAATGGTAA TAGGAAGAAA GTACATTTCG AGAAATTTTC

2281 GCCCAACGTC GAAATCTCAT GGATGATATC CTCGATACAA ATGACACCTT CATCACCTAA

2341 TTTCTCTTCA ACGATATTGT TGTCATTCAA GATTATCTCT TTTGGTTCTT TATCTTCTGG

2401 CCTTTGCCAC ATTATTCTAG ATCTCTTTTG GATTAAAGAA CGGATAGATG CAAGTGAAGG

2461 TTGTCCGATA ACAATATATG GTGATACAAG TTTCAACAAT GGGTACACGT CCTTGGTAAG

2521 TTTGATGAAA ACACCAGTGT TCAACTCAAC AAGTCTCAAA AGAGATAACA CTTTGTAAGC

2581 CTTTGCTGGG ATCTTCACAG CGAGCTCTCG AGAACCCTTA ATATAACTTC GTATAATGTA

2641 TGCTATACGA AGTTATTAGG TGATATCAGA TCCACTAGTG GCCTATGCAC CCAATTCGCC

2701 CTATAGTGAG TCGTATTACG CGCGCTCACT GGCCGTCGTT TTACAACGTC GTGACTGGGA

2761 AAACCCTGGC GTTACCCCTG CAGGACAGAC GTCATTAGTG CTGAGGCATT AATTGATCAA

2821 AAACGGAATG AGGAATAATC GTAATATTAG TATGTAGAAA TATAGATTCC ATTTTGAGGA

2881 TTCCTATATC CTCGAGGAGA ACTTCTAGTG TATATTCTGT ATACCTAATA TTATAGCCTT

2941 TATCAACAAT GGAATCCCAA CAATTATCTA ATTACCCACA AATTTCTCAA GATCTGCGGC

3001 CGCACTCAGA CCTGAAGTGA AGTTCCTATA CTTTCTAGAG AATAGGAACT TCTATAGTGA

3061 GTCGAATAAG GGCGACACAA AATTTATTCT AAATGCATAA TAAATACTGA TAACATCTTA

3121 TAGTTTGTAT TATATTTTGT ATTATCGTTG ACATGTATAA TTTTGATATC AAAAACTGAT

3181 TTTCCCTTTA TTATTTTCGA GATTTATTTT CTTAATTCTC TTTAACAAAC TAGAAATATT

3241 GTATATACAA AAAATCATAA ATAATAGATG AATAGTTTAA TTATAGGTGT TCATCAATCG

3301 AAAAAGCAAC GTATCTTATT TAAAGTGCGT TGCTTTTTTC TCATTTATAA GGTTAAATAA

3361 TTCTCATATA TCAAGCAAAG TGACAGGCGC CCTTAAATAT TCTGACAAAT GCTCTTTCCC

3421 TAAACTCCCC CCATAAAAAA ACCCGCCGAA GCGGGTTTTT ACGTTATTTG CGGATTAACG

3481 ATTACTCGTT ATCAGAACCG CCCAGGGGGC CCGAGCTTAA GACTGGCCGT CGTTTTACAA

3541 CACAGAAAGA GTTTGTAGAA ACGCAAAAAG GCCATCCGTC AGGGGCCTTC TGCTTAGTTT

3601 GATGCCTGGC AGTTCCCTAC TCTCGCCTTC CGCTTCCTCG CTCACTGACT CGCTGCGCTC

3661 GGTCGTTCGG CTGCGGCGAG CGGTATCAGC TCACTCAAAG GCGGTAATAC GGTTATCCAC

3721 AGAATCAGGG GATAACGCAG GAAAGAACAT GTGAGCAAAA GGCCAGCAAA AGGCCAGGAA

3781 CCGTAAAAAG GCCGCGTTGC TGGCGTTTTT CCATAGGCTC CGCCCCCCTG ACGAGCATCA

3841 CAAAAATCGA CGCTCAAGTC AGAGGTGGCG AAACCCGACA GGACTATAAA GATACCAGGC

3901 GTTTCCCCCT GGAAGCTCCC TCGTGCGCTC TCCTGTTCCG ACCCTGCCGC TTACCGGATA

3961 CCTGTCCGCC TTTCTCCCTT CGGGAAGCGT GGCGCTTTCT CATAGCTCAC GCTGTAGGTA

4021 TCTCAGTTCG GTGTAGGTCG TTCGCTCCAA GCTGGGCTGT GTGCACGAAC CCCCCGTTCA

4081 GCCCGACCGC TGCGCCTTAT CCGGTAACTA TCGTCTTGAG TCCAACCCGG TAAGACACGA

4141 CTTATCGCCA CTGGCAGCAG CCACTGGTAA CAGGATTAGC AGAGCGAGGT ATGTAGGCGG

4201 TGCTACAGAG TTCTTGAAGT GGTGGGCTAA CTACGGCTAC ACTAGAAGAA CAGTATTTGG

4261 TATCTGCGCT CTGCTGAAGC CAGTTACCTT CGGAAAAAGA GTTGGTAGCT CTTGATCCGG

4321 CAAACAAACC ACCGCTGGTA GCGGTGGTTT TTTTGTTTGC AAGCAGCAGA TTACGCGCAG

4381 AAAAAAAGGA TCTCAAGAAG ATCCTTTGAT CTTTTCTACG GGGTCTGACG CTCAGTGGAA

4441 CGACGCGCGC GTAACTCACG TTAAGGGATT TTGGTCATGA GCTTGCGCCG TCCCGTCAAG

4501 TCAGCGTAAT GCTCTGCTTT TACCAATGCT TAATCAGTGA GGCACCTATC TCAGCGATCT

4561 GTCTATTTCG TTCATCCATA GTTGCCTGAC TCCCCGTCGT GTAGATAACT ACGATACGGG

4621 AGGGCTTACC ATCTGGCCCC AGCGCTGCGA TGATACCGCG AGAACCACGC TCACCGGCTC

4681 CGGATTTATC AGCAATAAAC CAGCCAGCCG GAAGGGCCGA GCGCAGAAGT GGTCCTGCAA

4741 CTTTATCCGC CTCCATCCAG TCTATTAATT GTTGCCGGGA AGCTAGAGTA AGTAGTTCGC

4801 CAGTTAATAG TTTGCGCAAC GTTGTTGCCA TCGCTACAGG CATCGTGGTG TCACGCTCGT

4861 CGTTTGGTAT GGCTTCATTC AGCTCCGGTT CCCAACGATC AAGGCGAGTT ACATGATCCC

4921 CCATGTTGTG CAAAAAAGCG GTTAGCTCCT TCGGTCCTCC GATCGTTGTC AGAAGTAAGT

4981 TGGCCGCAGT GTTATCACTC ATGGTTATGG CAGCACTGCA TAATTCTCTT ACTGTCATGC

5041 CATCCGTAAG ATGCTTTTCT GTGACTGGTG AGTACTCAAC CAAGTCATTC TGAGAATAGT

5101 GTATGCGGCG ACCGAGTTGC TCTTGCCCGG CGTCAATACG GGATAATACC GCGCCACATA

5161 GCAGAACTTT AAAAGTGCTC ATCATTGGAA AACGTTCTTC GGGGCGAAAA CTCTCAAGGA

5221 TCTTACCGCT GTTGAGATCC AGTTCGATGT AACCCACTCG TGCACCCAAC TGATCTTCAG

5281 CATCTTTTAC TTTCACCAGC GTTTCTGGGT GAGCAAAAAC AGGAAGGCAA AATGCCGCAA

5341 AAAAGGGAAT AAGGGCGACA CGGAAATGTT GAATACTCAT ATTCTTCCTT TTTCAATATT

5401 ATTGAAGCAT TTATCAGGGT TATTGTCTCA TGAGCGGATA CATATTTGAA TGTATTTAGA

5461 AAAATAAACA AATAGGGGTC AGTGTTACAA CCAATTAACC AATTCTGAAC ATTATCGCGA

5521 GCCCATTTAT ACCTGAATAT GGCTCATAAC ACCCCTTGTT TGCCTGGCGG CAGTAGCGCG

5581 GTGGTCCCAC CTGACCCCAT GCCGAACTCA GAAGTGAAAC GCCGTAGCGC CGATGGTAGT

5641 GTGGGGACTC CCCATGCGAG AGTAGGGAAC TGCCAGGCAT CAAATAAAAC GAAAGGCTCA

5701 GTCGAAAGAC TGGGCCTTTC GCCCGGGCTA ATTATGGGGT GTCGCCCTTA TTCGACTCTA

5761 TAGTGAAGTT CCTATTCTCT AGAAAGTATA GGAACTTCTG AAGTGGGGAT TTAAAGTCGG

5821 TGTCCGCGCT GAGGGTTTAA TGGCGCGCCG CGGCCGCCCG CGGTGTTGGA ATAAAAATCC

5881 ACTATCGTCT ATCAACTAAT AGTTATATTA TCAATATATT ATCATATACG GTGTTAAGAT

5941 GATGACATAA GTTATGAGAA GCTGTCATCG AAGTTAGAGG AAGCTGAAGT GCAAGGATTG

6001 ATAATGTAAT AGGATCAATG AATATAAACA TATGCTAGCA TTAAGTCCTC AGCGAGCACG

6061 CTGCTTCATG GAATGCGTGC GATGAGCGAC CTCATGCTAT ACCTGAGAAA GCAACCTGAC

6121 CTACAGGAAA GAGTTACTCA AGAATAAGAA TTTTCGTTTT AAAACCTAAG AGTCACTTTA

6181 AAATTTGTAT ACACTTATTT TTTTTATAAC TTATTTAATA ATAAAAATCA TAAATCATAA

6241 GAAATTCGCT TATTTAGAAG TGTCAACAAC GTATCTACCA ACGGAATG

**pCfB2991_(pTY1Cons2-KlLEU2-deg) 6285 bp DNA circular UNA**

FEATURES Location/Qualifiers

Misc._feature 1..14

/label="USER cassette"

Terminator 20..190

/label="T CYC1"

Misc._feature 257..316

/label=loxP

Terminator complement(317..439)

/label="T URA3"

Misc._structure complement(440..490)

/label="Degradation tag"

CDS complement(491..1576)

/label="KlLEU2syn without STOP codon"

Promoter complement(1577..2606)

/label="P LEU2"

Misc._feature 2623..2656

/label=loxP

Misc._feature 2796..3001

/label="TY1 C234 3'"

/label="Consensus TY1Cons2 3'"

Replication_ori complement(3775..4418)

/label="pUC ori"

CDS complement(4521..5379)

/label=AmpR

Misc._feature 5823..6054

/label="TY1 C234 5'"

/label="Consensus TY1Cons2 5'"

Terminator complement(6081..6275)

/label="T ADH1"

ORIGIN

1 CGTGCGATCG CGTGCATTCA TCCGCTCTAA CCGAAAAGGA AGGAGTTAGA CAACCTGAAG

61 TCTAGGTCCC TATTTATTTT TTTATAGTTA TGTTAGTATT AAGAACGTTA TTTATATTTC

121 AAATTTTTCT TTTTTTTCTG TACAGACGCG TGTACGCATG TAACATTATA CTGAAAACCT

181 TGCTTGAGAA GGTTTTGGGA CGCTCGAAGA TCGCGTCAGC TGAAGCTTCG TACGCTGCAG

241 GTCGACAACC CTTAATGTCG ACAACCCTTA ATATAACTTC GTATAATGTA TGCTATACGA

301 AGTTATTAGG TCTAGAGATC CCAATACAAC AGATCACGTG ATCTTTTGTA AGATGAAGTT

361 GAAGTGAGTG TTGCACCGTG CCAATGCAGG TGGCTATTAG ATTAAATATG TGATTTGTTC

421 TATTAAGTTT CCTGTATAAT TACAAATGAA TAACGAAATG AGACAAAGAA GAGAACCAAU

481 TTTTACAAGC AGCCAAGATT TCCTTGACAG CCTTGGCGAT AGCATCGCCA ACCTCAGTGG

541 TAGAGTTAGA ACCACCAAGG TCACCGGTTC TGACACCTGC ATCCAAGACA TTTCTAACAG

601 CTTCTTCAAG AGCCCTACCT TCTTCAACCA AATCCAAGGA TAACTTCAAC ATCATAGCTG

661 CAGATAAGAT GGTAGCAATT GGGTTAACCT TGTTTGCTGG TAAATCTGGG GCAGAACCAT

721 GACATGGTTC GTACAAACCG AATGCCTTGT TAGTGTCAGG TAGGGAAGCT AGAGATGCAG

781 AAGGTAATAA ACCCAAAGAA CCTGGAATAA CAGAGGCTTC ATCGGAGATA ATATCACCAA

841 ACATGTTGTT GGTAATAACA ACACCGTTTA GCTTAGTTGG TGATTTAACC AAAATCATAG

901 CAGCAGAGTC GATCAATTGG TGCTGAACAG TTAATTGTGG GAACTCAGTC TTGATGGTTT

961 CTTCAACAGT CTTTCTCCAC AATCTGGAAG AGGCAAGCAC GTTAGCCTTG TCAAGTGACC

1021 AGATTGGTAA TGGTGGGTTT TGTTGCAATG CCAAGAAAGC AGCCATTCTT GTAATTCTTT

1081 GAACTTCAGG AACACTGTAT TTCTCAGAGT CCCAAGCAAC TCCGTCACCT TCATCTTCTT

1141 TTCTTTCACC AAAGTAGATA CCACCAACCA ATTCTCTAAC GACGACGAAA TCGGTACCCT

1201 TTGCATATTC AGGCTTCAAA GGAGAAAGAT CTAGTAAAGA ATCAGAAGCA AAGTTACATG

1261 GTCTTAAGTT GGCGTATAGA CCCAATTCCT TTCTGATCTT CAATAGACCT TGTTCTGGTC

1321 TAACTGCGCC CGTACCCCAT TTTGGACCAC CAACAGCACC TAGTAAGACA GCATCGGCTT

1381 TCTTAGAGGC TTCTAGAGCT TCATCTGGTA AAGGAGTGCC AGTGGCATCG ATGGCAGCAC

1441 CCCCGATCAA GTGATGTTGG AAATTGAACT TAATTTCTGG ACGGACTTCA GCAATGGCAT

1501 TCAAGACCTT AATAGCTTCG TCAGTAACTT CTTTACCGAC GTGATCACCC GGTAGGACAA

1561 CGATATTCTT AGACATGGTA TAATCTGTGT AGTGTGGGAT ACTTTTTACT TCTTCAAATA

1621 GGTATCAACA ATAAAAATAA ATCGAAGCAA ATGTAGGAAT GCGTTAAAGC AGATGTACTT

1681 ACTTAGAGTA CATAGATATA TTTATATAAT TCAATATATA AAAGTATATG AAGCATCTGA

1741 TGTTGAACCT GTCATGACTC TAAACTGGAA TGGGTAGTTA TGCTTCTGAA GGTTTTCCGT

1801 TAGAAAATGA GTGCAGGGAT CAGGCCCGGA ACCGGTTTTA GCCTGAAAGG AAAAGAAAAA

1861 AATGCGGTGA TTACTAGCAC GTGACTGCGC TGAATTGGAA TCCACCACAA GTACAGCGAA

1921 TGGCAAGAGG GAAGGGGAAT ATATTGTGCC TGGCGGTAGT CTGCTATATC AAATGTTAAA

1981 CATAACTAAA TGCAGTAGTG AATAGAGTAA TGTATTTCTA CATATGTGGG TAGGGCCAAT

2041 GGGAGCCCGA TGTCTATTGT ACAGATATTT TCCTTTTATA GTTAATCAAT TCAACTTGGA

2101 AATCATTGAG TCGATATCTA CTTGGATAAC TGGAGCCGTG GCAGCGTTTG AAATTTGACG

2161 AGTCTCCTTG TTTTGTTCGC GCATTTTCAG TTTATTCAAA CGGGAGATGG CACCGAATCC

2221 ACTGACTTCT CTGTTCAATT TGAATGGTAA TAGGAAGAAA GTACATTTCG AGAAATTTTC

2281 GCCCAACGTC GAAATCTCAT GGATGATATC CTCGATACAA ATGACACCTT CATCACCTAA

2341 TTTCTCTTCA ACGATATTGT TGTCATTCAA GATTATCTCT TTTGGTTCTT TATCTTCTGG

2401 CCTTTGCCAC ATTATTCTAG ATCTCTTTTG GATTAAAGAA CGGATAGATG CAAGTGAAGG

2461 TTGTCCGATA ACAATATATG GTGATACAAG TTTCAACAAT GGGTACACGT CCTTGGTAAG

2521 TTTGATGAAA ACACCAGTGT TCAACTCAAC AAGTCTCAAA AGAGATAACA CTTTGTAAGC

2581 CTTTGCTGGG ATCTTCACAG CGAGCTCTCG AGAACCCTTA ATATAACTTC GTATAATGTA

2641 TGCTATACGA AGTTATTAGG TGATATCAGA TCCACTAGTG GCCTATGCAC CCAATTCGCC

2701 CTATAGTGAG TCGTATTACG CGCGCTCACT GGCCGTCGTT TTACAACGTC GTGACTGGGA

2761 AAACCCTGGC GTTACCCCTG CAGGACAGAC GTCATTAGTG CTGAGGCATT AATTGATCAT

2821 AAAACGGAAT GATGAATAAT ATTTATAGAA TTGTGTAGAA TTGCAGATTC CCTTTTATGG

2881 ATTCCTAAAT CCTCGAGGAG AACTTCTAGT ATATTCTGTA TACCTAATAT TATAGCCTTT

2941 ATCAACAATG GAATCCCAAC AATTATCTCA AAATTCACAT ATTTCTCAAG ATCTGCGGCC

3001 GCACTCAGAC CTGAAGTGAA GTTCCTATAC TTTCTAGAGA ATAGGAACTT CTATAGTGAG

3061 TCGAATAAGG GCGACACAAA ATTTATTCTA AATGCATAAT AAATACTGAT AACATCTTAT

3121 AGTTTGTATT ATATTTTGTA TTATCGTTGA CATGTATAAT TTTGATATCA AAAACTGATT

3181 TTCCCTTTAT TATTTTCGAG ATTTATTTTC TTAATTCTCT TTAACAAACT AGAAATATTG

3241 TATATACAAA AAATCATAAA TAATAGATGA ATAGTTTAAT TATAGGTGTT CATCAATCGA

3301 AAAAGCAACG TATCTTATTT AAAGTGCGTT GCTTTTTTCT CATTTATAAG GTTAAATAAT

3361 TCTCATATAT CAAGCAAAGT GACAGGCGCC CTTAAATATT CTGACAAATG CTCTTTCCCT

3421 AAACTCCCCC CATAAAAAAA CCCGCCGAAG CGGGTTTTTA CGTTATTTGC GGATTAACGA

3481 TTACTCGTTA TCAGAACCGC CCAGGGGGCC CGAGCTTAAG ACTGGCCGTC GTTTTACAAC

3541 ACAGAAAGAG TTTGTAGAAA CGCAAAAAGG CCATCCGTCA GGGGCCTTCT GCTTAGTTTG

3601 ATGCCTGGCA GTTCCCTACT CTCGCCTTCC GCTTCCTCGC TCACTGACTC GCTGCGCTCG

3661 GTCGTTCGGC TGCGGCGAGC GGTATCAGCT CACTCAAAGG CGGTAATACG GTTATCCACA

3721 GAATCAGGGG ATAACGCAGG AAAGAACATG TGAGCAAAAG GCCAGCAAAA GGCCAGGAAC

3781 CGTAAAAAGG CCGCGTTGCT GGCGTTTTTC CATAGGCTCC GCCCCCCTGA CGAGCATCAC

3841 AAAAATCGAC GCTCAAGTCA GAGGTGGCGA AACCCGACAG GACTATAAAG ATACCAGGCG

3901 TTTCCCCCTG GAAGCTCCCT CGTGCGCTCT CCTGTTCCGA CCCTGCCGCT TACCGGATAC

3961 CTGTCCGCCT TTCTCCCTTC GGGAAGCGTG GCGCTTTCTC ATAGCTCACG CTGTAGGTAT

4021 CTCAGTTCGG TGTAGGTCGT TCGCTCCAAG CTGGGCTGTG TGCACGAACC CCCCGTTCAG

4081 CCCGACCGCT GCGCCTTATC CGGTAACTAT CGTCTTGAGT CCAACCCGGT AAGACACGAC

4141 TTATCGCCAC TGGCAGCAGC CACTGGTAAC AGGATTAGCA GAGCGAGGTA TGTAGGCGGT

4201 GCTACAGAGT TCTTGAAGTG GTGGGCTAAC TACGGCTACA CTAGAAGAAC AGTATTTGGT

4261 ATCTGCGCTC TGCTGAAGCC AGTTACCTTC GGAAAAAGAG TTGGTAGCTC TTGATCCGGC

4321 AAACAAACCA CCGCTGGTAG CGGTGGTTTT TTTGTTTGCA AGCAGCAGAT TACGCGCAGA

4381 AAAAAAGGAT CTCAAGAAGA TCCTTTGATC TTTTCTACGG GGTCTGACGC TCAGTGGAAC

4441 GACGCGCGCG TAACTCACGT TAAGGGATTT TGGTCATGAG CTTGCGCCGT CCCGTCAAGT

4501 CAGCGTAATG CTCTGCTTTT ACCAATGCTT AATCAGTGAG GCACCTATCT CAGCGATCTG

4561 TCTATTTCGT TCATCCATAG TTGCCTGACT CCCCGTCGTG TAGATAACTA CGATACGGGA

4621 GGGCTTACCA TCTGGCCCCA GCGCTGCGAT GATACCGCGA GAACCACGCT CACCGGCTCC

4681 GGATTTATCA GCAATAAACC AGCCAGCCGG AAGGGCCGAG CGCAGAAGTG GTCCTGCAAC

4741 TTTATCCGCC TCCATCCAGT CTATTAATTG TTGCCGGGAA GCTAGAGTAA GTAGTTCGCC

4801 AGTTAATAGT TTGCGCAACG TTGTTGCCAT CGCTACAGGC ATCGTGGTGT CACGCTCGTC

4861 GTTTGGTATG GCTTCATTCA GCTCCGGTTC CCAACGATCA AGGCGAGTTA CATGATCCCC

4921 CATGTTGTGC AAAAAAGCGG TTAGCTCCTT CGGTCCTCCG ATCGTTGTCA GAAGTAAGTT

4981 GGCCGCAGTG TTATCACTCA TGGTTATGGC AGCACTGCAT AATTCTCTTA CTGTCATGCC

5041 ATCCGTAAGA TGCTTTTCTG TGACTGGTGA GTACTCAACC AAGTCATTCT GAGAATAGTG

5101 TATGCGGCGA CCGAGTTGCT CTTGCCCGGC GTCAATACGG GATAATACCG CGCCACATAG

5161 CAGAACTTTA AAAGTGCTCA TCATTGGAAA ACGTTCTTCG GGGCGAAAAC TCTCAAGGAT

5221 CTTACCGCTG TTGAGATCCA GTTCGATGTA ACCCACTCGT GCACCCAACT GATCTTCAGC

5281 ATCTTTTACT TTCACCAGCG TTTCTGGGTG AGCAAAAACA GGAAGGCAAA ATGCCGCAAA

5341 AAAGGGAATA AGGGCGACAC GGAAATGTTG AATACTCATA TTCTTCCTTT TTCAATATTA

5401 TTGAAGCATT TATCAGGGTT ATTGTCTCAT GAGCGGATAC ATATTTGAAT GTATTTAGAA

5461 AAATAAACAA ATAGGGGTCA GTGTTACAAC CAATTAACCA ATTCTGAACA TTATCGCGAG

5521 CCCATTTATA CCTGAATATG GCTCATAACA CCCCTTGTTT GCCTGGCGGC AGTAGCGCGG

5581 TGGTCCCACC TGACCCCATG CCGAACTCAG AAGTGAAACG CCGTAGCGCC GATGGTAGTG

5641 TGGGGACTCC CCATGCGAGA GTAGGGAACT GCCAGGCATC AAATAAAACG AAAGGCTCAG

5701 TCGAAAGACT GGGCCTTTCG CCCGGGCTAA TTATGGGGTG TCGCCCTTAT TCGACTCTAT

5761 AGTGAAGTTC CTATTCTCTA GAAAGTATAG GAACTTCTGA AGTGGGGATT TAAAGTCGGT

5821 GTCCGCGCTG AGGGTTTAAT GGCGCGCCGC GGCCGCCCGC GGTGTTGGAA TAAAAATCAA

5881 CTATCATCTA CTAACTAGTA TTTACGTTAC TAGTATATTA TCATATACGG TGTTAGAAGA

5941 TGACGCAAAT GATGAGAAAT AGTCATCTAA ATTAGTGGAA GCTGAAACGC AAGGATTGAT

6001 AATGTAATAG GATCAATGAA TATTAACATA GCTAGCATTA AGTCCTCAGC GAGCACGCTG

6061 CTTCATGGAA TGCGTGCGAT GAGCGACCTC ATGCTATACC TGAGAAAGCA ACCTGACCTA

6121 CAGGAAAGAG TTACTCAAGA ATAAGAATTT TCGTTTTAAA ACCTAAGAGT CACTTTAAAA

6181 TTTGTATACA CTTATTTTTT TTATAACTTA TTTAATAATA AAAATCATAA ATCATAAGAA

6241 ATTCGCTTAT TTAGAAGTGT CAACAACGTA TCTACCAACG GAATG

**pCfB2804_(pTY2-KlLEU2-deg) 6257 bp DNA circular UNA**

FEATURES Location/Qualifiers

Misc._feature 1..14

/label="USER cassette"

Terminator 20..190

/label="T CYC1"

Misc._feature 238..297

/label=loxP

Terminator complement(298..420)

/label="T URA3"

Misc._structure complement(421..471)

/label="Degradation tag"

CDS complement(472..1557)

/label="KlLEU2syn without STOP codon"

Promoter complement(1558..2587)

/label="P LEU2"

Misc._feature 2604..2637

/label=loxP

Misc._feature 2777..2963

/label="TY2 3'"

/label="Consensus TY2 3'"

Replication_ori complement(3750..4393)

/label="pUC ori"

CDS complement(4496..5354)

/label=AmpR

Misc._feature 5798..6026

/label="TY2 5'"

/label="Consensus TY2 5'"

Terminator complement(6053..6247)

/label="T ADH1"

ORIGIN

1 CGTGCGATCG CGTGCATTCA TCCGCTCTAA CCGAAAAGGA AGGAGTTAGA CAACCTGAAG

61 TCTAGGTCCC TATTTATTTT TTTATAGTTA TGTTAGTATT AAGAACGTTA TTTATATTTC

121 AAATTTTTCT TTTTTTTCTG TACAGACGCG TGTACGCATG TAACATTATA CTGAAAACCT

181 TGCTTGAGAA ATCGCGTCAG CTGAAGCTTC GTACGCTGCA GGTCGACAAC CCTTAATGTC

241 GACAACCCTT AATATAACTT CGTATAATGT ATGCTATACG AAGTTATTAG GTCTAGAGAT

301 CCCAATACAA CAGATCACGT GATCTTTTGT AAGATGAAGT TGAAGTGAGT GTTGCACCGT

361 GCCAATGCAG GTGGCTATTA GATTAAATAT GTGATTTGTT CTATTAAGTT TCCTGTATAA

421 TTACAAATGA ATAACGAAAT GAGACAAAGA AGAGAACCAA UTTTTACAAG CAGCCAAGAT

481 TTCCTTGACA GCCTTGGCGA TAGCATCGCC AACCTCAGTG GTAGAGTTAG AACCACCAAG

541 GTCACCGGTT CTGACACCTG CATCCAAGAC ATTTCTAACA GCTTCTTCAA GAGCCCTACC

601 TTCTTCAACC AAATCCAAGG ATAACTTCAA CATCATAGCT GCAGATAAGA TGGTAGCAAT

661 TGGGTTAACC TTGTTTGCTG GTAAATCTGG GGCAGAACCA TGACATGGTT CGTACAAACC

721 GAATGCCTTG TTAGTGTCAG GTAGGGAAGC TAGAGATGCA GAAGGTAATA AACCCAAAGA

781 ACCTGGAATA ACAGAGGCTT CATCGGAGAT AATATCACCA AACATGTTGT TGGTAATAAC

841 AACACCGTTT AGCTTAGTTG GTGATTTAAC CAAAATCATA GCAGCAGAGT CGATCAATTG

901 GTGCTGAACA GTTAATTGTG GGAACTCAGT CTTGATGGTT TCTTCAACAG TCTTTCTCCA

961 CAATCTGGAA GAGGCAAGCA CGTTAGCCTT GTCAAGTGAC CAGATTGGTA ATGGTGGGTT

1021 TTGTTGCAAT GCCAAGAAAG CAGCCATTCT TGTAATTCTT TGAACTTCAG GAACACTGTA

1081 TTTCTCAGAG TCCCAAGCAA CTCCGTCACC TTCATCTTCT TTTCTTTCAC CAAAGTAGAT

1141 ACCACCAACC AATTCTCTAA CGACGACGAA ATCGGTACCC TTTGCATATT CAGGCTTCAA

1201 AGGAGAAAGA TCTAGTAAAG AATCAGAAGC AAAGTTACAT GGTCTTAAGT TGGCGTATAG

1261 ACCCAATTCC TTTCTGATCT TCAATAGACC TTGTTCTGGT CTAACTGCGC CCGTACCCCA

1321 TTTTGGACCA CCAACAGCAC CTAGTAAGAC AGCATCGGCT TTCTTAGAGG CTTCTAGAGC

1381 TTCATCTGGT AAAGGAGTGC CAGTGGCATC GATGGCAGCA CCCCCGATCA AGTGATGTTG

1441 GAAATTGAAC TTAATTTCTG GACGGACTTC AGCAATGGCA TTCAAGACCT TAATAGCTTC

1501 GTCAGTAACT TCTTTACCGA CGTGATCACC CGGTAGGACA ACGATATTCT TAGACATGGT

1561 ATAATCTGTG TAGTGTGGGA TACTTTTTAC TTCTTCAAAT AGGTATCAAC AATAAAAATA

1621 AATCGAAGCA AATGTAGGAA TGCGTTAAAG CAGATGTACT TACTTAGAGT ACATAGATAT

1681 ATTTATATAA TTCAATATAT AAAAGTATAT GAAGCATCTG ATGTTGAACC TGTCATGACT

1741 CTAAACTGGA ATGGGTAGTT ATGCTTCTGA AGGTTTTCCG TTAGAAAATG AGTGCAGGGA

1801 TCAGGCCCGG AACCGGTTTT AGCCTGAAAG GAAAAGAAAA AAATGCGGTG ATTACTAGCA

1861 CGTGACTGCG CTGAATTGGA ATCCACCACA AGTACAGCGA ATGGCAAGAG GGAAGGGGAA

1921 TATATTGTGC CTGGCGGTAG TCTGCTATAT CAAATGTTAA ACATAACTAA ATGCAGTAGT

1981 GAATAGAGTA ATGTATTTCT ACATATGTGG GTAGGGCCAA TGGGAGCCCG ATGTCTATTG

2041 TACAGATATT TTCCTTTTAT AGTTAATCAA TTCAACTTGG AAATCATTGA GTCGATATCT

2101 ACTTGGATAA CTGGAGCCGT GGCAGCGTTT GAAATTTGAC GAGTCTCCTT GTTTTGTTCG

2161 CGCATTTTCA GTTTATTCAA ACGGGAGATG GCACCGAATC CACTGACTTC TCTGTTCAAT

2221 TTGAATGGTA ATAGGAAGAA AGTACATTTC GAGAAATTTT CGCCCAACGT CGAAATCTCA

2281 TGGATGATAT CCTCGATACA AATGACACCT TCATCACCTA ATTTCTCTTC AACGATATTG

2341 TTGTCATTCA AGATTATCTC TTTTGGTTCT TTATCTTCTG GCCTTTGCCA CATTATTCTA

2401 GATCTCTTTT GGATTAAAGA ACGGATAGAT GCAAGTGAAG GTTGTCCGAT AACAATATAT

2461 GGTGATACAA GTTTCAACAA TGGGTACACG TCCTTGGTAA GTTTGATGAA AACACCAGTG

2521 TTCAACTCAA CAAGTCTCAA AAGAGATAAC ACTTTGTAAG CCTTTGCTGG GATCTTCACA

2581 GCGAGCTCTC GAGAACCCTT AATATAACTT CGTATAATGT ATGCTATACG AAGTTATTAG

2641 GTGATATCAG ATCCACTAGT GGCCTATGCA CCCAATTCGC CCTATAGTGA GTCGTATTAC

2701 GCGCGCTCAC TGGCCGTCGT TTTACAACGT CGTGACTGGG AAAACCCTGG CGTTACCCCT

2761 GCAGGACAGA CGTCATTAGT GCTGAGGCAT TAATTGATCA ATATAAAATG ATGATAATAA

2821 TATTTATAGA ATTGTGTAGA ATTGCAGATT CCCTTTTATG GATTCCTAAA TCCTGAGGAG

2881 AACTTCTAGT ATATTCTACA TACCTAATAT TATTGCCTTA TTAAAAATGG AATCCCAACA

2941 ATTACATCAA AATCCACATT CTCAGATCTG CGGCCGCACT CAGACCTGAA GTGAAGTTCC

3001 TATACTTTCT AGAGAATAGG AACTTCTATA GTGAGTCGAA TAAGGGCGAC ACAAAATTTA

3061 TTCTAAATGC ATAATAAATA CTGATAACAT CTTATAGTTT GTATTATATT TTGTATTATC

3121 GTTGACATGT ATAATTTTGA TATCAAAAAC TGATTTTCCC TTTATTATTT TCGAGATTTA

3181 TTTTCTTAAT TCTCTTTAAC AAACTAGAAA TATTGTATAT ACAAAAAATC ATAAATAATA

3241 GATGAATAGT TTAATTATAG GTGTTCATCA ATCGAAAAAG CAACGTATCT TATTTAAAGT

3301 GCGTTGCTTT TTTCTCATTT ATAAGGTTAA ATAATTCTCA TATATCAAGC AAAGTGACAG

3361 GCGCCCTTAA ATATTCTGAC AAATGCTCTT TCCCTAAACT CCCCCCATAA AAAAACCCGC

3421 CGAAGCGGGT TTTTACGTTA TTTGCGGATT AACGATTACT CGTTATCAGA ACCGCCCAGG

3481 GGGCCCGAGC TTAAGACTGG CCGTCGTTTT ACAACACAGA AAGAGTTTGT AGAAACGCAA

3541 AAAGGCCATC CGTCAGGGGC CTTCTGCTTA GTTTGATGCC TGGCAGTTCC CTACTCTCGC

3601 CTTCCGCTTC CTCGCTCACT GACTCGCTGC GCTCGGTCGT TCGGCTGCGG CGAGCGGTAT

3661 CAGCTCACTC AAAGGCGGTA ATACGGTTAT CCACAGAATC AGGGGATAAC GCAGGAAAGA

3721 ACATGTGAGC AAAAGGCCAG CAAAAGGCCA GGAACCGTAA AAAGGCCGCG TTGCTGGCGT

3781 TTTTCCATAG GCTCCGCCCC CCTGACGAGC ATCACAAAAA TCGACGCTCA AGTCAGAGGT

3841 GGCGAAACCC GACAGGACTA TAAAGATACC AGGCGTTTCC CCCTGGAAGC TCCCTCGTGC

3901 GCTCTCCTGT TCCGACCCTG CCGCTTACCG GATACCTGTC CGCCTTTCTC CCTTCGGGAA

3961 GCGTGGCGCT TTCTCATAGC TCACGCTGTA GGTATCTCAG TTCGGTGTAG GTCGTTCGCT

4021 CCAAGCTGGG CTGTGTGCAC GAACCCCCCG TTCAGCCCGA CCGCTGCGCC TTATCCGGTA

4081 ACTATCGTCT TGAGTCCAAC CCGGTAAGAC ACGACTTATC GCCACTGGCA GCAGCCACTG

4141 GTAACAGGAT TAGCAGAGCG AGGTATGTAG GCGGTGCTAC AGAGTTCTTG AAGTGGTGGG

4201 CTAACTACGG CTACACTAGA AGAACAGTAT TTGGTATCTG CGCTCTGCTG AAGCCAGTTA

4261 CCTTCGGAAA AAGAGTTGGT AGCTCTTGAT CCGGCAAACA AACCACCGCT GGTAGCGGTG

4321 GTTTTTTTGT TTGCAAGCAG CAGATTACGC GCAGAAAAAA AGGATCTCAA GAAGATCCTT

4381 TGATCTTTTC TACGGGGTCT GACGCTCAGT GGAACGACGC GCGCGTAACT CACGTTAAGG

4441 GATTTTGGTC ATGAGCTTGC GCCGTCCCGT CAAGTCAGCG TAATGCTCTG CTTTTACCAA

4501 TGCTTAATCA GTGAGGCACC TATCTCAGCG ATCTGTCTAT TTCGTTCATC CATAGTTGCC

4561 TGACTCCCCG TCGTGTAGAT AACTACGATA CGGGAGGGCT TACCATCTGG CCCCAGCGCT

4621 GCGATGATAC CGCGAGAACC ACGCTCACCG GCTCCGGATT TATCAGCAAT AAACCAGCCA

4681 GCCGGAAGGG CCGAGCGCAG AAGTGGTCCT GCAACTTTAT CCGCCTCCAT CCAGTCTATT

4741 AATTGTTGCC GGGAAGCTAG AGTAAGTAGT TCGCCAGTTA ATAGTTTGCG CAACGTTGTT

4801 GCCATCGCTA CAGGCATCGT GGTGTCACGC TCGTCGTTTG GTATGGCTTC ATTCAGCTCC

4861 GGTTCCCAAC GATCAAGGCG AGTTACATGA TCCCCCATGT TGTGCAAAAA AGCGGTTAGC

4921 TCCTTCGGTC CTCCGATCGT TGTCAGAAGT AAGTTGGCCG CAGTGTTATC ACTCATGGTT

4981 ATGGCAGCAC TGCATAATTC TCTTACTGTC ATGCCATCCG TAAGATGCTT TTCTGTGACT

5041 GGTGAGTACT CAACCAAGTC ATTCTGAGAA TAGTGTATGC GGCGACCGAG TTGCTCTTGC

5101 CCGGCGTCAA TACGGGATAA TACCGCGCCA CATAGCAGAA CTTTAAAAGT GCTCATCATT

5161 GGAAAACGTT CTTCGGGGCG AAAACTCTCA AGGATCTTAC CGCTGTTGAG ATCCAGTTCG

5221 ATGTAACCCA CTCGTGCACC CAACTGATCT TCAGCATCTT TTACTTTCAC CAGCGTTTCT

5281 GGGTGAGCAA AAACAGGAAG GCAAAATGCC GCAAAAAAGG GAATAAGGGC GACACGGAAA

5341 TGTTGAATAC TCATATTCTT CCTTTTTCAA TATTATTGAA GCATTTATCA GGGTTATTGT

5401 CTCATGAGCG GATACATATT TGAATGTATT TAGAAAAATA AACAAATAGG GGTCAGTGTT

5461 ACAACCAATT AACCAATTCT GAACATTATC GCGAGCCCAT TTATACCTGA ATATGGCTCA

5521 TAACACCCCT TGTTTGCCTG GCGGCAGTAG CGCGGTGGTC CCACCTGACC CCATGCCGAA

5581 CTCAGAAGTG AAACGCCGTA GCGCCGATGG TAGTGTGGGG ACTCCCCATG CGAGAGTAGG

5641 GAACTGCCAG GCATCAAATA AAACGAAAGG CTCAGTCGAA AGACTGGGCC TTTCGCCCGG

5701 GCTAATTATG GGGTGTCGCC CTTATTCGAC TCTATAGTGA AGTTCCTATT CTCTAGAAAG

5761 TATAGGAACT TCTGAAGTGG GGATTTAAAG TCGGTGTCCG CGCTGAGGGT TTAATGGCGC

5821 GCCGCGGCCG CCCGCGGTGT TGGAATAAAA ATCAACTATC ATCTACTAAC TAGTATTTAC

5881 GTTACTAGTA TATTATCATA TACGGTGTTA GAAGATGACG CAAATGATGA GAAATAGTCA

5941 TCTAAATTAG TGGAAGCTGA AACGCAAGGA TTGATAATGT AATAGGATCA ATGAATATTA

6001 ACGCTAGCAT TAAGTCCTCA GCGAGCACGC TGCTTCATGG AATGCGTGCG ATGAGCGACC

6061 TCATGCTATA CCTGAGAAAG CAACCTGACC TACAGGAAAG AGTTACTCAA GAATAAGAAT

6121 TTTCGTTTTA AAACCTAAGA GTCACTTTAA AATTTGTATA CACTTATTTT TTTTATAACT

6181 TATTTAATAA TAAAAATCAT AAATCATAAG AAATTCGCTT ATTTAGAAGT GTCAACAACG

6241 TATCTACCAA CGGAATG

**pCfB2990_(pTY3-KlLEU2-deg) 6289 bp DNA circular UNA**

FEATURES Location/Qualifiers

Misc._feature 1..14

/label="USER cassette"

Terminator 20..190

/label="T CYC1"

Misc._feature 257..316

/label=loxP

Terminator complement(317..439)

/label="T URA3"

Misc._structure complement(440..490)

/label="Degradation tag"

CDS complement(491..1576)

/label="KlLEU2syn without STOP codon"

Promoter complement(1577..2606)

/label="P LEU2"

Misc._feature 2623..2656

/label=loxP

Misc._feature 2796..2996

/label="TY3 3'"

/label="Consensus TY3 3'"

Replication_ori complement(3777..4420)

/label="pUC ori"

CDS complement(4523..5381)

/label=AmpR

Misc._feature 5825..6058

/label="TY3 5'"

/label="Consensus TY3 5'"

Terminator complement(6085..6279)

/label="T ADH1"

ORIGIN

1 CGTGCGATCG CGTGCATTCA TCCGCTCTAA CCGAAAAGGA AGGAGTTAGA CAACCTGAAG

61 TCTAGGTCCC TATTTATTTT TTTATAGTTA TGTTAGTATT AAGAACGTTA TTTATATTTC

121 AAATTTTTCT TTTTTTTCTG TACAGACGCG TGTACGCATG TAACATTATA CTGAAAACCT

181 TGCTTGAGAA GGTTTTGGGA CGCTCGAAGA TCGCGTCAGC TGAAGCTTCG TACGCTGCAG

241 GTCGACAACC CTTAATGTCG ACAACCCTTA ATATAACTTC GTATAATGTA TGCTATACGA

301 AGTTATTAGG TCTAGAGATC CCAATACAAC AGATCACGTG ATCTTTTGTA AGATGAAGTT

361 GAAGTGAGTG TTGCACCGTG CCAATGCAGG TGGCTATTAG ATTAAATATG TGATTTGTTC

421 TATTAAGTTT CCTGTATAAT TACAAATGAA TAACGAAATG AGACAAAGAA GAGAACCAAU

481 TTTTACAAGC AGCCAAGATT TCCTTGACAG CCTTGGCGAT AGCATCGCCA ACCTCAGTGG

541 TAGAGTTAGA ACCACCAAGG TCACCGGTTC TGACACCTGC ATCCAAGACA TTTCTAACAG

601 CTTCTTCAAG AGCCCTACCT TCTTCAACCA AATCCAAGGA TAACTTCAAC ATCATAGCTG

661 CAGATAAGAT GGTAGCAATT GGGTTAACCT TGTTTGCTGG TAAATCTGGG GCAGAACCAT

721 GACATGGTTC GTACAAACCG AATGCCTTGT TAGTGTCAGG TAGGGAAGCT AGAGATGCAG

781 AAGGTAATAA ACCCAAAGAA CCTGGAATAA CAGAGGCTTC ATCGGAGATA ATATCACCAA

841 ACATGTTGTT GGTAATAACA ACACCGTTTA GCTTAGTTGG TGATTTAACC AAAATCATAG

901 CAGCAGAGTC GATCAATTGG TGCTGAACAG TTAATTGTGG GAACTCAGTC TTGATGGTTT

961 CTTCAACAGT CTTTCTCCAC AATCTGGAAG AGGCAAGCAC GTTAGCCTTG TCAAGTGACC

1021 AGATTGGTAA TGGTGGGTTT TGTTGCAATG CCAAGAAAGC AGCCATTCTT GTAATTCTTT

1081 GAACTTCAGG AACACTGTAT TTCTCAGAGT CCCAAGCAAC TCCGTCACCT TCATCTTCTT

1141 TTCTTTCACC AAAGTAGATA CCACCAACCA ATTCTCTAAC GACGACGAAA TCGGTACCCT

1201 TTGCATATTC AGGCTTCAAA GGAGAAAGAT CTAGTAAAGA ATCAGAAGCA AAGTTACATG

1261 GTCTTAAGTT GGCGTATAGA CCCAATTCCT TTCTGATCTT CAATAGACCT TGTTCTGGTC

1321 TAACTGCGCC CGTACCCCAT TTTGGACCAC CAACAGCACC TAGTAAGACA GCATCGGCTT

1381 TCTTAGAGGC TTCTAGAGCT TCATCTGGTA AAGGAGTGCC AGTGGCATCG ATGGCAGCAC

1441 CCCCGATCAA GTGATGTTGG AAATTGAACT TAATTTCTGG ACGGACTTCA GCAATGGCAT

1501 TCAAGACCTT AATAGCTTCG TCAGTAACTT CTTTACCGAC GTGATCACCC GGTAGGACAA

1561 CGATATTCTT AGACATGGTA TAATCTGTGT AGTGTGGGAT ACTTTTTACT TCTTCAAATA

1621 GGTATCAACA ATAAAAATAA ATCGAAGCAA ATGTAGGAAT GCGTTAAAGC AGATGTACTT

1681 ACTTAGAGTA CATAGATATA TTTATATAAT TCAATATATA AAAGTATATG AAGCATCTGA

1741 TGTTGAACCT GTCATGACTC TAAACTGGAA TGGGTAGTTA TGCTTCTGAA GGTTTTCCGT

1801 TAGAAAATGA GTGCAGGGAT CAGGCCCGGA ACCGGTTTTA GCCTGAAAGG AAAAGAAAAA

1861 AATGCGGTGA TTACTAGCAC GTGACTGCGC TGAATTGGAA TCCACCACAA GTACAGCGAA

1921 TGGCAAGAGG GAAGGGGAAT ATATTGTGCC TGGCGGTAGT CTGCTATATC AAATGTTAAA

1981 CATAACTAAA TGCAGTAGTG AATAGAGTAA TGTATTTCTA CATATGTGGG TAGGGCCAAT

2041 GGGAGCCCGA TGTCTATTGT ACAGATATTT TCCTTTTATA GTTAATCAAT TCAACTTGGA

2101 AATCATTGAG TCGATATCTA CTTGGATAAC TGGAGCCGTG GCAGCGTTTG AAATTTGACG

2161 AGTCTCCTTG TTTTGTTCGC GCATTTTCAG TTTATTCAAA CGGGAGATGG CACCGAATCC

2221 ACTGACTTCT CTGTTCAATT TGAATGGTAA TAGGAAGAAA GTACATTTCG AGAAATTTTC

2281 GCCCAACGTC GAAATCTCAT GGATGATATC CTCGATACAA ATGACACCTT CATCACCTAA

2341 TTTCTCTTCA ACGATATTGT TGTCATTCAA GATTATCTCT TTTGGTTCTT TATCTTCTGG

2401 CCTTTGCCAC ATTATTCTAG ATCTCTTTTG GATTAAAGAA CGGATAGATG CAAGTGAAGG

2461 TTGTCCGATA ACAATATATG GTGATACAAG TTTCAACAAT GGGTACACGT CCTTGGTAAG

2521 TTTGATGAAA ACACCAGTGT TCAACTCAAC AAGTCTCAAA AGAGATAACA CTTTGTAAGC

2581 CTTTGCTGGG ATCTTCACAG CGAGCTCTCG AGAACCCTTA ATATAACTTC GTATAATGTA

2641 TGCTATACGA AGTTATTAGG TGATATCAGA TCCACTAGTG GCCTATGCAC CCAATTCGCC

2701 CTATAGTGAG TCGTATTACG CGCGCTCACT GGCCGTCGTT TTACAACGTC GTGACTGGGA

2761 AAACCCTGGC GTTACCCCTG CAGGACAGAC GTCATTAGTG CTGAGGCATT AATTGATCAG

2821 ATTCCGCGCT TCCACCACTT AGTATGATTC ATATTTTATA TAATATATAA GATAAGTAAC

2881 ATTCCGTGAA TTAATCTGAT AAACTGTTTT GACAACTGGT TACTTCCCTA AGACTGTTTA

2941 TATTAGGATT GTCAAGACAC TCCGGTATTA CTCGAGCCCG TAATACAACA AGATCTGCGG

3001 CCGCACTCAG ACCTGAAGTG AAGTTCCTAT ACTTTCTAGA GAATAGGAAC TTCTATAGTG

3061 AGTCGAATAA GGGCGACACA AAATTTATTC TAAATGCATA ATAAATACTG ATAACATCTT

3121 ATAGTTTGTA TTATATTTTG TATTATCGTT GACATGTATA ATTTTGATAT CAAAAACTGA

3181 TTTTCCCTTT ATTATTTTCG AGATTTATTT TCTTAATTCT CTTTAACAAA CTAGAAATAT

3241 TGTATATACA AAAAATCATA AATAATAGAT GAATAGTTTA ATTATAGGTG TTCATCAATC

3301 GAAAAAGCAA CGTATCTTAT TTAAAGTGCG TTGCTTTTTT CTCATTTATA AGGTTAAATA

3361 ATTCTCATAT ATCAAGCAAA GTGACAGGCG CCCTTAAATA TTCTGACAAA TGCTCTTTCC

3421 CTAAACTCCC CCCATAAAAA AACCCGCCGA AGCGGGTTTT TACGTTATTT GCGGATTAAC

3481 GATTACTCGT TATCAGAACC GCCCAGGGGG CCCGAGCTTA AGACTGGCCG TCGTTTTACA

3541 ACACAGAAAG AGTTTGTAGA AACGCAAAAA GGCCATCCGT CAGGGGCCTT CTGCTTAGTT

3601 TGATGCCTGG CAGTTCCCTA CTCTCGCCTT CCGCTTCCTC GCTCACTGAC TCGCTGCGCT

3661 CGGTCGTTCG GCTGCGGCGA GCGGTATCAG CTCACTCAAA GGCGGTAATA CGGTTATCCA

3721 CAGAATCAGG GGATAACGCA GGAAAGAACA TGTGAGCAAA AGGCCAGCAA AAGGCCAGGA

3781 ACCGTAAAAA GGCCGCGTTG CTGGCGTTTT TCCATAGGCT CCGCCCCCCT GACGAGCATC

3841 ACAAAAATCG ACGCTCAAGT CAGAGGTGGC GAAACCCGAC AGGACTATAA AGATACCAGG

3901 CGTTTCCCCC TGGAAGCTCC CTCGTGCGCT CTCCTGTTCC GACCCTGCCG CTTACCGGAT

3961 ACCTGTCCGC CTTTCTCCCT TCGGGAAGCG TGGCGCTTTC TCATAGCTCA CGCTGTAGGT

4021 ATCTCAGTTC GGTGTAGGTC GTTCGCTCCA AGCTGGGCTG TGTGCACGAA CCCCCCGTTC

4081 AGCCCGACCG CTGCGCCTTA TCCGGTAACT ATCGTCTTGA GTCCAACCCG GTAAGACACG

4141 ACTTATCGCC ACTGGCAGCA GCCACTGGTA ACAGGATTAG CAGAGCGAGG TATGTAGGCG

4201 GTGCTACAGA GTTCTTGAAG TGGTGGGCTA ACTACGGCTA CACTAGAAGA ACAGTATTTG

4261 GTATCTGCGC TCTGCTGAAG CCAGTTACCT TCGGAAAAAG AGTTGGTAGC TCTTGATCCG

4321 GCAAACAAAC CACCGCTGGT AGCGGTGGTT TTTTTGTTTG CAAGCAGCAG ATTACGCGCA

4381 GAAAAAAAGG ATCTCAAGAA GATCCTTTGA TCTTTTCTAC GGGGTCTGAC GCTCAGTGGA

4441 ACGACGCGCG CGTAACTCAC GTTAAGGGAT TTTGGTCATG AGCTTGCGCC GTCCCGTCAA

4501 GTCAGCGTAA TGCTCTGCTT TTACCAATGC TTAATCAGTG AGGCACCTAT CTCAGCGATC

4561 TGTCTATTTC GTTCATCCAT AGTTGCCTGA CTCCCCGTCG TGTAGATAAC TACGATACGG

4621 GAGGGCTTAC CATCTGGCCC CAGCGCTGCG ATGATACCGC GAGAACCACG CTCACCGGCT

4681 CCGGATTTAT CAGCAATAAA CCAGCCAGCC GGAAGGGCCG AGCGCAGAAG TGGTCCTGCA

4741 ACTTTATCCG CCTCCATCCA GTCTATTAAT TGTTGCCGGG AAGCTAGAGT AAGTAGTTCG

4801 CCAGTTAATA GTTTGCGCAA CGTTGTTGCC ATCGCTACAG GCATCGTGGT GTCACGCTCG

4861 TCGTTTGGTA TGGCTTCATT CAGCTCCGGT TCCCAACGAT CAAGGCGAGT TACATGATCC

4921 CCCATGTTGT GCAAAAAAGC GGTTAGCTCC TTCGGTCCTC CGATCGTTGT CAGAAGTAAG

4981 TTGGCCGCAG TGTTATCACT CATGGTTATG GCAGCACTGC ATAATTCTCT TACTGTCATG

5041 CCATCCGTAA GATGCTTTTC TGTGACTGGT GAGTACTCAA CCAAGTCATT CTGAGAATAG

5101 TGTATGCGGC GACCGAGTTG CTCTTGCCCG GCGTCAATAC GGGATAATAC CGCGCCACAT

5161 AGCAGAACTT TAAAAGTGCT CATCATTGGA AAACGTTCTT CGGGGCGAAA ACTCTCAAGG

5221 ATCTTACCGC TGTTGAGATC CAGTTCGATG TAACCCACTC GTGCACCCAA CTGATCTTCA

5281 GCATCTTTTA CTTTCACCAG CGTTTCTGGG TGAGCAAAAA CAGGAAGGCA AAATGCCGCA

5341 AAAAAGGGAA TAAGGGCGAC ACGGAAATGT TGAATACTCA TATTCTTCCT TTTTCAATAT

5401 TATTGAAGCA TTTATCAGGG TTATTGTCTC ATGAGCGGAT ACATATTTGA ATGTATTTAG

5461 AAAAATAAAC AAATAGGGGT CAGTGTTACA ACCAATTAAC CAATTCTGAA CATTATCGCG

5521 AGCCCATTTA TACCTGAATA TGGCTCATAA CACCCCTTGT TTGCCTGGCG GCAGTAGCGC

5581 GGTGGTCCCA CCTGACCCCA TGCCGAACTC AGAAGTGAAA CGCCGTAGCG CCGATGGTAG

5641 TGTGGGGACT CCCCATGCGA GAGTAGGGAA CTGCCAGGCA TCAAATAAAA CGAAAGGCTC

5701 AGTCGAAAGA CTGGGCCTTT CGCCCGGGCT AATTATGGGG TGTCGCCCTT ATTCGACTCT

5761 ATAGTGAAGT TCCTATTCTC TAGAAAGTAT AGGAACTTCT GAAGTGGGGA TTTAAAGTCG

5821 GTGTCCGCGC TGAGGGTTTA ATGGCGCGCC GCGGCCGCCC GCGGTGTTGT ATCTCAAAAT

5881 GAGATATGTC AGTATGACAA TACGTCATCC TGAACGTTCA TAAAACACAT ATGAAACAAC

5941 CTTATAACAA AACGAACAAC ATGAGACAAA ACCCGTCCTT CCCTAGCTGA ACTACCCAAA

6001 AGTATAAATG CCTGAACAAT TAGTTTAGAT CCGAGCTAGC ATTAAGTCCT CAGCGAGCAC

6061 GCTGCTTCAT GGAATGCGTG CGATGAGCGA CCTCATGCTA TACCTGAGAA AGCAACCTGA

6121 CCTACAGGAA AGAGTTACTC AAGAATAAGA ATTTTCGTTT TAAAACCTAA GAGTCACTTT

6181 AAAATTTGTA TACACTTATT TTTTTTATAA CTTATTTAAT AATAAAAATC ATAAATCATA

6241 AGAAATTCGC TTATTTAGAA GTGTCAACAA CGTATCTACC AACGGAATG

**pCfB2803_(pTY4-KlLEU2-deg) 6312 bp DNA circular UNA**

FEATURES Location/Qualifiers

Misc._feature 1..14

/label="USER cassette"

Terminator 20..209

/label="T CYC1"

Misc._feature 257..316

/label=loxP

Terminator complement(317..439)

/label="T URA3"

Misc._structure complement(440..490)

/label="Degradation tag"

CDS complement(491..1576)

/label="KlLEU2syn without STOP codon"

Promoter complement(1577..2606)

/label="P LEU2"

Misc._feature 2623..2656

/label=loxP

Misc._feature 2811..2996

/label="Consensus TY4 3'"

Replication_ori complement(3794..4437)

/label="pUC ori"

CDS complement(4540..5398)

/label=AmpR

Misc._feature 5879..6063

/label="Consensus TY4 5'"

Terminator complement(6108..6302)

/label="T ADH1"

ORIGIN

1 CGTGCGATCG CGTGCATTCA TCCGCTCTAA CCGAAAAGGA AGGAGTTAGA CAACCTGAAG

61 TCTAGGTCCC TATTTATTTT TTTATAGTTA TGTTAGTATT AAGAACGTTA TTTATATTTC

121 AAATTTTTCT TTTTTTTCTG TACAGACGCG TGTACGCATG TAACATTATA CTGAAAACCT

181 TGCTTGAGAA GGTTTTGGGA CGCTCGAAGA TCGCGTCAGC TGAAGCTTCG TACGCTGCAG

241 GTCGACAACC CTTAATGTCG ACAACCCTTA ATATAACTTC GTATAATGTA TGCTATACGA

301 AGTTATTAGG TCTAGAGATC CCAATACAAC AGATCACGTG ATCTTTTGTA AGATGAAGTT

361 GAAGTGAGTG TTGCACCGTG CCAATGCAGG TGGCTATTAG ATTAAATATG TGATTTGTTC

421 TATTAAGTTT CCTGTATAAT TACAAATGAA TAACGAAATG AGACAAAGAA GAGAACCAAU

481 TTTTACAAGC AGCCAAGATT TCCTTGACAG CCTTGGCGAT AGCATCGCCA ACCTCAGTGG

541 TAGAGTTAGA ACCACCAAGG TCACCGGTTC TGACACCTGC ATCCAAGACA TTTCTAACAG

601 CTTCTTCAAG AGCCCTACCT TCTTCAACCA AATCCAAGGA TAACTTCAAC ATCATAGCTG

661 CAGATAAGAT GGTAGCAATT GGGTTAACCT TGTTTGCTGG TAAATCTGGG GCAGAACCAT

721 GACATGGTTC GTACAAACCG AATGCCTTGT TAGTGTCAGG TAGGGAAGCT AGAGATGCAG

781 AAGGTAATAA ACCCAAAGAA CCTGGAATAA CAGAGGCTTC ATCGGAGATA ATATCACCAA

841 ACATGTTGTT GGTAATAACA ACACCGTTTA GCTTAGTTGG TGATTTAACC AAAATCATAG

901 CAGCAGAGTC GATCAATTGG TGCTGAACAG TTAATTGTGG GAACTCAGTC TTGATGGTTT

961 CTTCAACAGT CTTTCTCCAC AATCTGGAAG AGGCAAGCAC GTTAGCCTTG TCAAGTGACC

1021 AGATTGGTAA TGGTGGGTTT TGTTGCAATG CCAAGAAAGC AGCCATTCTT GTAATTCTTT

1081 GAACTTCAGG AACACTGTAT TTCTCAGAGT CCCAAGCAAC TCCGTCACCT TCATCTTCTT

1141 TTCTTTCACC AAAGTAGATA CCACCAACCA ATTCTCTAAC GACGACGAAA TCGGTACCCT

1201 TTGCATATTC AGGCTTCAAA GGAGAAAGAT CTAGTAAAGA ATCAGAAGCA AAGTTACATG

1261 GTCTTAAGTT GGCGTATAGA CCCAATTCCT TTCTGATCTT CAATAGACCT TGTTCTGGTC

1321 TAACTGCGCC CGTACCCCAT TTTGGACCAC CAACAGCACC TAGTAAGACA GCATCGGCTT

1381 TCTTAGAGGC TTCTAGAGCT TCATCTGGTA AAGGAGTGCC AGTGGCATCG ATGGCAGCAC

1441 CCCCGATCAA GTGATGTTGG AAATTGAACT TAATTTCTGG ACGGACTTCA GCAATGGCAT

1501 TCAAGACCTT AATAGCTTCG TCAGTAACTT CTTTACCGAC GTGATCACCC GGTAGGACAA

1561 CGATATTCTT AGACATGGTA TAATCTGTGT AGTGTGGGAT ACTTTTTACT TCTTCAAATA

1621 GGTATCAACA ATAAAAATAA ATCGAAGCAA ATGTAGGAAT GCGTTAAAGC AGATGTACTT

1681 ACTTAGAGTA CATAGATATA TTTATATAAT TCAATATATA AAAGTATATG AAGCATCTGA

1741 TGTTGAACCT GTCATGACTC TAAACTGGAA TGGGTAGTTA TGCTTCTGAA GGTTTTCCGT

1801 TAGAAAATGA GTGCAGGGAT CAGGCCCGGA ACCGGTTTTA GCCTGAAAGG AAAAGAAAAA

1861 AATGCGGTGA TTACTAGCAC GTGACTGCGC TGAATTGGAA TCCACCACAA GTACAGCGAA

1921 TGGCAAGAGG GAAGGGGAAT ATATTGTGCC TGGCGGTAGT CTGCTATATC AAATGTTAAA

1981 CATAACTAAA TGCAGTAGTG AATAGAGTAA TGTATTTCTA CATATGTGGG TAGGGCCAAT

2041 GGGAGCCCGA TGTCTATTGT ACAGATATTT TCCTTTTATA GTTAATCAAT TCAACTTGGA

2101 AATCATTGAG TCGATATCTA CTTGGATAAC TGGAGCCGTG GCAGCGTTTG AAATTTGACG

2161 AGTCTCCTTG TTTTGTTCGC GCATTTTCAG TTTATTCAAA CGGGAGATGG CACCGAATCC

2221 ACTGACTTCT CTGTTCAATT TGAATGGTAA TAGGAAGAAA GTACATTTCG AGAAATTTTC

2281 GCCCAACGTC GAAATCTCAT GGATGATATC CTCGATACAA ATGACACCTT CATCACCTAA

2341 TTTCTCTTCA ACGATATTGT TGTCATTCAA GATTATCTCT TTTGGTTCTT TATCTTCTGG

2401 CCTTTGCCAC ATTATTCTAG ATCTCTTTTG GATTAAAGAA CGGATAGATG CAAGTGAAGG

2461 TTGTCCGATA ACAATATATG GTGATACAAG TTTCAACAAT GGGTACACGT CCTTGGTAAG

2521 TTTGATGAAA ACACCAGTGT TCAACTCAAC AAGTCTCAAA AGAGATAACA CTTTGTAAGC

2581 CTTTGCTGGG ATCTTCACAG CGAGCTCTCG AGAACCCTTA ATATAACTTC GTATAATGTA

2641 TGCTATACGA AGTTATTAGG TGATATCAGA TCCACTAGTG GCCTATGCAC CCAATTCGCC

2701 CTATAGTGAG TCGTATTACG CGCGCTCACT GGCCGTCGTT TTACAACGTC GTGACTGGGA

2761 AAACCCTGGC GTTACCCCTG CAGGACTAGT GCTGAGGCAT TAATTGATCA GGTAGGTACA

2821 TATATGAGGA ATATGAGTCG TCACATCAAT GTATAGTAAC TACCGGAATC ACTATTATAT

2881 TGGTCATGAT TAATATGACC AATCGGCGTG TGTTTTATAT ACCTCTCTTA TTTAGTATAA

2941 GAAGATCAGT ACTCACTTCT TCATTAATAC TAATTTTTAA CCTCTAATTA TCAACAAGAT

3001 CTGCGGCCGC GGCCGCAAAT TTAAATAAAA TGAAGTGAAG TTCCTATACT TTCTAGAGAA

3061 TAGGAACTTC TATAGTGAGT CGAATAAGGG CGACACAAAA TTTATTCTAA ATGCATAATA

3121 AATACTGATA ACATCTTATA GTTTGTATTA TATTTTGTAT TATCGTTGAC ATGTATAATT

3181 TTGATATCAA AAACTGATTT TCCCTTTATT ATTTTCGAGA TTTATTTTCT TAATTCTCTT

3241 TAACAAACTA GAAATATTGT ATATACAAAA AATCATAAAT AATAGATGAA TAGTTTAATT

3301 ATAGGTGTTC ATCAATCGAA AAAGCAACGT ATCTTATTTA AAGTGCGTTG CTTTTTTCTC

3361 ATTTATAAGG TTAAATAATT CTCATATATC AAGCAAAGTG ACAGGCGCCC TTAAATATTC

3421 TGACAAATGC TCTTTCCCTA AACTCCCCCC ATAAAAAAAC CCGCCGAAGC GGGTTTTTAC

3481 GTTATTTGCG GATTAACGAT TACTCGTTAT CAGAACCGCC CAGGGGGCCC GAGCTTAAGA

3541 CTGGCCGTCG TTTTACAACA CAGAAAGAGT TTGTAGAAAC GCAAAAAGGC CATCCGTCAG

3601 GGGCCTTCTG CTTAGTTTGA TGCCTGGCAG TTCCCTACTC TCGCCTTCCG CTTCCTCGCT

3661 CACTGACTCG CTGCGCTCGG TCGTTCGGCT GCGGCGAGCG GTATCAGCTC ACTCAAAGGC

3721 GGTAATACGG TTATCCACAG AATCAGGGGA TAACGCAGGA AAGAACATGT GAGCAAAAGG

3781 CCAGCAAAAG GCCAGGAACC GTAAAAAGGC CGCGTTGCTG GCGTTTTTCC ATAGGCTCCG

3841 CCCCCCTGAC GAGCATCACA AAAATCGACG CTCAAGTCAG AGGTGGCGAA ACCCGACAGG

3901 ACTATAAAGA TACCAGGCGT TTCCCCCTGG AAGCTCCCTC GTGCGCTCTC CTGTTCCGAC

3961 CCTGCCGCTT ACCGGATACC TGTCCGCCTT TCTCCCTTCG GGAAGCGTGG CGCTTTCTCA

4021 TAGCTCACGC TGTAGGTATC TCAGTTCGGT GTAGGTCGTT CGCTCCAAGC TGGGCTGTGT

4081 GCACGAACCC CCCGTTCAGC CCGACCGCTG CGCCTTATCC GGTAACTATC GTCTTGAGTC

4141 CAACCCGGTA AGACACGACT TATCGCCACT GGCAGCAGCC ACTGGTAACA GGATTAGCAG

4201 AGCGAGGTAT GTAGGCGGTG CTACAGAGTT CTTGAAGTGG TGGGCTAACT ACGGCTACAC

4261 TAGAAGAACA GTATTTGGTA TCTGCGCTCT GCTGAAGCCA GTTACCTTCG GAAAAAGAGT

4321 TGGTAGCTCT TGATCCGGCA AACAAACCAC CGCTGGTAGC GGTGGTTTTT TTGTTTGCAA

4381 GCAGCAGATT ACGCGCAGAA AAAAAGGATC TCAAGAAGAT CCTTTGATCT TTTCTACGGG

4441 GTCTGACGCT CAGTGGAACG ACGCGCGCGT AACTCACGTT AAGGGATTTT GGTCATGAGC

4501 TTGCGCCGTC CCGTCAAGTC AGCGTAATGC TCTGCTTTTA CCAATGCTTA ATCAGTGAGG

4561 CACCTATCTC AGCGATCTGT CTATTTCGTT CATCCATAGT TGCCTGACTC CCCGTCGTGT

4621 AGATAACTAC GATACGGGAG GGCTTACCAT CTGGCCCCAG CGCTGCGATG ATACCGCGAG

4681 AACCACGCTC ACCGGCTCCG GATTTATCAG CAATAAACCA GCCAGCCGGA AGGGCCGAGC

4741 GCAGAAGTGG TCCTGCAACT TTATCCGCCT CCATCCAGTC TATTAATTGT TGCCGGGAAG

4801 CTAGAGTAAG TAGTTCGCCA GTTAATAGTT TGCGCAACGT TGTTGCCATC GCTACAGGCA

4861 TCGTGGTGTC ACGCTCGTCG TTTGGTATGG CTTCATTCAG CTCCGGTTCC CAACGATCAA

4921 GGCGAGTTAC ATGATCCCCC ATGTTGTGCA AAAAAGCGGT TAGCTCCTTC GGTCCTCCGA

4981 TCGTTGTCAG AAGTAAGTTG GCCGCAGTGT TATCACTCAT GGTTATGGCA GCACTGCATA

5041 ATTCTCTTAC TGTCATGCCA TCCGTAAGAT GCTTTTCTGT GACTGGTGAG TACTCAACCA

5101 AGTCATTCTG AGAATAGTGT ATGCGGCGAC CGAGTTGCTC TTGCCCGGCG TCAATACGGG

5161 ATAATACCGC GCCACATAGC AGAACTTTAA AAGTGCTCAT CATTGGAAAA CGTTCTTCGG

5221 GGCGAAAACT CTCAAGGATC TTACCGCTGT TGAGATCCAG TTCGATGTAA CCCACTCGTG

5281 CACCCAACTG ATCTTCAGCA TCTTTTACTT TCACCAGCGT TTCTGGGTGA GCAAAAACAG

5341 GAAGGCAAAA TGCCGCAAAA AAGGGAATAA GGGCGACACG GAAATGTTGA ATACTCATAT

5401 TCTTCCTTTT TCAATATTAT TGAAGCATTT ATCAGGGTTA TTGTCTCATG AGCGGATACA

5461 TATTTGAATG TATTTAGAAA AATAAACAAA TAGGGGTCAG TGTTACAACC AATTAACCAA

5521 TTCTGAACAT TATCGCGAGC CCATTTATAC CTGAATATGG CTCATAACAC CCCTTGTTTG

5581 CCTGGCGGCA GTAGCGCGGT GGTCCCACCT GACCCCATGC CGAACTCAGA AGTGAAACGC

5641 CGTAGCGCCG ATGGTAGTGT GGGGACTCCC CATGCGAGAG TAGGGAACTG CCAGGCATCA

5701 AATAAAACGA AAGGCTCAGT CGAAAGACTG GGCCTTTCGC CCGGGCTAAT TATGGGGTGT

5761 CGCCCTTATT CGACTCTATA GTGAAGTTCC TATTCTCTAG AAAGTATAGG AACTTCTGAA

5821 GTGGGGATTT AAATGCGGCC GCGCTGAGGG TTTAATGGCG CGCCGCGGCC GCCCGCGGTG

5881 TTGGAACGAG AGTAATTAAT AGTGACATGA GTTGCTATGG TAACAATCTA ATGCTTACAT

5941 CGTATATTAA TGTACAACTC GTATACGTTT AAGTGTGATT GCGCCTATTG CAGAAGGAAT

6001 GTTAAACGAG AAGCTCAGAC AATACTGAAG CTGTGTTAAA GACCTATTAG TTGAACATGT

6061 TATGCTAGCA TTAAGTCCTC AGCGAGCTCG CATGGAATGC GTGCGATGAG CGACCTCATG

6121 CTATACCTGA GAAAGCAACC TGACCTACAG GAAAGAGTTA CTCAAGAATA AGAATTTTCG

6181 TTTTAAAACC TAAGAGTCAC TTTAAAATTT GTATACACTT ATTTTTTTTA TAACTTATTT

6241 AATAATAAAA ATCATAAATC ATAAGAAATT CGCTTATTTA GAAGTGTCAA CAACGTATCT

6301 ACCAACGGAA TG
