# Supplementary material for: EasyCloneMulti: A Set of Vectors for Simultaneous and Multiple Genomic Integrations in Saccharomyces cerevisiae
Source: PLoS One. 2016 Mar 2;11(3):e0150394. doi: 10.1371/journal.pone.0150394 (PMC4775045; doi:10.1371/journal.pone.0150394)
Supplement: S2 File — (DOCX) [file pone.0150394.s005.docx]

## Supplementary References

1. Borodina I, Kildegaard KR, Jensen NB, Blicher TH, Maury J, Sherstyk S, et al. Establishing a synthetic pathway for high-level production of 3-hydroxypropionic acid in *Saccharomyces cerevisiae* via β-alanine. Metab Eng.; 2014; 1–8. doi:10.1016/j.ymben.2014.10.003

2. Jensen NB, Strucko T, Kildegaard KR, David F, Maury J, Mortensen UH, et al. EasyClone: method for iterative chromosomal integration of multiple genes in *Saccharomyces cerevisiae*. FEMS Yeast Res. 2014; 1–11. doi:10.1111/1567-1364.12118

3. Gilon T, Chomsky O, Kulka RG. Degradation signals for ubiquitin system proteolysis in *Saccharomyces cerevisiae*. EMBO J.; 1998;17: 2759–66. doi:10.1093/emboj/17.10.2759

4. Mikkelsen MD, Buron LD, Salomonsen B, Olsen CE, Hansen BG, Mortensen UH, et al. Microbial production of indolylglucosinolate through engineering of a multi-gene pathway in a versatile yeast expression platform. Metab Eng.; 2012;14: 104–11. doi:10.1016/j.ymben.2012.01.006

5. Nørholm MHH. A mutant Pfu DNA polymerase designed for advanced uracil-excision DNA engineering. BMC Biotechnol. 2010;10: 21. doi: 10.1186/1472-6750-10-21
