# Supplementary material for: EasyCloneMulti: A Set of Vectors for Simultaneous and Multiple Genomic Integrations in Saccharomyces cerevisiae
Source: PLoS One. 2016 Mar 2;11(3):e0150394. doi: 10.1371/journal.pone.0150394 (PMC4775045; doi:10.1371/journal.pone.0150394)
Supplement: S1 Table — (DOCX) [file pone.0150394.s006.docx]

**Supplementary Table S1: List of the consensus sequences**

| **Name** | **Sequence (5’-3’)** |
| --- | --- |
|  |  |
| Ty1Cons1 | *TGTTGGAATAAAAATCCACTATCGTCTATCAACTAATAGTTATATTATCAATATATTATCATATACGGTGTTAAGATGATGACATAAGTTATGAGAAGCTGTCATCGAAGTTAGAGGAAGCTGAAGTGCAAGGATTGATAATGTAATAGGATCAATGAATATAAACATAT***AAAACGGAATGAGGAATAATCGTAATATTAGTATGTAGAAATATAGATTCCATTTTGAGGATTCCTATATCCTCGAGGAGAACTTCTAGTGTATATTCTGTATACCTAATATTATAGCCTTTATCAACAATGGAATCCCAACAATTATCTAATTACCCACAAATTTCTCA** |
| Ty1Cons2 | *TGTTGGAATAAAAATCAACTATCATCTACTAACTAGTATTTACGTTACTAGTATATTATCATATACGGTGTTAGAAGATGACGCAAATGATGAGAAATAGTCATCTAAATTAGTGGAAGCTGAAACGCAAGGATTGATAATGTAATAGGATCAATGAATATTAACATA***TAAAACGGAATGATGAATAATATTTATAGAATTGTGTAGAATTGCAGATTCCCTTTTATGGATTCCTAAATCCTCGAGGAGAACTTCTAGTATATTCTGTATACCTAATATTATAGCCTTTATCAACAATGGAATCCCAACAATTATCTCAAAATTCACATATTTCTCA** |
| Ty2Cons | *TGTTGGAATAAAAATCAACTATCATCTACTAACTAGTATTTACGTTACTAGTATATTATCATATACGGTGTTAGAAGATGACGCAAATGATGAGAAATAGTCATCTAAATTAGTGGAAGCTGAAACGCAAGGATTGATAATGTAATAGGATCAATGAATATTAAC***ATATAAAATGATGATAATAATATTTATAGAATTGTGTAGAATTGCAGATTCCCTTTTATGGATTCCTAAATCCTBGAGGAGAACTTCTAGTATATTCTACATACCTAATATTATTGCCTTATTAAAAATGGAATCCCAACAATTACATCAAAATCCACATTCTC** |
| Ty3Cons | *TGTTGTATCTCAAAATGAGATATGTCAGTATGACAATACGTCATCCTGAACGTTCATAAAACACATATGAAACAACCTTATAACAAAACGAACAACATGAGACAAAACCCGTCCTTCCCTAGCTGAACTACCCAAAAGTATAAATGCCTGAACAATTAGTTTAGATCCGA***GATTCCGCGCTTCCACCACTTAGTATGATTCATATTTTATATAATATATAAGATAAGTAACATTCCGTGAATTAATCTGATAAACTGTTTTGACAACTGGTTACTTCCCTAAGACTGTTTATATTAGGATTGTCAAGACACTCCGGTATTACTCGAGCCCGTAATACAACA** |
| Ty4Cons | *TGTTGGAACGAGAGTAATTAATAGTGACATGAGTTGCTATGGTAACAATCTAATGCTTACATCGTATATTAATGTACAACTCGTATACGTTTAAGTGTGATTGCGCCTATTGCAGAAGGAATGTTAAACGAGAAGCTCAGACAATACTGAAGCTGTGTTAAAGACCTATTAGTTGAACATGTTAT***GGTAGGTACATATATGAGGAATATGAGTCGTCACATCAATGTATAGTAACTACCGGAATCACTATTATATTGGTCATGATTAATATGACCAATCGGCGTGTGTTTTATATACCTCTCTTATTTAGTATAAGAAGATCAGTACTCACTTCTTCATTAATACTAATTTTTAACCTCTAATTATCAACA** |
|  |  |

Sequences of the HR sequences present on the EasyCloneMulti vectors are specified in italic for HR 5’ and in bold for HR 3’. Sizes of HR sequences in bp (HR 5’; HR 3’): Ty1Cons1 (170;170), Ty1Cons2 (168/169), Ty2Cons (165/164), Ty3Cons (170/171), Ty4Cons (185/186).
