# Supplementary material for: EasyCloneMulti: A Set of Vectors for Simultaneous and Multiple Genomic Integrations in Saccharomyces cerevisiae
Source: PLoS One. 2016 Mar 2;11(3):e0150394. doi: 10.1371/journal.pone.0150394 (PMC4775045; doi:10.1371/journal.pone.0150394)
Supplement: S2 Table — (DOCX) [file pone.0150394.s007.docx]

**Supplementary Table S2: List of strains used in this study.**

| **Strain name** | **Genotype** | **Reference/Source** |
| --- | --- | --- |
| CEN.PK 113-5D | *MAT*a *ura3-52 HIS3 LEU2 TRP1 MAL2-8^c^ SUC2* [ura^-^] | Peter Kötter |
| CEN.PK 113-32D | *MAT*a *URA3 HIS3 leu2-3/112 TRP1 MAL2-8^c^ SUC2* [leu^-^] | Peter Kötter |
| CEN.PK 113-11A | *MAT*a *URA3 his3∆1 LEU2 trp1-289* *MAL2-8^c^ SUC2* [his^-^ trp^-^] | Peter Kötter |
| ST738 | *MAT*a *ura3-52 his3∆1 leu2-3/112 TRP1 MAL2-8^c^ SUC2* P_TEF1_::ScPYC1 P_PGK1_::ScPYC2 [ura^-^ his^-^ leu^-^] | [1] |
| SCE-iL1-155 | *MAT*a *ura3-52 his3∆1 leu2-3/112 TRP1 MAL2-8^c^ SUC2* P_TEF1_::ScPYC1 P_PGK1_::ScPYC2 P_TEF1_::BcBAPAT P_PGK1_::EcYdfG loxP-*Sp.HIS5* loxP-*Kl.LEU2* [ura^-^] | This study |

Yeast strains CEN.PK113-5D, CEN.PK113-32D and CEN.PK113-11A were used for experiments with GFP fluorescence. Yeast strain SCE-iL1-155 was used for 3HP production experiments and created by transforming strain ST738 with *Not*I-linearized pCfB257 and pCfB800.
