# Supplementary material for: EasyCloneMulti: A Set of Vectors for Simultaneous and Multiple Genomic Integrations in Saccharomyces cerevisiae
Source: PLoS One. 2016 Mar 2;11(3):e0150394. doi: 10.1371/journal.pone.0150394 (PMC4775045; doi:10.1371/journal.pone.0150394)
Supplement: S3 Table — Sequences of primers (5´ to 3´) used in this study. Overhangs used for USER cloning are underlined. (DOCX) [file pone.0150394.s008.docx]

**Supplementary Table S3: Primer sequences.**

Sequences of primers (5´ to 3´) used in this study. Overhangs used for USER cloning are underlined.

| **ID** | **Name** | **Sequence, 5´ to 3´** |
| --- | --- | --- |
| PR-5 | PTEF1_fw | ACCTGCACU TTGTAATTAAAACTTAG |
| PR-6 | PTEF1_rv | CACGCGAU GCACACACCATAGCTTC |
| PR-311 | GFPopt_fw | ATCTGTCAU AAAACAATGAGCAAAGGAGAAGAAC |
| PR-312 | GFPopt_rv | CACGCGAU TCATTTGTAGAGCTCATCCATGC |
| PR-399 | USERrev | ATTGGGU GCATAGGCCACTAGTGGATCTG |
| PR-401 | pIntFwdU | ACCCAAU TCGCCCTATAGTGAGTCG |
| PR-512 | JM076_UP5’_open | ATTAAACCCTCAGCGCGGCC |
| PR-513 | JM077_UP3’_open | ATTAAGTCCTCAGCGAGCTCG |
| PR-514 | JM078_DW5’_open | ATTAATGCCTCAGCACTAGTCC |
| PR-515 | JM079_DW3’_open | ATTAAGACCTCAGCGCGG |
| PR-517 | JM081_UP_LTR_Inf_forw | CCGCGCTGAGGGTTTAATGG |
| PR-518 | JM082_UP_LTR_Inf_rev | GCTCGCTGAGGACTTAATGC |
| PR-519 | JM083_DW_LTR_Inf_forw | TAGTGCTGAGGCATTAATTGATC |
| PR-521 | JM085_KlURA_3 _open_rev | TAACGAAATGAGACAAAGAAGAGAACCAATTTTTACAAGCATGGGGAGCGCTGATTCTCT |
| PR-522 | JM086_KlURA_3 _open_forw | TCTTTGTCTCATTTCGTTATTCATTTGTAATTATACAGGAAACTTAATAGAACAAATCAC |
| PR-526 | JM090_DW_LTR_Inf_rev bis | TTTAAATTTGCGGCCGCGGCCGCAG |
| PR-652 | TcPAND_U1_fw | AGTGCAGG**U** AAAACAATGCCAGCTACTGGTG [1] |
| PR-653 | TcPAND_U1_rv | CGTGCGA**U** TCACAAATCGGAACCCAATC [1] |
| PR-1697 | JM169_Tyx_5 _forw | AGTCGGTGU CCGCGCTGAGGGTTTAATGG |
| PR-1698 | JM170_openvec up_Tyx_5 | ACACCGACU TTAAATCCCCACTTCAGAAGTTCC |
| PR-1699 | JM171_Tyx_5 _rev | AAGCAGCGU GCTCGCTGAGGACTTAATGC |
| PR-1700 | JM172_openvec dw_Tyx_5 _rev | ACGCTGCTU CATGGAATGCGTGCGATGA |
| PR-1701 | JM173_Tyx_3 _forw | AGACGTCAU TAGTGCTGAGGCATTAATTGATC |
| PR-1702 | JM174_openvec up_Tyx_3 | ATGACGTCU GTCCTGCAGGGGTAACGCCA |
| PR-1704 | JM176_openvec dw_Tyx_3 | ACTCAGACCU GAAGTGAAGTTCCTATACTTTCTAGAG |
| PR-2051 | JM215_Tyx_3 _rev | AGGTCTGAGU GCGGCCGCAGATCTTGAG |
| PR-2848 | JM235_TADH1_USER_3' | AGCGACCU CATGCTATACCTG |
| PR-2849 | JM236_TADH1_openvec_USER_3' | AGGTCGCU CATCGCACGCA |
| PR-2850 | JM237_TCYC1_USER_3' | TGACGCGAU CTTCGAGCG |
| PR-2851 | JM238_TCYC1_openvec_USER_3' | ATCGCGU CAGCTGAAGCTTC |
| PR-9685 | open at degron_rev | ATTGGTTCU CTTCTTTGTCTCATTTC |
| PR-9686 | HIS from ptr_forw | AGGGTTCU CGAGTAGGTCTAGAGATCTGTTTAGCTTG |
| PR-9687 | HIS for fuse degron_rev | AGAACCAAU TTTTACAAGC CAACACTCCCTTCGTGCTTG |
| PR-9688 | LEU for fuse degron rev | AGAACCAAU TTTTACAAGCAGCCAAGATTTCCTTGACAGC |
| PR-9689 | open TY at lox_Forw | AGAACCCU TAATATAACTTCGTATAATGT |
| PR-9690 | open TY at degron_rev | ATTGGTTCU CTTCTTTGTCTCATTTC |
| PR-9691 | URA3 for fuse degron rev | AGAACCAAU TTTTACAAGCATG GGG AGC GCT GAT TCT CT |
