# Supplementary material for: EasyCloneMulti: A Set of Vectors for Simultaneous and Multiple Genomic Integrations in Saccharomyces cerevisiae
Source: PLoS One. 2016 Mar 2;11(3):e0150394. doi: 10.1371/journal.pone.0150394 (PMC4775045; doi:10.1371/journal.pone.0150394)
Supplement: S4 Table — (DOCX) [file pone.0150394.s009.docx]

### Supplementary Table S4: List of plasmids used in this study.

| **Name** | **Vector type** | **Targeted integration site** | **Insert** | **Selective marker**^§^ | **Degradation signal** | **Source** |
| --- | --- | --- | --- | --- | --- | --- |
| ***EasyCloneMulti vectors for cloning of constructs in this study*** | | | | | | |
| pCfB322 | integrative | Ty4Cons | USER cassette** | *Kl.URA3* | degradation signal* | This study, and [1] |
| pCfB2046 | integrative | Ty1Cons2 | USER cassette** | *Kl.URA3* | degradation signal* | This study |
| pCfB2047 | integrative | Ty2Cons | USER cassette** | *Kl.URA3* | degradation signal* | This study |
| ***EasyCloneMulti vectors for cloning with Kl.URA3****** ***marker*** | | | | | | |
| pCfB2989 | integrative | Ty1Cons1 | USER cassette** | *Kl.URA3**** | degradation signal* | This study |
| pCfB2988 | integrative | Ty1Cons2 | USER cassette** | *Kl.URA3**** | degradation signal* | This study |
| pCfB2797 | integrative | Ty2Cons | USER cassette** | *Kl.URA3**** | degradation signal* | This study |
| pCfB2875 | integrative | Ty3Cons | USER cassette** | *Kl.URA3**** | degradation signal* | This study |
| pCfB2796 | integrative | Ty4Cons | USER cassette** | *Kl.URA3**** | degradation signal* | This study |
| ***EasyCloneMulti vectors for cloning with Kl.LEU2****** ***marker*** | | | | | | |
| pCfB2992 | integrative | Ty1Cons1 | USER cassette** | *Kl.LEU2**** | degradation signal* | This study |
| pCfB2991 | integrative | Ty1Cons2 | USER cassette** | *Kl.LEU2**** | degradation signal* | This study |
| pCfB2804 | integrative | Ty2Cons | USER cassette** | *Kl.LEU2**** | degradation signal* | This study |
| pCfB2990 | integrative | Ty3Cons | USER cassette** | *Kl.LEU2**** | degradation signal* | This study |
| pCfB2803 | integrative | Ty4Cons | USER cassette** | *Kl.LEU2**** | degradation signal* | This study |
| ***EasyCloneMulti vectors containing a GFP reporter*** | | | | | | |
| pCfB1136 | integrative | Ty1Cons1 | *PTEF1*-GFP | *Kl.URA3* | degradation signal* | This study |
| pCfB1137 | integrative | Ty1Cons2 | *PTEF1*-GFP | *Kl.URA3* | degradation signal* | This study |
| pCfB1138 | integrative | Ty2Cons | *PTEF1*-GFP | *Kl.URA3* | degradation signal* | This study |
| pCfB1139 | integrative | Ty3Cons | *PTEF1*-GFP | *Kl.URA3* | degradation signal* | This study |
| pCfB326 | integrative | Ty4Cons | *PTEF1*-GFP | *Kl.URA3* | degradation signal* | This study |
| pCfB2795 | integrative | Ty1Cons1 | *PTEF1*-GFP | *Kl.URA3**** | degradation signal* | This study |
| pCfB2794 | integrative | Ty1Cons2 | *PTEF1*-GFP | *Kl.URA3**** | degradation signal* | This study |
| pCfB2793 | integrative | Ty2Cons | *PTEF1*-GFP | *Kl.URA3**** | degradation signal* | This study |
| pCfB2792 | integrative | Ty3Cons | *PTEF1*-GFP | *Kl.URA3**** | degradation signal* | This study |
| pCfB2791 | integrative | Ty4Cons | *PTEF1*-GFP | *Kl.URA3**** | degradation signal* | This study |
| pCfB2802 | integrative | Ty1Cons1 | *PTEF1*-GFP | *Kl.LEU2**** | degradation signal* | This study |
| pCfB2801 | integrative | Ty1Cons2 | *PTEF1*-GFP | *Kl.LEU2**** | degradation signal* | This study |
| pCfB2800 | integrative | Ty2Cons | *PTEF1*-GFP | *Kl.LEU2**** | degradation signal* | This study |
| pCfB2799 | integrative | Ty3Cons | *PTEF1*-GFP | *Kl.LEU2**** | degradation signal* | This study |
| pCfB2798 | integrative | Ty4Cons | *PTEF1*-GFP | *Kl.LEU2**** | degradation signal* | This study |
| pCfB2809 | integrative | Ty1Cons1 | *PTEF1*-GFP | *Sp.HIS5**** | degradation signal* | This study |
| pCfB2808 | integrative | Ty1Cons2 | *PTEF1*-GFP | *Sp.HIS5**** | degradation signal* | This study |
| pCfB2807 | integrative | Ty2Cons | *PTEF1*-GFP | *Sp.HIS5**** | degradation signal* | This study |
| pCfB2806 | integrative | Ty3Cons | *PTEF1*-GFP | *Sp.HIS5**** | degradation signal* | This study |
| pCfB2805 | integrative | Ty4Cons | *PTEF1*-GFP | *Sp.HIS5**** | degradation signal* | This study |
|  |  |  |  |  |  |  |
| ***EasyCloneMulti vectors containing Tc.panD for 3HP production*** | | | | | | |
| pCfB2099 | integrative | Ty1Cons1 | *PTEF1-Tc.panD* | *Kl.URA3* | degradation signal* | This study |
| pCfB2097 | integrative | Ty1Cons2 | *PTEF1-Tc.panD* | *Kl.URA3* | degradation signal* | This study |
| pCfB2096 | integrative | Ty2Cons | *PTEF1-Tc.panD* | *Kl.URA3* | degradation signal* | This study |
| pCfB799 | integrative | Ty4Cons | *PTEF1-Tc.panD* | *Kl.URA3* | degradation signal* | [1] |
| ***Other vectors*** | | | | | | |
| pCfB054 | episomal, 2μ |  | USER cassette** | *Kl.URA3* | no | [2] |
| pCfB255 | integrative | X-2 | USER cassette** | *Kl.URA3* | no | [2] |
| pCfB257 | integrative | X-3 | USER cassette** | *Kl.LEU2* | no | [2] |
| pCfB312 | integrative | Ty4Cons | USER cassette** | *Kl.URA3* | no | This study |
| pCfB319 | episomal, 2μ |  | *PTEF1*-GFP | *Kl.URA3* | no | This study |
| pCfB321 | integrative | Ty4Cons | *PTEF1*-GFP | *Kl.URA3* | no | This study |
| pCfB329 | integrative | X-2 | *PTEF1*-GFP | *Kl.URA3* | no | This study |
| pCfB800 | integrative | X-4 | *PTEF1*-*Bc.*BAPAT; *PPGK1*-*Ec.*YdfG | *Sp.HiS5* | no | [1] |
| pCfB2051 | CEN/ARS |  | *-* | *Kl.URA3**** | no | GeneArt® (LifeTechnologies) |
| pCfB2052 | CEN/ARS |  | *-* | *Kl.LEU2**** | no | GeneArt® (LifeTechnologies) |
| pCfB2053 | CEN/ARS |  | *-* | *Sp.HIS5**** | no | GeneArt® (LifeTechnologies) |

^§^ All selection marker genes in the EasyClone and EasyCloneMulti vectors are flanked by loxP sites. * The degradation signal has the amino acid sequence ACKNWFSSLSHFVIHL and was reported by Gilon et al. 1998. ** USER cassette refers to a cassette for uracil excision cloning based on *Asi*SI/*Nb.Bsm*I as reported by [2]. *** for these vectors, selective markers were ordered as synthetic DNA from Geneart® (LifeTechnologies).
