# Supplementary material for: EasyCloneMulti: A Set of Vectors for Simultaneous and Multiple Genomic Integrations in Saccharomyces cerevisiae
Source: PLoS One. 2016 Mar 2;11(3):e0150394. doi: 10.1371/journal.pone.0150394 (PMC4775045; doi:10.1371/journal.pone.0150394)
Supplement: S5 Table — Candidate insertion loci were identified by finding reads mapped to a specific chromosomal location whose paired end partner read mapped to a unique region (i.e. a region with no similarity to the yeast genome) on the vector insert. The table indicates all loci identified using this approach, but the candidate loci that either have narrow mapped read stacks or low coverage are not likely to be real insertion loci. The percentage of mapped reads indicates what percentage of reads identified as mapping to candidate insertion loci map to the particular location specified in the table. (DOCX) [file pone.0150394.s010.docx]

**Supplementary Table S5: Putative insertion loci identified from isolate sequencing.**

| **Sample Id** | **Chromosome** | **Location** | **Read stack width** | **Average read coverage** | **Percentage of mapped reads** |
| --- | --- | --- | --- | --- | --- |
| TY1_C1_B1 | chrI | 165975 | 36 | 5.81 | 0.11 |
| TY1_C1_B1 | chrII | 221258 | 122 | 9.21 | 0.6 |
| TY1_C1_B1 | chrIII | 82896 | 56 | 5 | 0.15 |
| TY1_C1_B1 | chrIV | 878152 | 507 | 362.32 | 98.19 |
| TY1_C1_B1 | chrV | 449135 | 32 | 5 | 0.09 |
| TY1_C1_B1 | chrVII | 817542 | 36 | 5 | 0.1 |
| TY1_C1_B1 | chrVIII | 549455 | 36 | 5 | 0.1 |
| TY1_C1_B1 | chrX | 204239 | 29 | 5 | 0.08 |
| TY1_C1_B1 | chrXII | 215225 | 44 | 6 | 0.14 |
| TY1_C1_B1 | chrXII | 476123 | 36 | 5 | 0.1 |
| TY1_C1_B1 | chrXII | 481706 | 44 | 5 | 0.12 |
| TY1_C1_B1 | chrXIII | 378415 | 74 | 5.97 | 0.24 |
| TY1_C2_H4 | chrX | 197717 | 24 | 5 | 100 |
| TY2_D6 | chrII | 36123 | 190 | 95.4 | 10.1 |
| TY2_D6 | chrII | 643722 | 111 | 38.9 | 2.4 |
| TY2_D6 | chrIV | 437349 | 31 | 5.0 | 0.1 |
| TY2_D6 | chrVII | 111132 | 186 | 125.8 | 13.1 |
| TY2_D6 | chrVII | 931306 | 108 | 8.4 | 0.5 |
| TY2_D6 | chrVIII | 91471 | 174 | 43.2 | 4.2 |
| TY2_D6 | chrIX | 196867 | 372 | 63.1 | 13.1 |
| TY2_D6 | chrX | 197644 | 190 | 102.1 | 10.8 |
| TY2_D6 | chrX | 203500 | 190 | 112.3 | 11.9 |
| TY2_D6 | chrXIII | 503871 | 190 | 112.2 | 11.9 |
| TY2_D6 | chrXIII | 808796 | 135 | 8.5 | 0.6 |
| TY2_D6 | chrXIV | 561882 | 186 | 113.0 | 11.8 |
| TY2_D6 | chrXIV | 632367 | 189 | 22.2 | 2.3 |
| TY2_D6 | chrXVI | 437174 | 169 | 36.5 | 3.4 |
| TY2_D6 | chrXVI | 443029 | 173 | 37.6 | 3.6 |
| TY3_B8 | chrIV | 946125 | 2 | 5 | 0.65 |
| TY3_B8 | chrV | 434844 | 90 | 6.74 | 39.75 |
| TY3_B8 | chrV | 435131 | 152 | 5.92 | 58.94 |
| TY3_B8 | chrVIII | 146501 | 2 | 5 | 0.65 |
| TY4_D9 | chrIII | 84346 | 226 | 41.03 | 10 |
| TY4_D9 | chrIII | 90688 | 229 | 29.28 | 7.23 |
| TY4_D9 | chrIII | 93195 | 22 | 5 | 0.12 |
| TY4_D9 | chrIV | 513549 | 83 | 7 | 0.63 |
| TY4_D9 | chrIV | 513788 | 188 | 10.2 | 2.07 |
| TY4_D9 | chrIV | 519602 | 54 | 5 | 0.29 |
| TY4_D9 | chrIV | 645761 | 151 | 8.5 | 1.38 |
| TY4_D9 | chrIV | 651376 | 47 | 5 | 0.25 |
| TY4_D9 | chrIV | 878540 | 144 | 6.11 | 0.95 |
| TY4_D9 | chrIV | 884184 | 51 | 5 | 0.28 |
| TY4_D9 | chrIV | 981295 | 54 | 5 | 0.29 |
| TY4_D9 | chrIV | 987403 | 179 | 7.06 | 1.36 |
| TY4_D9 | chrIV | 992508 | 52 | 5 | 0.28 |
| TY4_D9 | chrV | 62277 | 59 | 5 | 0.32 |
| TY4_D9 | chrV | 135873 | 112 | 6.11 | 0.74 |
| TY4_D9 | chrV | 431705 | 101 | 6.25 | 0.68 |
| TY4_D9 | chrV | 436236 | 92 | 9.89 | 0.98 |
| TY4_D9 | chrV | 487896 | 151 | 8.44 | 1.38 |
| TY4_D9 | chrVI | 137991 | 13 | 5 | 0.07 |
| TY4_D9 | chrVII | 569005 | 10 | 5 | 0.05 |
| TY4_D9 | chrVII | 574456 | 163 | 6.79 | 1.19 |
| TY4_D9 | chrVII | 811557 | 84 | 5 | 0.45 |
| TY4_D9 | chrVII | 817245 | 230 | 6.69 | 1.66 |
| TY4_D9 | chrVIII | 91908 | 206 | 8.4 | 1.87 |
| TY4_D9 | chrVIII | 389432 | 225 | 20.51 | 4.98 |
| TY4_D9 | chrIX | 246400 | 32 | 5 | 0.17 |
| TY4_D9 | chrX | 204038 | 164 | 7.75 | 1.37 |
| TY4_D9 | chrX | 354875 | 221 | 29.6 | 7.06 |
| TY4_D9 | chrX | 538362 | 163 | 34.74 | 6.11 |
| TY4_D9 | chrX | 541347 | 156 | 8.22 | 1.38 |
| TY4_D9 | chrXII | 650891 | 167 | 30.62 | 5.52 |
| TY4_D9 | chrXII | 731858 | 324 | 25.37 | 8.87 |
| TY4_D9 | chrXII | 941445 | 163 | 34.37 | 6.04 |
| TY4_D9 | chrXII | 947074 | 163 | 32.96 | 5.8 |
| TY4_D9 | chrXII | 976494 | 111 | 5 | 0.6 |
| TY4_D9 | chrXIII | 168584 | 278 | 18.17 | 5.45 |
| TY4_D9 | chrXIII | 768594 | 105 | 8.35 | 0.95 |
| TY4_D9 | chrXIV | 562272 | 158 | 8.65 | 1.47 |
| TY4_D9 | chrXIV | 567920 | 166 | 30.89 | 5.53 |
| TY4_D9 | chrXIV | 726843 | 138 | 7.86 | 1.17 |
| TY4_D9 | chrXV | 704315 | 165 | 8.24 | 1.47 |
| TY4_D9 | chrXV | 709881 | 44 | 5 | 0.24 |
| TY4_D9 | chrXVI | 62417 | 207 | 5.81 | 1.3 |
| X2_E11 | chrX | 203508 | 144 | 5.38 | 100 |

Candidate insertion loci were identified by finding reads mapped to a specific chromosomal location whose paired end partner read mapped to a unique region (i.e. a region with no similarity to the yeast genome) on the vector insert. The table indicates all loci identified using this approach, but the candidate loci that either have narrow mapped read stacks or low coverage are not likely to be real insertion loci. The percentage of mapped reads indicates what percentage of reads identified as mapping to candidate insertion loci map to the particular location specified in the table.
