# Supplementary material for: EasyCloneMulti: A Set of Vectors for Simultaneous and Multiple Genomic Integrations in Saccharomyces cerevisiae
Source: PLoS One. 2016 Mar 2;11(3):e0150394. doi: 10.1371/journal.pone.0150394 (PMC4775045; doi:10.1371/journal.pone.0150394)
Supplement: S6 Table — (DOCX) [file pone.0150394.s011.docx]

**Supplementary Table S6: Final biomass concentrations of *S. cerevisiae* strains bearing *T. castaneum panD* on different EasyCloneMulti vectors.**

| **Parent strain** | **Integrated EasyCloneMulti vector** | **Final biomass concentration (gCDW.L^-1^)** |
| --- | --- | --- |
| SCE-iL1-155 | pCfB2099 (Ty1Cons1) | 13.1 ± 0.4 |
| SCE-iL1-155 | pCfB2097 (Ty1Cons2) | 13.4 ± 0.5 |
| SCE-iL1-155 | pCfB2096 (Ty2Cons) | 13.4 ± 0.4 |
| SCE-iL1-155 | pCfB799 (Ty4cons) | 12.8 ± 0.5 |

Final biomass concentrations are the average of biomass concentrations measured for all the transformants belonging to the same strain type after 72h of cultivation in deep well plate using synthetic fed-batch medium (Feed-In-Time fed-batch medium, m2p-Labs). As for indication, the average final biomass concentration, after cultivation in similar conditions, of a strain derived from SCE-iL1-155 but expressing *T. castaneum panD* from a single genomic locus (locus X-2, [1]), was 11.8 ± 0.2 gCDW.L^-1^.

Production of 3HP by the abovementioned strains is reported in figure 6.

1. Mikkelsen MD, Buron LD, Salomonsen B, Olsen CE, Hansen BG, Mortensen UH, et al. Microbial production of indolylglucosinolate through engineering of a multi-gene pathway in a versatile yeast expression platform. Metab Eng. 2012;14: 104–11. doi:10.1016/j.ymben.2012.01.006
